# Development and Verification of an Immune-Based Gene Signature for Risk Stratification and Immunotherapeutic Efficacy Assessment in Gastric Cancer

**DOI:** 10.1155/2021/4251763

**Published:** 2021-11-11

**Authors:** Feng Qiu, Yumei Zhu, Yafeng Shi, Jingjing Ji, Yingchao Jin

**Affiliations:** ^1^Department of Pathology, The Fourth Affiliated Hospital of Harbin Medical University, Harbin, 150001 Heilongjiang, China; ^2^Department of Obstetrics and Gynecology, The Fourth Affiliated Hospital of Harbin Medical University, Harbin, 150001 Heilongjiang, China; ^3^Department of Pathology, The Second Affiliated Hospital of Harbin Medical University, Harbin, 150001 Heilongjiang, China; ^4^Department of General Surgery, Heilongjiang Provincial Hospital, Harbin, 150036 Heilongjiang, China

## Abstract

**Objective:**

Due to the molecular heterogeneity of gastric cancer, only minor patients respond to immunotherapeutic schemes. This study is aimed at developing an immune-based gene signature for risk stratification and immunotherapeutic efficacy assessment in gastric cancer.

**Methods:**

An immune-based gene signature was developed in gastric cancer by LASSO method in the training set. The predictive performance was validated in the external datasets. KEGG pathways related to risk scores were assessed by GSEA. Based on multivariate Cox regression analysis, a nomogram was established. Sensitivity to chemotherapy drugs was evaluated between high- and low-risk samples. The relationships of risk scores with infiltration levels of immune cells, stromal scores, immune scores, immune cell subgroups, and overall response to anti-PD-L1 therapy were determined.

**Results:**

Our results showed that high risk scores were indicative of undesirable survival outcomes both in the training set (*p* < 0.0001) and the validation set (*p* = 0.002). Moreover, this signature could independently predict patients' survival (HR: 2.656 (1.919-3.676) and *p* < 0.001). Subgroup analysis confirmed the sensitivity of this signature in predicting prognosis (all *p* < 0.05). Cancer-related pathways were primarily enriched in high-risk samples, such as MAPK and TGF-*β* pathways (*p* < 0.05). By incorporating stage and the risk score, we established a nomogram for predicting one-, three-, and five-year survival probability. Patients with high-risk scores were more sensitive to chemotherapy drugs (*p* < 0.05). There was heterogeneity in immune cells between high- and low-risk samples (*p* < 0.05). Samples with progressive disease exhibited the highest risk score, and those with complete response had the lowest risk score (*p* < 0.05).

**Conclusion:**

This immune-based gene signature might be representative of a promising prognostic classifier for predicting risk stratification and immunotherapeutic efficacy in gastric cancer, assisting personalized therapy and follow-up plan.

## 1. Introduction

Gastric cancer represents the primary reason for cancer-related deaths globally, despite its declining prevalence in recent years [1–3]. Most of the patients are diagnosed at an advanced stage. The 5-year overall survival (OS) is <40%, and the median survival time after recurrence is simply 8 months [4]. As a heterogeneous malignancy, survival duration widely varies towards subjects with the same clinicopathological characteristics as well as therapeutic schemes. The present staging system alone cannot be predictive of outcomes. Thus, it is of necessity to exploit novel prognostic classifier for predicting risk stratification.

Recently, immunotherapies against CTLA4, PD-1, and PD-L1 inhibitors have displayed efficient therapeutic outcomes for cancer patients [5, 6]. Despite the durable efficacy of immunotherapy against advanced gastric cancer, only minor subjects may respond to this therapy [7, 8]. Because of the extensive molecular heterogeneity of gastric cancer, immunotherapy requires to be made for individual patient, thereby eliciting the optimal therapeutic effects [9]. Increasing evidence underlines the clinical significance of tumor immune microenvironment on immunotherapy [10–12]. Nevertheless, there is lack of immune-related signatures for predicting which gastric cancer patients will respond to immunotherapy. Here, this study developed and externally verified an immune-based gene signature for gastric cancer, which may become a promising clinical tool for risk stratification and immunotherapeutic efficacy prediction in gastric cancer.

## 2. Materials and Methods

### 2.1. Data Collection

Level 3 transcriptome data (HTSeq-FPKM) and clinical information of gastric cancer patients were downloaded from The Cancer Genome Atlas (TCGA) database via the Genomic Data Commons (https://portal.gdc.cancer.gov). Furthermore, the gene expression profiles and corresponding clinical data of gastric cancer were retrieved from the GSE66229 dataset of the Gene Expression Omnibus (GEO) database (https://www.ncbi.nlm.nih.gov/geo/) [13], which were background corrected and normalized by quartile through robust multiarray average algorithm. If a gene symbol corresponded to multiple probes, the mean value was utilized as its expression value. Gene expression data of gastric cancer were also obtained from an immunotherapy cohort (Imvigor210) [14]. Exclusion criteria of our study population were as follows: (1) patients with survival time of 0; (2) patients with incomplete clinical information. TCGA dataset (*n* = 350) was applied as the training set, while the GSE66229 (*n* = 300) and Imvigor210 (*n* = 298) datasets were utilized as the validation set.

### 2.2. Differential Expression Analysis of Immune-Related Genes (IRGs)

IRGs were extracted from the ImmPort database (https://immport.niaid.nih.gov). Then, IRGs were overlapped from TCGA, GSE66229, and Imvigor210 datasets for next analyses (Supplementary Table 1). Differentially expressed IRGs with ∣log fold change (FC) | >1 and false discovery rate (FDR) < 0.05 were screened between gastric cancer and normal tissues in TCGA dataset utilizing the limma package [15].

### 2.3. Functional Annotation Analysis

Biological functions of differentially expressed IRGs were annotated through the clusterProfiler package, containing Gene Ontology (GO) and Kyoto Encyclopedia of Genes and Genomes (KEGG) pathway enrichment analyses [16]. GO terms were composed of biological process (BP), cellular component (CC), and molecular function (MF). Terms with FDR < 0.05 were indicative of significant enrichment.

### 2.4. Construction of an Immune-Related Gene Model

The correlations between IRGs and prognosis of gastric cancer were assessed in the training set utilizing the coxph package. Genes with *p* value < 0.05 were chosen as prognosis-related IRGs. The least absolute shrinkage and selector operation (LASSO) regression analysis was conducted by applying the glmnet package, followed by tenfold cross-verification [17]. The risk score was determined according to regression coefficient and expression of specified IRGs. The formula of risk score = risk score = ∑(regression coefficient of gene × expression of signature gene). Based on the median value of risk scores, subjects were divided into high- and low-risk subgroups. Kaplan-Meier curves of overall survival (OS) were conducted via the survival package, followed by log-rank test. Receiver operating characteristic (ROC) curves of one-, three-, and five-year survival duration were established by the survivalROC package. Furthermore, we compared the predictive efficacy of this signature with the immune-related prognostic signatures constructed by Li and He [18] and Tian et al. [19] by ROC curves. Univariate Cox regression analysis was carried out to determine the associations of survival duration with age, gender, grade, stage, TNM, and risk score according to hazard ratio (HR), 95% confidence interval (CI), and *p* value. Afterwards, multivariate Cox regression analysis was presented for evaluating whether these variables independently predicted the prognosis.

### 2.5. Subgroup Analysis

Patients were separated into different subgroups according to age (age > 65 and age < 65), gender (female and male), grade (G1-2 and G3), T (T1-2 and T3-4), N (N0 and N1-3), M (M0 and M1), and stage (stage I-II and stage III-IV). In different subgroups, Kaplan-Meier curves of OS were implemented between high- and low-risk patients.

### 2.6. Gene Set Enrichment Analysis (GSEA)

GSEA was employed to probe KEGG pathways positively correlated to high- or low-risk scores. The gene sets were retrieved from the Molecular Signatures Database [20]. The number of permutations was set as 1000, and pathways with FDR < 0.25 were identified.

### 2.7. Nomogram

After determining independent prognostic factors, this study constructed a nomogram for predicting one-, three-, and five-year survival duration. The efficacy of this model was under evaluation by ROCs of one-, three-, and five-year survival duration. Moreover, calibration plots were depicted for comparing the model-predicted one-, three-, and five-year survival with the actual survival probability by employing the rms package.

### 2.8. Analysis of Sensitivity to Chemotherapy Drugs

This study assessed the sensitivity of gastric cancer samples in the training set to chemotherapy drugs by the Genomics of Drug Sensitivity in Cancer (GDSC; https://www.cancerrxgene.org/) database that is the largest public resource for drug sensitivity in cancer cells and molecular biomarkers of drug responses [21]. Furthermore, the half-maximal inhibitory concentration (IC50) values were calculated via the pRRophetic package [22].

### 2.9. Connectivity Map (CMap)

Abnormally expressed genes were screened between high- and low-risk subgroups in the training set by applying the limma package [15]. The criteria were as follows: ∣FC | >1.5 and FDR < 0.05. Based on the up- and downregulated genes, underlying small molecule compounds were predicted through the CMap (http://portals.broadinstitute.org/cmap/) database that is a tool that has been widely applied for studying drug repositioning as well as side effect prediction [23]. Shared mechanisms of action were evaluated by employing mode-of-action analyses.

### 2.10. Genetic Mutation Analysis

Somatic mutation data of 437 gastric cancer samples were obtained from TCGA database. The mutation types and frequencies were determined through the MutSigCV algorithm [24]. The top five mutation genes were extracted, and patients were separated into wild-type and mutation subgroups. The predictive efficacy of this signature was assessed in each subgroup.

### 2.11. CIBERSORT

CIBERSORT (http://cibersort.stanford.edu/) is an algorithm to characterize cell compositions of complex tissues based on gene expression profiles [25]. CIBERSORT tool was employed to infer the composition ratio of 22 tumor-infiltrating immune cells in gastric cancer samples through deconvolution algorithm.

### 2.12. Estimation of Stromal and Immune Cells in Malignant Tumors Using Expression Data (ESTIMATE)

ESTIMATE can use gene expression signatures to infer the fractions of stromal and immune cells in tumor tissues [26]. This study evaluated the stromal scores and immune scores between the high- and low-risk gastric cancer groups based on gene expression profiles through the ESTIMATE package.

### 2.13. Immunohistochemistry

Paraffin-embedded sections of 5 paired gastric cancer and adjacent normal tissues were collected from Heilongjiang Provincial Hospital. Each patient signed a written informed statement. This study gained the approval of the Ethics Committee of Heilongjiang Provincial Hospital (2020061). All specimens were fixed through 10% formalin for 48 h and sectioned into 5 *μ*m thickness. The sections were incubated by primary antibodies against APOD (1/100; ab108191; Abcam, USA), CTLA4 (1/100; ab237712), CXCR4 (1/100; ab197203), DKK1 (1/100; ab109416), INHBA (1/100; ab97705), NPR1 (1/100; ab40817), PENK (1/100; ab22619), PROC (1/100; ab17771), RBP4 (1/100; ab188230), S100A12 (1/100; ab196740), and STC1 (1/100; ab229477) overnight at 4°C and incubated by HRP-labeled secondary antibodies for 30 min at room temperature. Afterwards, the sections were stained by hematoxylin and investigated under a microscope.

### 2.14. Statistical Analysis

Statistical analysis was carried out by applying R 3.6.3 (https://www.r-project.org/). Comparisons between two groups were analyzed by the Wilcoxon test. Multiple comparisons were assessed through the Kruskal-Wallis test. *p* value < 0.05 was indicative of statistical significance.

## 3. Results

### 3.1. Immune-Related Genes in Gastric Cancer

**Figure 1:**
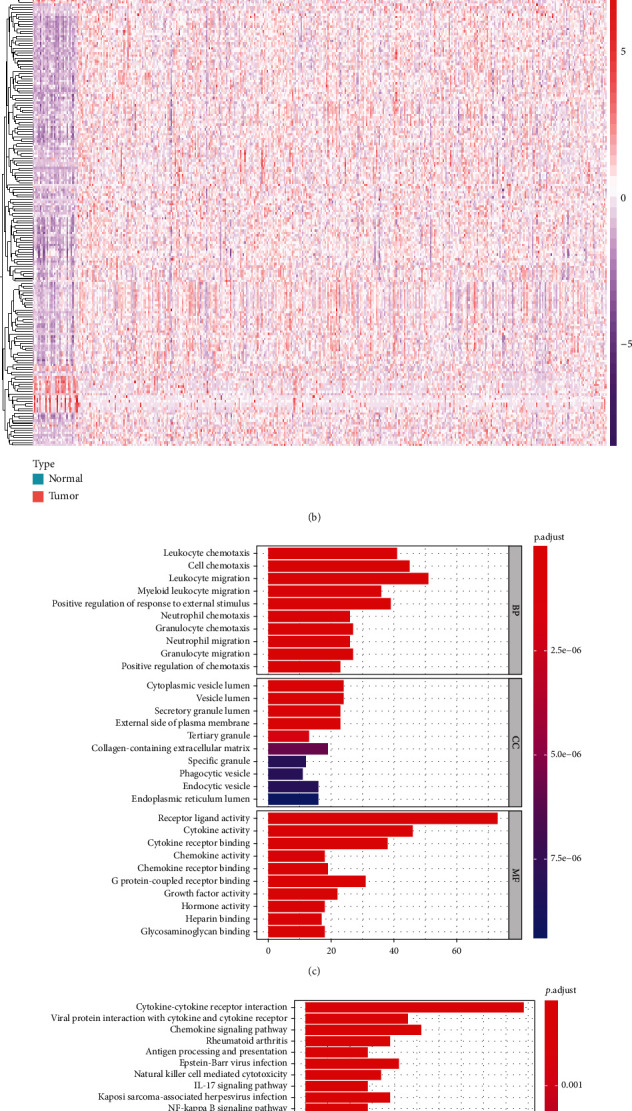
Differentially expressed immune-related genes and their immune functions in gastric cancer in TCGA dataset. (a) Volcano plot for visualizing the expression of immune-related genes in gastric cancer and normal samples. Red dots indicate upregulation, green dots indicate downregulation, and black dots indicate nonsignificance. (b) Hierarchical clustering analysis of differentially expressed immune-related genes in gastric cancer and normal tissues. (c) GO and (d) KEGG enrichment analysis of differentially expressed immune-related genes.

216 IRGs were differentially expressed in gastric cancer compared to normal samples from TCGA dataset. The detailed information of these IRGs is listed in Supplementary Table 2. Among them, 50 IRGs were downregulated and 166 were upregulated in gastric cancer (Figures 1(a) and 1(b)). Functional enrichment analysis confirmed their complex immune functions (Figures 1(c) and 1(d)). Various immune pathways were significantly enriched like chemokine, cytokine, antigen processing and presentation, IL-17 pathways.

### 3.2. Establishment of an Immune-Related Prognostic Signature for Gastric Cancer

**Figure 2:**
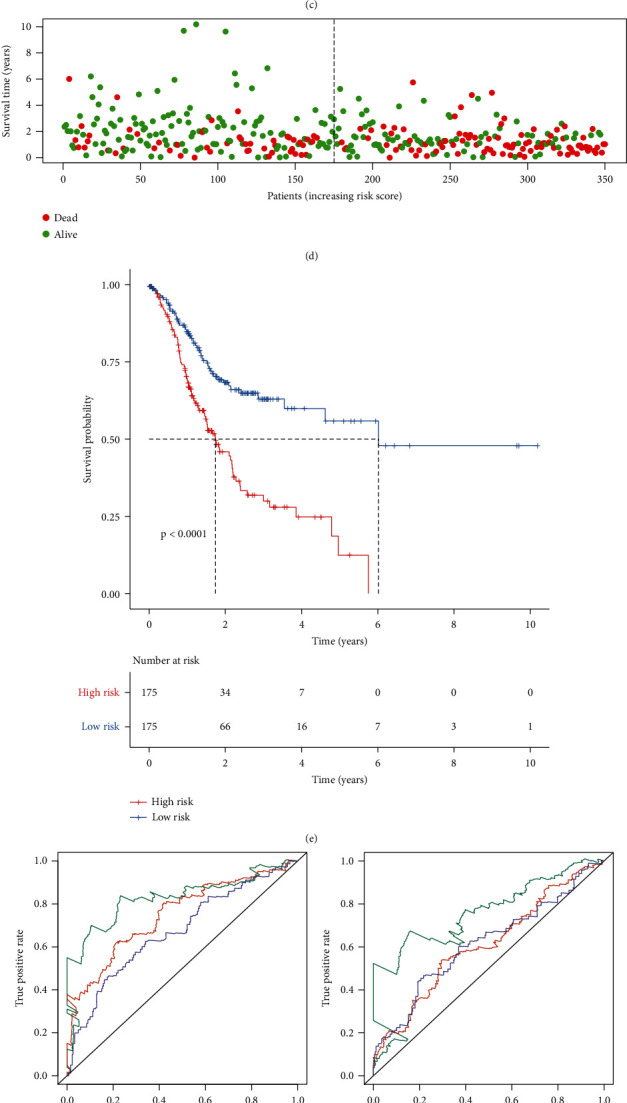
Establishment of an immune gene-based signature for predicting prognosis of gastric cancer in the training set. (a) Selection of variables in LASSO regression model by tenfold cross-verification. (b) Partial likelihood deviance for each *λ* in LASSO regression model. (c) Ranking of risk scores for gastric cancer patients. (d) Survival status of each patient. (e) Kaplan-Meier curves for OS between the high- and low-risk groups. (f) ROCs of one-, three-, and five-year survival based on this signature. ROCs of one-, three-, and five-year survival based on the immune-related signature constructed by (g) Li et al. and (h) Tian et al. (i) Univariate and (j) multivariate Cox regression analysis of the risk score and other clinicopathological characteristics.

We firstly screened 23 prognosis-related IRGs for gastric cancer with *p* value < 0.05 (Table 1). Then, a LASSO regression model was constructed based on the 13 prognostic IRGs (Figures 2(a) and 2(b)). The risk score was calculated, as follows: 0.0163595534614718 ∗ INHBA expression + 0.112976565411344 ∗ STC1 expression + 0.0939280691660978 ∗ NRP1 expression + (−0.2540793422499) ∗ CTLA4 expression + 0.0724948304927518 ∗ GCG expression + 0.0536048393713446 ∗ RNASE2 + 0.0187605064028966 ∗ PENK + 0.190660002104556 ∗ CXCR4 + 0.0522712129657809 ∗ S100A12 expression + 0.12694740939673 ∗ PROC expression + 0.0631625200474562 ∗ DKK1 expression + 0.00494872423672059 ∗ RBP4 expression + 0.0414384058727867 ∗ APOD expression. Patients were separated into high- and low-risk groups in line with the median value of risk scores (Figure 2(c)). We found that in the high-risk group, there were more patients with dead status compared to the low-risk group (Figure 2(d)). Subjects with high-risk scores exhibited pessimistic clinical outcomes in comparison to those with low-risk scores (*p* < 0.0001; Figure 2(e)). The area under the curves (AUCs) of one year, three years, and five years were separately 0.671, 0.748, and 0.827 (Figure 2(f)). Compared to the immune-related prognostic signatures constructed by Li and He [18] (Figure 2(g)) and Tian et al. [19] (Figure 2(h)), this signature had higher predictive efficacy towards gastric cancer prognosis. The correlations of risk score with clinical characteristics were evaluated among gastric cancer subjects. As a result, this risk score displayed distinct associations with stage (*p* = 0.0106) and T (*p* = 0.0272) in gastric cancer (Table 2). Univariate Cox regression analysis demonstrated that age (*p* = 0.033, HR: 1.021; 95% CI: 1.002-1.042), stage (*p* = 0.002, HR: 1.465; 95% CI: 1.154-1.861), N (*p* = 0.022, HR: 1.235; 95% CI: 1.031-1.478), and risk score (*p* < 0.001, HR: 2.656; 95% CI: 1.919-3.676) were risk factors of gastric cancer prognosis (Figure 2(i)). To verify their independency of predicting prognosis, multivariate Cox regression analysis was presented. In Figure 2(j), age (*p* = 0.003, HR: 1.032; 95% CI: 1.011-1.053), stage (*p* = 0.009, HR: 1.527; 95% CI: 1.112-2.097), and risk score (*p* < 0.001, HR: 2.861; 95% CI: 2.018-4.056) independently predicted the clinical outcomes of gastric cancer.

### 3.3. External Validation of the Immune-Related Prognostic Signature for Gastric Cancer

**Figure 3:**
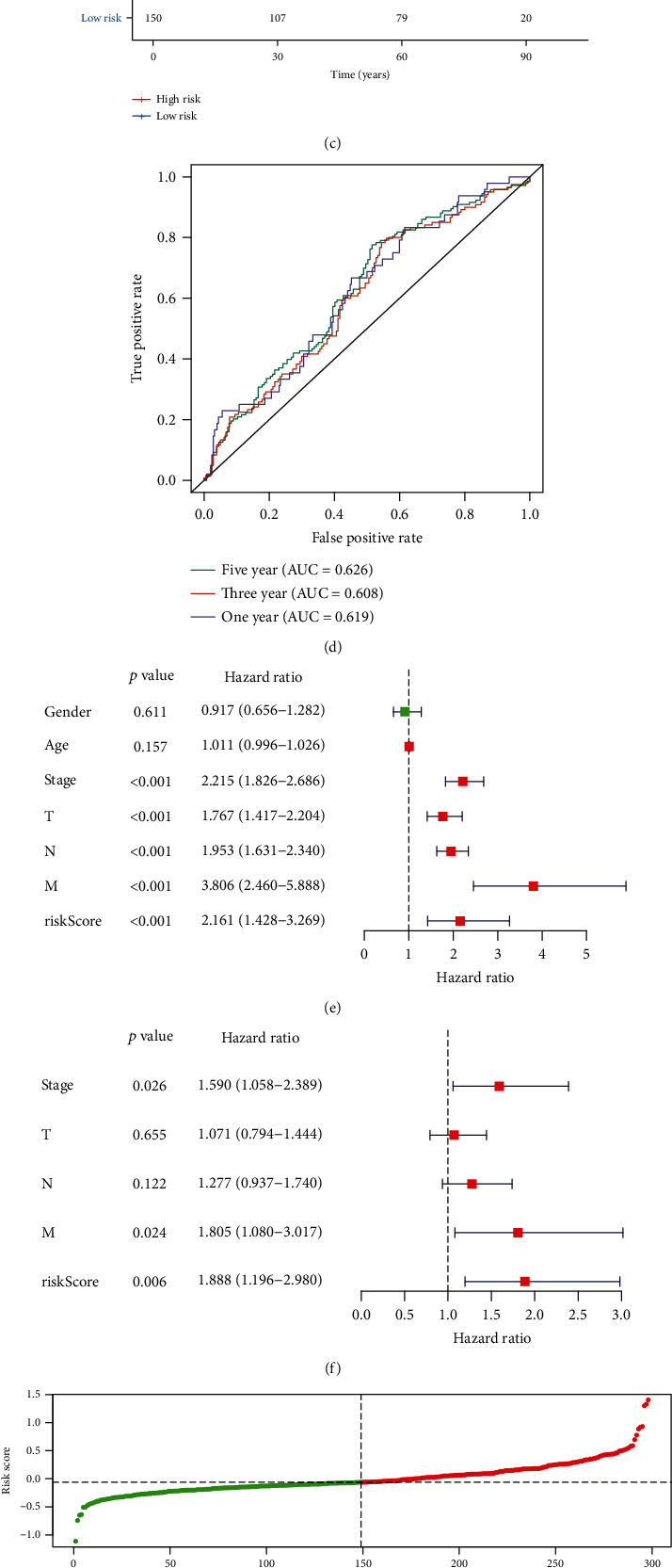
External validation of the immune-related prognostic signature for gastric cancer. (a) Ranking of risk scores and (b) survival status for patients in the GSE66229 dataset. (c) Kaplan-Meier curves for OS between the high- and low-risk groups in the GSE66229 dataset. (d) ROCs for one year, three years, and five years in the GSE66229 dataset. (e) Univariate and (f) multivariate Cox regression analyses of the risk score and other clinical characteristics in the GSE66229 dataset. (g) Ranking of risk scores and (h) survival status for patients in the Imvigor210 dataset. (i) Kaplan-Meier curves for OS between the high- and low-risk groups in the Imvigor210 dataset.

To verify the predictive performance of this risk score, we employed the GSE66229 and Imvigor210 datasets. According to the formula of risk score, we calculated the risk score of each patient in the GSE66229 dataset. Patients were divided into the high- and low-risk groups on the basis of the median value of risk scores (Figures 3(a) and 3(b)). High risk scores were indicative of undesirable prognosis for gastric cancer patients (*p* = 0.002; Figure 3(c)). The AUCs of one year, three years, and five years were separately 0.619, 0.608, and 0.626 (Figure 3(d)), confirming the well performance for predicting patients' survival outcomes. In Table 3, the risk score exhibited significant associations with stage (*p* = 0.0073), T (*p* < 0.0001), and M (*p* = 0.0437) among patients. According to univariate Cox regression analysis, stage (*p* < 0.001, HR: 2.215; 95% CI: 1.826-2.686), T (*p* < 0.001, HR: 1.767; 95% CI: 1.417-2.204), N (*p* < 0.001, HR: 1.953; 95% CI: 1.631-2.340), M (*p* < 0.001, HR: 3.806; 95% CI: 2.460-5.888), and risk score (*p* < 0.001, HR: 2.161; 95% CI: 1.428-3.269) could be risk factors of gastric cancer (Figure 3(e)). Following validation using multivariate Cox regression analysis, stage (*p* = 0.026, HR: 1.590; 95% CI: 1.058-2.389), M (*p* = 0.024, HR: 1.805; 95% CI: 1.080-3.017), and risk score (*p* = 0.006, HR: 1.888; 95% CI: 1.196-2.980) were independently related to poor prognosis (Figure 3(f)). In the Imvigor210 dataset, patients were divided into high- and low-risk groups according to the median value (Figures 3(g) and 3(h)). After validation, high risk scores were indicative of awful prognosis for gastric cancer patients (*p* < 0.0001; Figure 3(i)). By external validation, this signature was a promising tool for predicting prognosis of gastric cancer.

### 3.4. The Immune-Related Signature Can Be Predictive of Survival for Gastric Cancer with Different Clinicopathological Features

**Figure 4:**
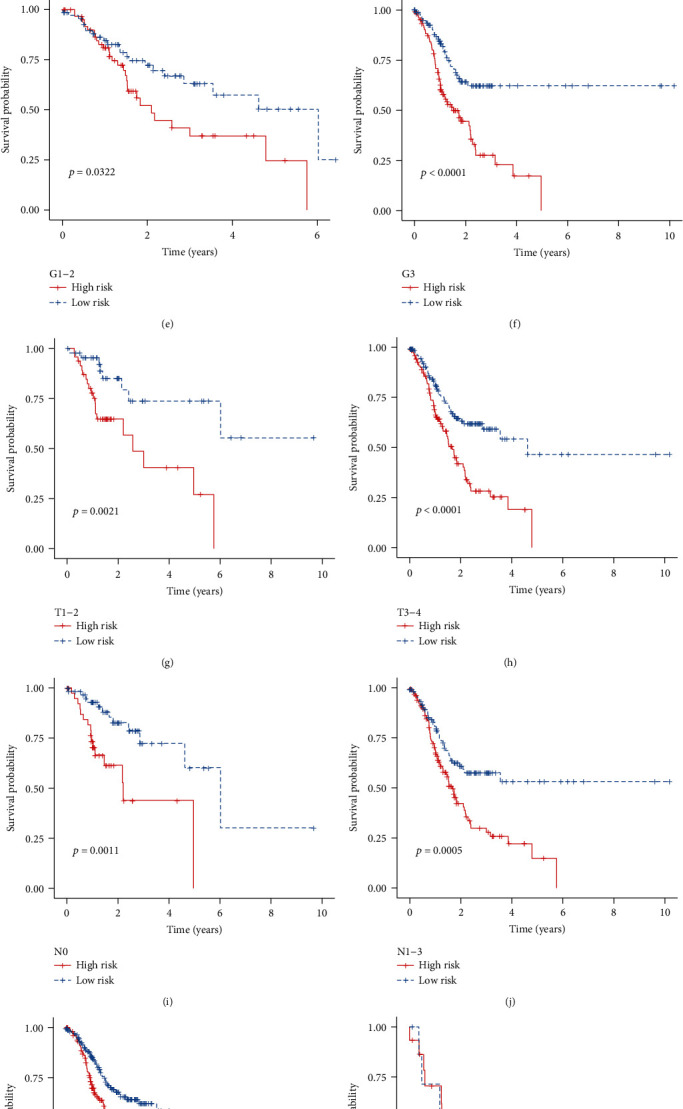
The immune-related signature can be predictive of survival of gastric cancer patients with different clinicopathological features. Kaplan-Meier curves of OS between the high- and low-risk groups for patients with (a) age > 65 and (b) age < 65; (c) female and (d) male; (e) G1-2 and (f) G3; (g) T1-2 and (h) T3-4; (i) N0 and (j) N1-3; (k) M0 and (l) M1; (m) stage I-II and (n) stage III-IV.

To detect the sensitivity of this signature for predicting prognosis, we presented Kaplan-Meier curves of OS between high- and low-risk gastric cancer groups in different subgroups. Our data showed that patients with high risk scores exhibited poorer survival duration in comparison to those with low risk scores in age > 65 (*p* = 0.0011, Figure 4(a)) and age < 65 (*p* < 0.0001, Figure 4(b)); female (*p* = 0.0001, Figure 4(c)) and male (*p* = 0.0005, Figure 4(d)); G1-2 (*p* = 0.0322, Figure 4(e)) and G3 (*p* < 0.0001, Figure 4(f)); T1-2 (*p* < 0.0001, Figure 4(g)) and T3-4 (*p* = 0.0021, Figure 4(h)); N0 (*p* = 0.0011, Figure 4(i)) and N1-3 (*p* = 0.0005, Figure 4(j)); M0 (*p* < 0.0001, Figure 4(k)) and M1 (*p* = 0.1664, Figure 4(l)); stage I-II (*p* = 0.0002, Figure 4(m)) and stage III-IV (*p* = 0.0028, Figure 4(n)).

### 3.5. Signaling Pathways Related to the Risk Score

**Figure 5:**
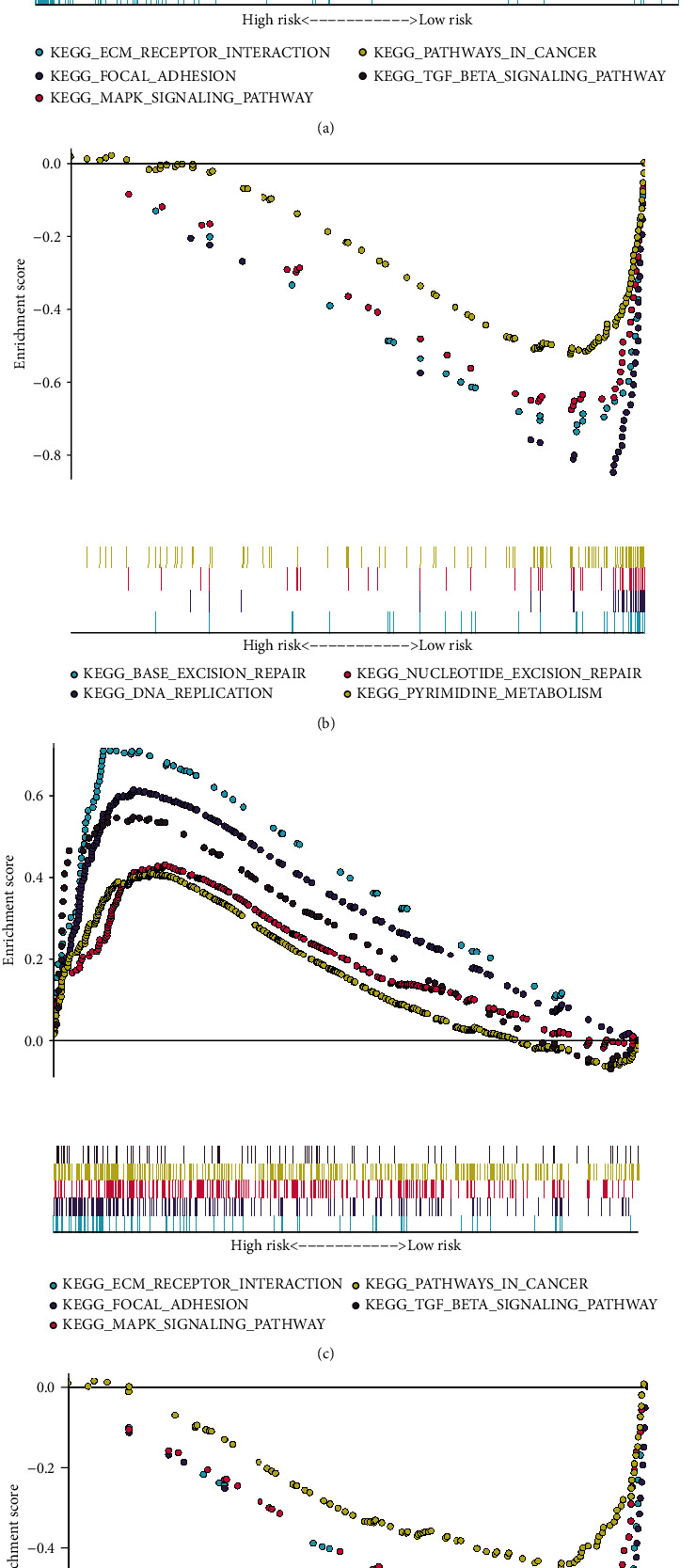
KEGG signaling pathways related to the risk score by GSEA. (a, b) Enriched signaling pathways in the high- and low-risk gastric cancer samples in the training set. (c, d) Enriched signaling pathways in the high- and low-risk gastric cancer samples in the GSE66229 dataset.

The relationships of the risk score and signaling pathways were investigated by GSEA. In the training set, ECM receptor interaction, focal adhesion, MAPK signaling pathway, pathways in cancer, and TGF-*β* signaling pathway were positively related to the high-risk scores (Figure 5(a)). Meanwhile, base excision repair, DNA replication, nucleotide excision repair, and pyrimidine metabolism were enriched in low-risk samples (Figure 5(b)). Above results were confirmed in the GSE66229 dataset (Figures 5(c) and 5(d)).

### 3.6. Construction of a Nomogram Based on the Risk Score for Gastric Cancer

**Figure 6:**
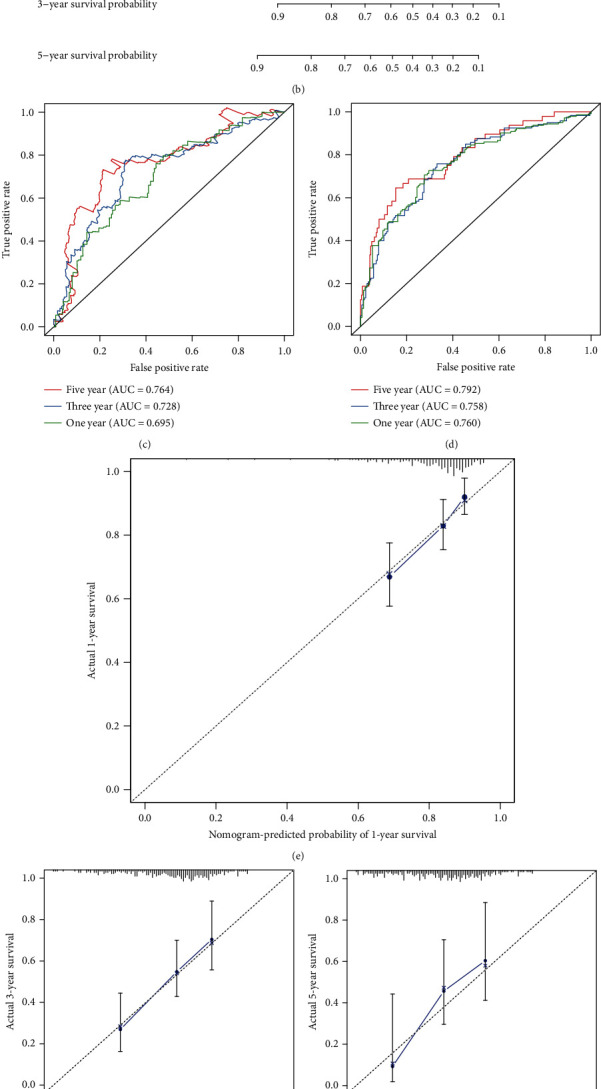
Establishment of a risk score-based nomogram for predicting prognosis of gastric cancer. The nomogram that integrates the risk score and stage for predicting one-, three-, and five-year survival probability in the (a) training set and the (b) GSE66229 dataset. ROCs of one-, three-, and five-year survival based on the nomogram in (c) the training set and (d) the GSE66229 dataset. Calibration curves showing the relationships between nomogram-predicted one-, three-, and five-year survival and actual survival duration in (e–g) the training set and (h–j) the GSE66229 dataset.

In the training set, we established a nomogram for predicting one-, three-, and five-year survival by integrating independent variables including stage and the risk score (Figure 6(a)). The *C*-index was 0.683 in the training set and 0.711 in the GSE66229 dataset (Figure 6(b)). AUCs of one year, three years, and five years were separately 0.695, 0.728, and 0.764 in the training set, showing that the nomogram exhibited better predictive performance (Figure 6(c)). In the GSE66229 dataset, AUCs of one year, three years, and five years were 0.760, 0.758, and 0.792, respectively (Figure 6(d)). Calibration curves confirmed that nomogram-predicted one- (Figure 6(e)), three- (Figure 6(f)), and five-year (Figure 6(g)) survival was highly consistent with the actual one-, three-, and five-year survival. The similar consequences were observed in the GSE66229 dataset (Figures 6(h)–6(j)). Thus, this nomogram displayed the well predictive performance.

### 3.7. Assessment of Sensitivity to Chemotherapy Drugs and Prediction of Small Molecular Compounds Based on the Risk Score

**Figure 7:**
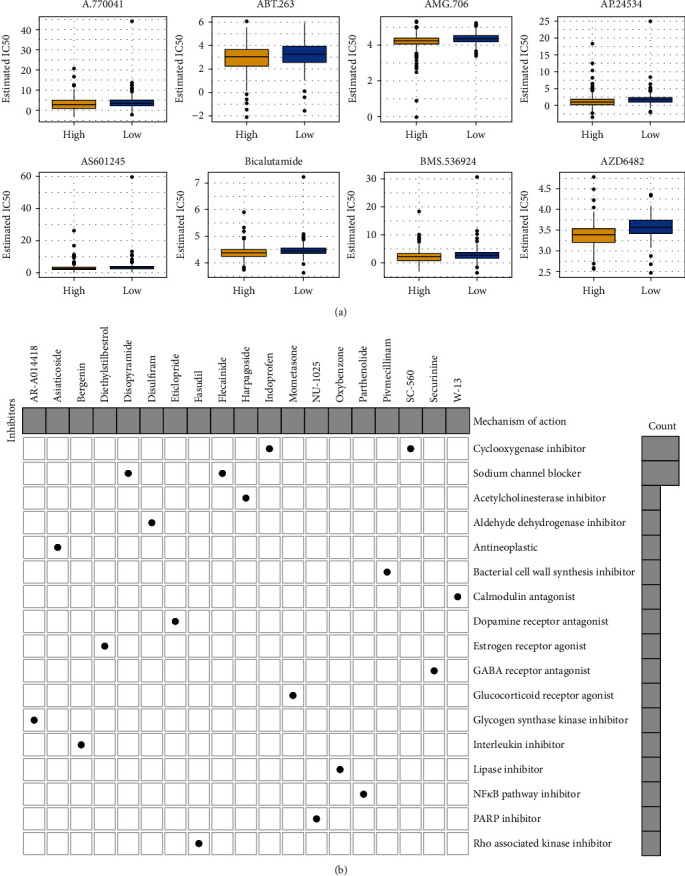
Assessment of sensitivity of chemotherapy drugs and prediction of small molecular compounds based on the risk score. (a) Box plots for estimated IC50 values of A.770041, ABT.263, AMG.706, AP.24534, AS601245, Bicalutamide, BMS.536924, and AZD6482 in high- and low-risk gastric cancer samples. (b) Mechanisms of action shared by small molecular inhibitors.

A.770041 (*p* = 0.001116), ABT.263 (*p* = 0.007174), AMG.706 (*p* = 5.61*E* − 06), AP.24534 (*p* = 3.16*E* − 08), AS601245 (*p* = 9.27*E* − 05), Bicalutamide (*p* = 4.51*E* − 05), BMS.536924 (*p* = 0.002514), and AZD6482 (*p* = 4.55*E* − 12) exhibited higher estimated IC50 values in the low-risk samples compared to the high-risk samples from the training set (Figure 7(a)), indicating that high-risk samples were more sensitive to these chemotherapy drugs. With the criteria of ∣FC | >1.5 and adjusted *p* < 0.05, we screened 33 downregulated and 1484 upregulated genes in high-risk compared to low-risk gastric cancer samples (Supplementary Table 3). Based on these genes, we predicted potential small compounds through the CMap database. In Table 4, 26 small molecular drugs were listed with *p* < 0.05. Furthermore, we analyzed the shared mechanisms of action of predicted small molecular compounds (Figure 7(b)). Cyclooxygenase inhibitor was shared by indoprofen and SC-560. Sodium channel blocker was shared by disopyramide and flecainide.

### 3.8. The Risk Score Can Predict Prognosis of Gastric Cancer with Different Gene Mutations

**Figure 8:**
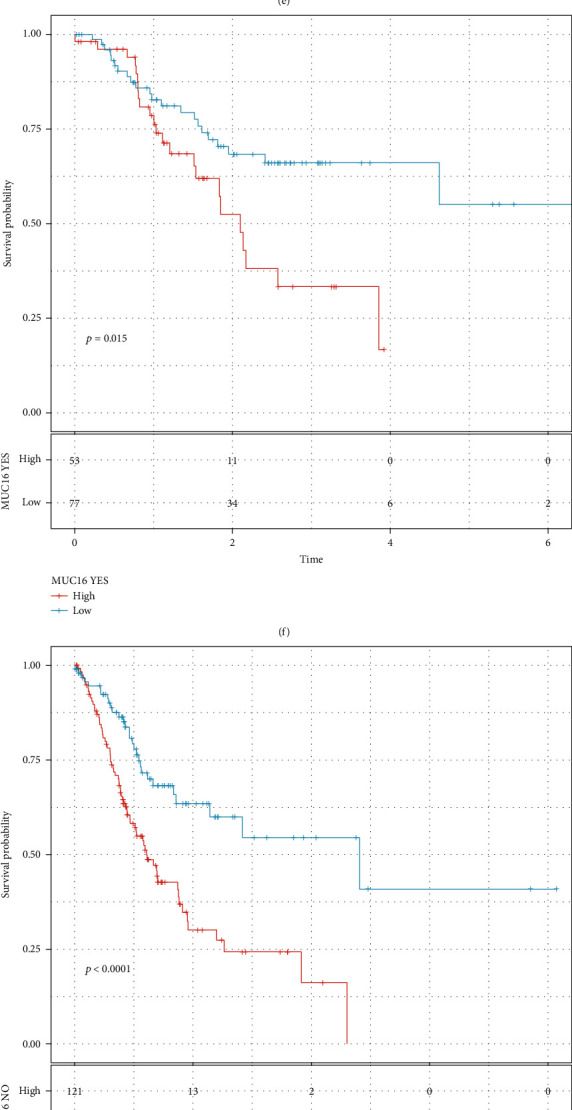
The risk score can be predictive of prognosis of gastric cancer patients with different gene mutations. (a) Landscape of gene variations in gastric cancer samples. The mutation type is identified by a unique color. Kaplan-Meier OS curves of high- and low-risk gastric cancer patients in different subgroups of (b) TP53 mutation and (c) TP53 wild-type; (d) TTN mutation and (e) TTN wild-type; (f) MUC16 mutation and (g) MUC16 wild-type; (h) LRP1B mutation and (i) LRP1B wild-type; (j) SYNE1 mutation and (k) LRP1B wild-type.

We assessed gene variations in gastric cancer samples. As a result, we found that 405 sample mutations occurred among 437 samples (92.68%) in Figure 8(a). TTN (53%), TP53 (46%), MUC16 (32%), LRP1B (27%), and SYNE1 (25%) were the top five mutated genes. The predictive efficacy of this signature was further evaluated in mutant and wild-type gastric cancer samples. Our data demonstrated that patients with high risk scores were indicative of undesirable survival duration in comparison to those with low risk scores in different subgroups including TP53 mutation (*p* < 0.0001, Figure 8(b)) and TP53 wild-type (*p* = 0.0018, Figure 8(c)); TTN mutation (*p* < 0.0001, Figure 8(d)) and TTN wild-type (*p* = 0.0025, Figure 8(e)); MUC16 mutation (*p* = 0.0015, Figure 8(f)) and MUC16 wild-type (*p* < 0.0001, Figure 8(g)); LRP1B mutation (*p* = 0.00067, Figure 8(h)) and LRP1B wild-type (*p* = 0.00021, Figure 8(i)); SYNE1 mutation (*p* = 0.025, Figure 8(j)) and LRP1B wild-type (*p* < 0.0001, Figure 8(k)).

### 3.9. The Risk Score Can Be Predictive of Immunotherapy Efficacy

**Figure 9:**
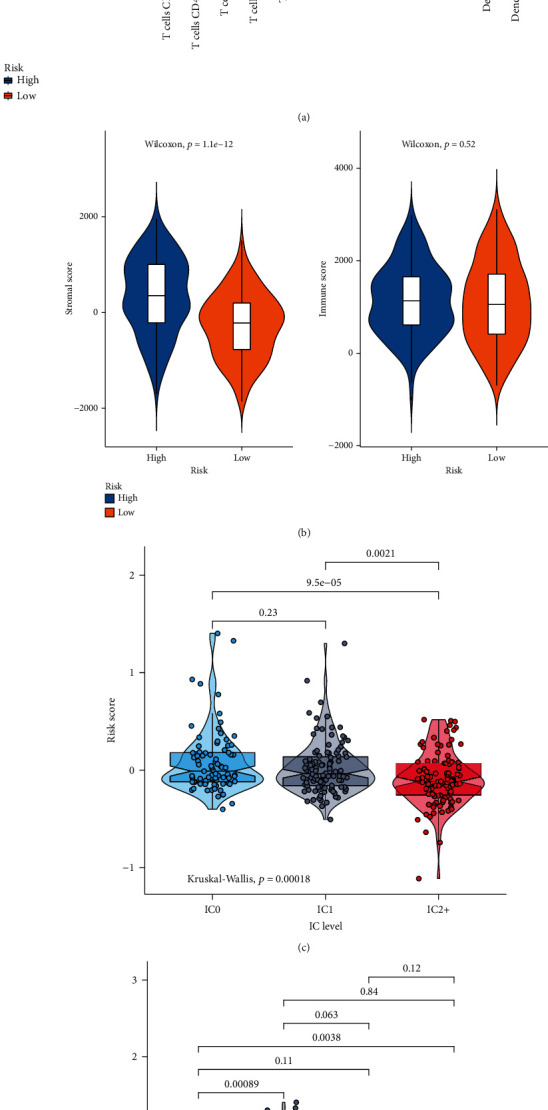
Assessment of the risk score in predicting the efficacy of immunotherapy. (a) Box plots for the relationships of the risk score with infiltration levels of immune cells in gastric cancer samples. ^∗^*p* < 0.05; ^∗∗^*p* < 0.01; ^∗∗∗^*p* < 0.001; ns: not significant. (b) Violin plots for the associations of the risk score with stromal and immune scores. (c) Violin plots for the risk scores in different immune cell subgroups (IC0, IC1, and IC2+) in the Imvigor210 dataset. (d) Violin plots for the relationships of the risk core with immunotherapy efficacy (CR: complete response; PD: progressive disease; PR: partial response; SD: stable disease).

The relationships between the risk score and the infiltration of immune cells were evaluated in gastric cancer samples. High risk scores were characterized by increased infiltration levels of B cells naïve, T cells CD4 memory resting, monocytes, and macrophages M2 (Figure 9(a)). Meanwhile, low risk scores had the characteristics of elevated infiltration levels of T cells CD8, T cells CD4 memory activated, T cells follicular helper, and macrophages M1. Furthermore, stromal scores were significantly elevated in the high-risk group compared to the low-risk group (*p* = 1.1*e* − 12; Figure 9(b)). However, there were no significant differences in immune scores between the high- and low-risk groups (*p* = 0.52). Then, we assessed whether this risk score can be applied to predict the efficacy of anti-PD-L1 immunotherapy in the Imvigor210 dataset. Our data showed that this risk score had the distinct associations with immune cell (IC) subgroups (*p* = 0.00018; Figure 9(c)). Among them, IC2+ cells had the lowest risk score. Moreover, we found that the risk score was in relation to overall responses to the anti-PD-L1 immunotherapy (*p* = 0.0035; Figure 9(d)). Samples with progressive disease exhibited the highest risk score, and those with complete response had the lowest risk score. Collectively, this immune-related signature can be utilized to assess immunotherapy efficacy and predict which patients could benefit from immunotherapy.

### 3.10. Validation of Genes in the Prognostic Model in Gastric Cancer

**Figure 10:**
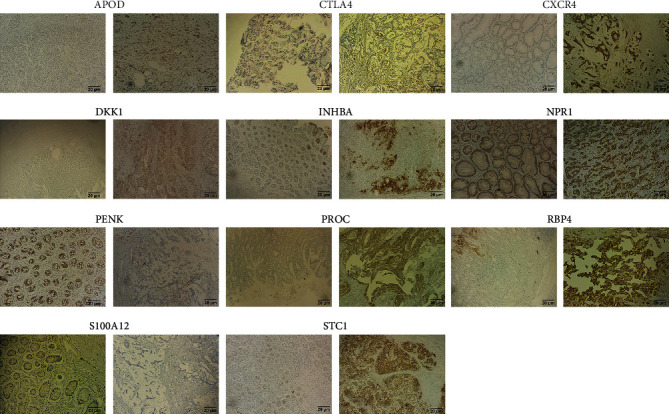
Immunohistochemistry for detecting the expression of APOD, CTLA4, CXCR4, DKK1, INHBA, NPR1, PENK, PROC, RBP4, S100A12, and STC1 in 5 paired gastric cancer and normal tissues. Bar = 20 *μ*m. Magnification, ×200.

Immunohistochemistry was presented for validating the expression of genes in the prognostic model in 5 paired gastric cancer and normal tissues. Our data confirmed that APOD, CTLA4, CXCR4, DKK1, INHBA, NPR1, PENK, PROC, RBP4, S100A12, and STC1 were abnormally expressed in gastric cancer compared to normal tissues (Figure 10).

## 4. Discussion

Gastric cancer, a heterogeneous malignancy, is characterized by diverse molecular and histological subtypes [27]. Immunotherapy may exert durable efficacy against advanced gastric cancer. However, only minor patients benefit from this therapy. Here, this study developed an immune-based gene signature for gastric cancer, which may be utilized for risk stratification and predictive of the response to immunotherapy. This prognostic classifier might possess the potential to assist oncologists make personalized therapeutic scheme and follow-up plan.

Dysregulation of gene expression that is modulated by various regulators may induce human malignancies [28]. Gene expression profiles of immune signatures within gastric cancer could discover markers of immunotherapy as well as survival outcomes. This study comprehensively analyzed abnormally expressed IRGs in gastric cancer. Our functional enrichment analysis confirmed their complex immune functions. Based on LASSO method, an immune-based gene signature was established, containing INHBA, STC1, NRP1, CTLA4, GCG, RNASE2, PENK, CXCR4, S100A12, PROC, DKK1, RBP4, and APOD. Compared with the immune-related prognostic signatures constructed by Li and He [18] and Tian et al. [19], this signature exhibited higher predictive efficacy for gastric cancer prognosis. Following external verification, high risk scores were indicative of undesirable survival outcomes. Our multivariable Cox regression and subgroup analyses confirmed the independency of this signature as a risk factor. We analyzed the biological functions behind the model in more depth. Our GSEA results demonstrated that ECM receptor interaction, focal adhesion, MAPK signaling pathway, pathways in cancer, and TGF-*β* signaling pathway were positively related to the high-risk scores while base excision repair, DNA replication, nucleotide excision repair, and pyrimidine metabolism were enriched in low-risk samples, indicating that varying prognosis among patients might be related to pathways. For example, ECM may provide support as well as maintain normal epithelial architecture [29]. Yang et al. found that ECM receptor interaction signatures such as CD36, COL5A2, and ITGB5 displayed distinct correlations to clinical outcomes of gastric cancer subjects [30]. Our data demonstrated that stage and the risk score were independent risk factors for gastric cancer, which were incorporated into the nomogram. Following verification, this nomogram could provide personalized prediction for one-, three-, or five-year survival duration.

The present staging system alone cannot be predictive of which patients with stage II or III could benefit from adjuvant chemotherapy [31]. This signature could be utilized for predicting the sensitivity to chemotherapy drugs. Patients with high risk scores were more likely to benefit from A.770041, ABT.263, AMG.706, AP.24534, AS601245, Bicalutamide, BMS.536924, and AZD6482 adjuvant chemotherapy. Furthermore, based on the risk score, we probed the underlying small molecular compounds against gastric cancer such as indoprofen, SC-560, disopyramide, and flecainide. Their therapeutic effects are worth exploring further. Genetic mutation frequently occurred in gastric cancer. Among 437 gastric cancer samples, 92.68% different types of mutations occurred. The most common mutation genes were TTN (53%), TP53 (46%), MUC16 (32%), LRP1B (27%), and SYNE1 (25%). We found that both in wild-type and mutation subgroups, this signature can be accurately predictive of subjects' outcomes, confirming its stability and extensibility.

Cell ingredients in the tumor microenvironment display key clinicopathologic implications for prediction of prognosis as well as therapeutic efficacy in gastric cancer [12]. Immune response may be epigenetically regulated in gastric cancer [32]. Here, we evaluated the correlations of risk scores with the infiltration levels of immune cells. High risk scores were characterized by increased infiltration levels of B cells naïve, T cells CD4 memory resting, monocytes, and macrophages M2, while low risk scores had the characteristics of increased infiltration levels of T cells CD8, T cells CD4 memory activated, T cells follicular helper, and macrophages M1 in gastric cancer tissues. Furthermore, high-risk samples displayed elevated stromal scores. Mao et al. have demonstrated that stromal scores may be a prognostic index for gastric cancer, which are in relation to tumor immune microenvironment [33]. More importantly, we found that the risk score was in relation to overall responses to the anti-PD-L1 immunotherapy. Samples with progressive disease exhibited the highest risk score, and those with complete response had the lowest risk score. These data suggested that gastric cancer patients with low risk score exhibited high response to anti-PD-L1 immunotherapy. Hence, compared with previous gene models, this signature could be utilized for predicting the response to immunotherapy [34, 35].

Despite this, there are some disadvantages in our study. First of all, although the immune-based gene signature exhibited the well performance in predicting gastric cancer prognosis in different datasets, we will further validate the predictive efficacy of this signature in prospective cohorts. Secondly, more experiments should be carried out for investigating the therapeutic effects of the small molecular agents against gastric cancer. Thirdly, the interactions of this signature with tumor microenvironment will be further validated in the coculture system.

## 5. Conclusion

This study proposed and externally verified the reproducible immune-based gene signature for predicting risk stratification as well as immunotherapeutic efficacy of gastric cancer, which might assist oncologists make personalized immunotherapy scheme for each subject.

## Figures and Tables

**Table 1:** Prognosis-related immune-related genes in gastric cancer.

ID	HR	95% CI lowest	95% CI highest	*p* value
INHBA	1.243952	1.045644	1.479870	0.013751
F2R	1.234062	1.044292	1.458318	0.013561
PGF	1.178581	1.00036	1.388554	0.049499
PDGFRB	1.221756	1.033238	1.444669	0.019162
FABP4	1.201410	1.045077	1.381128	0.009884
GHR	1.249665	1.079721	1.446358	0.002804
STC1	1.318622	1.117481	1.555969	0.001055
NRP1	1.377816	1.159051	1.637872	0.00028
CTLA4	0.835463	0.705530	0.989325	0.037123
SLC22A17	1.237162	1.060073	1.443834	0.006932
AGT	1.208708	1.018537	1.434385	0.029987
GCG	1.176646	1.040025	1.331215	0.009790
CARD11	1.185447	1.004931	1.398390	0.043558
RNASE2	1.236653	1.042710	1.466669	0.014667
PENK	1.148276	1.003847	1.313485	0.043806
CXCR4	1.280472	1.085135	1.510972	0.003418
AGTR1	1.149173	1.000137	1.320418	0.049775
S100A12	1.171564	1.006071	1.364281	0.041564
PROC	1.211598	1.036397	1.416415	0.016014
OGN	1.173089	1.004497	1.369978	0.043732
DKK1	1.175657	1.006877	1.372728	0.040693
RBP4	1.212545	1.036249	1.418834	0.016210
APOD	1.333790	1.127181	1.578271	0.000796

Abbreviations: HR: hazard ratio; CI: confidence interval.

**Table 2:** Clinical characteristics of patients in the high- and low-risk groups from the training set.

Characteristics		High risk (*N* = 175)	Low risk (*N* = 175)	Total (*N* = 350)	*p* value
Age	<65	78	72	150	0.5892
	≥65	97	103	200	
Stage	Stage I	16	33	49	0.0106
	Stage II	59	52	111	
	Stage III	76	79	155	
	Stage IV	24	11	35	
T	T1	3	13	16	0.0272
	T2	43	31	74	
	T3	75	86	161	
	T4	50	45	95	
	Tx	4	0	4	
M	M0	150	162	312	0.1705
	M1	15	8	23	
	Mx	10	5	15	
N	N0	41	63	104	0.0884
	N1	50	43	93	
	N2	38	34	72	
	N3	40	31	71	
	Nx	6	4	10	
Gender	Female	60	64	124	0.7374
	Male	115	111	226	
Grade	G1	5	4	9	0.7498
	G2	59	66	125	
	G3	106	101	207	
	Gx	5	4	9	

**Table 3:** Clinical characteristics of patients in the high- and low-risk groups from the GSE66229 dataset.

Characteristics		High risk (*N* = 150)	Low risk (*N* = 150)	Total (*N* = 300)	*p* value
Age	<65	87	74	161	0.1647
	≥65	63	76	139	
Stage	Stage I	9	21	30	0.0073
	Stage II	40	56	96	
	Stage III	55	40	95	
	Stage IV	45	32	77	
	NA	1	1	2	
T	T2	75	111	186	<0.0001
	T3	60	31	91	
	T4	14	7	21	
	NA	1	1	2	
M	M0	131	142	273	0.0437
	M1	19	8	27	
N	N0	14	24	38	0.1309
	N1	62	69	131	
	N2	47	33	80	
	N3	27	24	51	
Gender	Female	53	48	101	0.6251
	Male	97	102	199	

**Table 4:** Prediction of potential small molecular components based on the risk scores.

Rank	CMap name	Mean	*N*	Enrichment	*p*	Specificity	Percent nonnull
1	Lomustine	-0.804	4	-0.949	<0.0001	0	100
2	Oxybenzone	-0.454	4	-0.851	0.00093	0.0141	75
3	Trifluridine	0.291	4	0.819	0.00187	0.0421	50
4	Diethylstilbestrol	-0.493	6	-0.693	0.00209	0.0082	66
5	Prestwick-642	-0.336	4	-0.814	0.00223	0.0276	50
6	Chlorhexidine	-0.514	5	-0.71	0.00445	0.015	80
7	Indoprofen	-0.351	4	-0.765	0.00627	0.0333	50
8	Prestwick-857	-0.366	4	-0.762	0.00656	0.0127	50
9	Bromperidol	0.463	3	0.838	0.00831	0	66
10	Chenodeoxycholic acid	-0.381	4	-0.712	0.014	0.0923	50
11	STOCK1N-35874	-0.566	2	-0.915	0.01467	0.0331	100
12	Perhexiline	0.487	4	0.693	0.01862	0.1088	75
13	Pseudopelletierine	0.365	4	0.69	0.01908	0.0184	50
14	Hydrochlorothiazide	-0.446	5	-0.622	0.02005	0.0229	60
15	PHA-00767505E	-0.404	4	-0.687	0.02071	0.0127	75
16	Mometasone	-0.484	4	-0.68	0.02312	0.0342	75
17	Ciclopirox	0.39	4	0.679	0.02316	0.1594	75
18	0173570-0000	0.425	6	0.569	0.02384	0.1429	66
19	Eticlopride	-0.348	4	-0.676	0.02463	0.0758	50
20	Puromycin	0.441	4	0.663	0.02906	0.3258	75
21	Calcium pantothenate	0.379	4	0.662	0.02962	0.0413	50
22	Pimozide	0.426	4	0.659	0.03093	0.1457	75
23	Clopamide	-0.46	4	-0.657	0.03191	0.0284	75
24	Flumequine	0.476	4	0.647	0.03686	0.0567	75
25	Harpagoside	-0.178	4	-0.642	0.03945	0.0764	50
26	5194442	0.231	4	0.632	0.04559	0.0903	75

## Data Availability

The data used to support the findings of this study are included within the supplementary information files.

## Abbreviations

OS: Overall survival
TCGA: The Cancer Genome Atlas
GEO: Gene Expression Omnibus
IRGs: Immune-related genes
FC: Fold change
GO: Gene Ontology
KEGG: Kyoto Encyclopedia of Genes and Genomes
BP: Biological process
CC: Cellular component
MF: Molecular function
LASSO: Least absolute shrinkage and selector operation
ROC: Receiver operating characteristic
HR: Hazard ratio
CI: Confidence interval
GSEA: Gene set enrichment analysis
GDSC: Genomics of Drug Sensitivity in Cancer
CMap: Connectivity map
ESTIMATE: Estimation of stromal and immune cells in malignant tumors using expression data
AUCs: Area under the curves.

## Supplementary Materials

Supplementary 1 Supplementary Table 1: 1001 overlapped immune-related genes in TCGA, GSE66229, and Imvigor210 datasets.

Supplementary 2 Supplementary Table 2: differentially expressed immune-related genes in gastric cancer from TCGA dataset.

Supplementary 3 Supplementary Table 3: differentially expressed genes between high- and low-risk gastric cancer samples.
