# Supplementary material for: Development and Verification of an Immune-Based Gene Signature for Risk Stratification and Immunotherapeutic Efficacy Assessment in Gastric Cancer
Source: Dis Markers. 2021 Nov 11;2021:4251763. doi: 10.1155/2021/4251763 (PMC8602949; doi:10.1155/2021/4251763)
Supplement: Supplementary 1 — Supplementary Table 1: 1001 overlapped immune-related genes in TCGA, GSE66229, and Imvigor210 datasets. [file 4251763.f1.pdf]

Supplementary table 1. 1001 overlapped immune-related genes in TCGA, GSE66229 and Imvigor210 datasets.

AZGP1
B2M
CALR
CANX
CD1A
CD1C
CD1D
CD1E
CD4
CD8A
CD8B
CD74
CREB1
CTSB
CTSE
CTSS
FCER1G
FCGRT
PDIA3
HFE
HLA-A
HLA-B
HLA-C
HLA-DMA
HLA-DMB
HLA-DOA
HLA-DOB
HLA-DPA1
HLA-DPB1
HLA-DQA1
HLA-DQB1
HLA-DRA
HLA-DRB1
HLA-E
HLA-F
HLA-G
MR1
HSPA1A
HSPA1L
HSPA2
HSPA4
HSPA5
HSPA6
HSPA8
HSP90AA1
HSP90AB1
ICAM1
IFNG
KIR2DL4
KLRC1

KLRD1
LTA
CIITA
MICA
MICB
NFYA
NFYB
NFYC
LGMN
PSMB8
PSMC1
PSMC2
PSMC3
PSMC4
PSMC5
PSMC6
PSMD1
PSMD2
PSMD3
PSMD4
PSMD5
PSMD7
PSMD8
PSMD10
PSMD11
PSMD13
PSME1
RELB
RFX5
RFXAP
TAP1
TAP2
TAPBP
THBS1
KLRC4
AP3B1
RFXANK
PSMD6
PSME3
PSMD14
IFI30
PROCR
ADRM1
TRPC4AP
CD209
ERAP1
TAPBPL
ERAP2
ULBP3
ULBP1
RAET1E
UBR1
PDIA2

HAMP
PI3
CAMP
PPBP
REG3G
CXCL14
CXCL16
SLPI
CXCL10
CXCL9
CXCL5
CXCL11
CXCL6
CXCL1
CXCL12
CXCL13
CXCL2
PF4
XCL1
CXCL3
CCL13
DEFB1
CCL8
TMSB10
DEFA6
DEFA5
DEFA4
LCN2
LCN1
BPI
S100A9
S100A8
S100A12
CCR10
PENK
MMP12
LEAP2
SFTPD
PTGDS
TMSB4X
PGLYRP1
ZC3HAV1
S100B
S100A13
S100A6
SERPIND1
S100P
S100A7
PGLYRP3
S100A10
S100A2
LCN12
PGLYRP4

S100A11
S100A5
S100A3
S100A1
LMBR1L
S100A7A
COLEC12
TMSB4Y
S100A14
S100A16
ZC3HAV1L
IFNAR1
AZU1
S100G
TINAGL1
IFNGR1
SLC22A17
WFIKKN1
WFDC2
IL6
TGFB1
PF4V1
MMP9
TLR4
A2M
NFKB1
APOBEC3G
FABP6
NOD2
RBP1
TLR2
SLC40A1
PLAU
IL1B
PAEP
MUC5AC
PLTP
MX1
DDX58
IRF3
LBP
RBP4
NOX4
LTF
RBP5
FABP5
FABP3
FABP2
FABP4
R3HDML
OASL
CRABP2
CRABP1

RBP7
DUOX1
OBP2B
RBP2
CETP
C8G
PI15
NOX1
APOD
ORM1
TNF
CTSG
PRTN3
MAPK1
PML
CYBB
ISG20
BCL3
ISG20L2
DUOX2
TLR3
TFRC
IFIH1
LRP1
TRIM5
GDF15
NEDD4
ADIPOQ
STAT3
STAT1
SOCS3
SEMG1
TNFSF10
CCL20
SOCS1
RNASEL
IRF1
IL15
APOBEC3F
CD40
TLR7
PPIA
ZYX
NLRX1
PGC
VEGFA
IKBKE
ISG15
DHX58
TNFAIP3
TFR2
MUC4
F2R

ELN
MAPT
LYZ
CCL5
CYLD
CST4
CSRP1
MAPK14
JUN
ITGAV
IRF5
TLR8
GNLY
CD81
EIF2AK2
APOM
CACYBP
NOD1
MAPK8
MAPK3
BST2
BPHL
PLA2G2A
GRN
PDGFRA
GNAI1
WNT5A
FURIN
ADAR
TYK2
TRAF3
TPM2
NEO1
AHNAK
TLR1
TK2
PRDX2
MX2
FGF2
FGA
TCF7L2
F2RL1
MSR1
NFKBIZ
LMBR1
SRC
MPO
ELAVL1
ROBO3
SP1
SOD1
DLL4
ECD

SLC11A1
DMBT1
SKIV2L
SEMG2
DES
DCK
DAXX
TNFRSF10A
TNFRSF10B
EED
CCL4
LIMS1
TMPRSS6
SPINK5
MARCO
BECN1
TNFSF11
CSK
KCNH2
JUND
JAK1
CLDN4
CCL28
RNASE3
IRF7
IREB2
ILK
IL18
LTB4R
MASP2
TRIM27
RELA
IL7R
IL1A
PTX3
IFNAR2
SYTL1
APOBEC3C
DDX17
PTGS2
CD40LG
CD14
MASP1
PROC
MAP2K2
MAP2K1
NDRG1
IRF9
TRIM22
LANCL1
HMOX1
HMGB1
RNASE7

ABCC4
HGF
HDAC1
PLSCR1
BACH2
TANK
PIK3CG
ARRB1
RSAD2
TBK1
PDGFRB
PDCD1
PCSK2
PCSK1
ARG2
AQP9
FASLG
APOH
BIRC5
ANXA6
VIM
VCAM1
PRDX1
GBP2
ALB
SLC29A3
OAS1
AGER
UNC93B1
TNFSF4
ACTA1
ACO1
SERPINA3
CCL15
CCL16
CCL19
CCL18
CCL17
CCL26
CCL22
CCR3
CCR7
CCR8
CCL2
CCL21
CCL7
CCL3
CCL11
CCR5
CCL23
CCL25
CCR1
CCL24

CXCR4
CXCR6
CCR4
PTK2B
CDH1
LTBP1
IL10
PPARG
FGR
MIF
CRP
JAK2
PTK2
PTGDR
CD86
HCK
VDR
OLR1
TXK
RNASE2
CD79A
CD79B
LYN
SYK
BTK
BLNK
VAV3
VAV1
VAV2
RAC1
RAC2
RAC3
PPP3CA
PPP3CB
PPP3CC
PPP3R1
CHP2
NFAT5
NFATC1
NFATC2
NFATC3
NFATC4
HRAS
KRAS
NRAS
FOS
CARD11
BCL10
MALT1
CHUK
IKBKB
IKBKG
NFKBIA

NFKBIB
NFKBIE
CD19
CR2
PIK3R5
PIK3R1
PIK3R2
PIK3R3
PIK3CA
PIK3CB
PIK3CD
AKT3
AKT1
AKT2
GSK3B
INPP5D
CD22
CD72
PTPN6
FCGR2B
RASGRP3
PLCG2
IFITM1
C3
C5
CKLF
CMA1
CX3CL1
CXCL17
EDN1
EDN2
EDN3
FGF10
PROK2
SAA1
SBDS
SEMA3A
SEMA3B
SEMA3C
SEMA3D
SEMA3E
SEMA3F
SEMA3G
SEMA4A
SEMA4B
SEMA4C
SEMA4D
SEMA4F
SEMA4G
SEMA5A
SEMA5B
SEMA6A
SEMA6B

SEMA6C
SEMA6D
SEMA7A
SLIT1
SLIT2
TNC
C5AR1
CMKLR1
CX3CR1
CXCR3
CYSLTR1
CYSLTR2
EDNRA
EDNRB
FPR1
FPR2
GPR17
LTB4R2
PLAUR
PLXNA1
PLXNA2
PLXNA3
PLXNB1
PLXNB2
PLXNB3
PLXNC1
PLXND1
PTAFR
ROBO1
ROBO2
XCR1
ADM
ADM2
AGT
AMH
ANGPTL7
APLN
AREG
ARTN
BDNF
BMP1
BMP2
BMP3
BMP4
BMP5
BMP6
BMP7
BMP8A
BMP8B
BTC
CALCA
CAT
CCK

CD320
CD70
CGA
CHGA
CHGB
CLCF1
CLEC11A
CMTM1
CMTM2
CMTM3
CMTM4
CMTM6
CMTM7
CMTM8
CNTF
CSF1
CSF2
CSF3
CSPG5
CTF1
DKK1
EBI3
EREG
ESM1
FAM3B
FAM3C
FAM3D
FGF1
FGF12
FGF13
FGF18
FGF19
FGF20
FGF3
FGF7
FGF9
FLT3LG
GAL
GAST
GCG
GDF10
GDF11
GDF9
GDNF
GHRH
GHRL
GIP
GKN1
GMFB
GMFG
GNRH1
GNRH2
GPI

GREM1
GREM2
GRP
GUCA2A
HBEGF
HDGF
IGF1
IGF2
IL11
IL12A
IL16
IL17B
IL17C
IL17D
IL19
IL1RN
IL23A
IL24
IL32
IL33
IL6ST
IL7
INHA
INHBA
INHBB
INSL3
INSL6
JAG1
JAG2
KITLG
KL
LEFTY1
LIF
LRSAM1
LTB
LTBP3
LTBP4
MDK
MIA
MLN
NAMPT
NDP
NENF
NGF
NMB
NPPC
NPY
NRG1
NRG2
NRG4
NRTN
NTF3
NTS

NUDT6
OGN
OSGIN1
OSM
OXT
PDGFA
PDGFB
PDGFC
PDGFD
PDGFRL
PGF
PNOC
POMC
PPY
PSPN
PTHLH
PTN
PYY
RABEP1
RABEP2
REG1A
RETN
RETNLB
SCG2
SCGB3A1
SCT
SECTM1
SLURP1
SPP1
SST
STC1
STC2
TAC1
TDGF1
TGFA
TGFB2
TGFB3
THPO
TNFRSF11B
TNFSF12
TNFSF13
TNFSF13B
TNFSF14
TNFSF15
TNFSF18
TNFSF8
TNFSF9
TOR2A
TSLP
TXLNA
UCN
UCN2
UCN3

UTS2
VEGFB
VEGFC
VGF
VIP
ACVR1B
ACVR1C
ACVR2A
ACVR2B
ACVRL1
ADCYAP1R1
ADIPOR1
ADIPOR2
ADRB1
ADRB2
AGTR1
AMHR2
ANGPT1
ANGPTL1
ANGPTL2
ANGPTL3
ANGPTL4
ANGPTL6
AR
AVPR1A
AVPR2
BMPR1A
BMPR1B
BMPR2
BRD8
C3AR1
CALCRL
CNTFR
CRIM1
CRLF1
CRLF2
CRLF3
CSF1R
CSF2RA
CSF2RB
CSF3R
EGFR
ENG
EPOR
ESR1
ESR2
ESRRA
ESRRG
FGFR1
FGFR2
FGFR3
FGFR4
FGFRL1

FLT1
FLT3
FLT4
GALR2
GHR
GIPR
GLP1R
GLP2R
HNF4A
HNF4G
HTR3A
IFNGR2
IGF1R
IGF2R
IL10RA
IL10RB
IL11RA
IL12RB1
IL12RB2
IL13RA1
IL13RA2
IL15RA
IL17RA
IL17RB
IL17RC
IL17RD
IL17RE
IL18R1
IL18RAP
IL1R1
IL1R2
IL1RAP
IL1RL1
IL1RL2
IL20RA
IL20RB
IL21R
IL22RA1
IL27RA
IL2RA
IL2RB
IL2RG
IL31RA
IL3RA
IL4R
IL6R
IL9R
INSR
KDR
LEPR
LGR4
LGR5
LGR6

LIFR
LTBR
MC1R
MET
MLNR
MPL
MTNR1A
NGFR
NPR1
NPR3
NR0B2
NR1D1
NR1D2
NR1H2
NR1H3
NR1H4
NR1I2
NR1I3
NR2C1
NR2C2
NR2F1
NR2F2
NR2F6
NR3C1
NR3C2
NR4A1
NR4A2
NR4A3
NR5A2
NR6A1
NRP1
NRP2
OGFR
OPRL1
OSMR
OXTR
PGR
PGRMC2
PPARA
PPARD
PRLR
PTGER1
PTGER2
PTGER3
PTGER4
PTGFR
PTH2R
RARA
RARB
RARG
RORA
RORC
RXRA

RXRB
RXRG
SCTR
SDC1
SDC2
SDC3
SDC4
SORT1
SSTR1
SSTR2
SSTR5
TACR1
TEK
TGFBR1
TGFBR2
TGFBR3
THRA
THRB
TIE1
TNFRSF10C
TNFRSF10D
TNFRSF11A
TNFRSF12A
TNFRSF13B
TNFRSF13C
TNFRSF14
TNFRSF17
TNFRSF18
TNFRSF19
TNFRSF1A
TNFRSF1B
TNFRSF21
TNFRSF25
TNFRSF4
TNFRSF8
TNFRSF9
TUBB3
VIPR1
VIPR2
PTPN11
ICAM2
ITGAL
ITGB2
PAK1
TYROBP
LCK
FCGR3A
FCGR3B
NCR1
NCR3
CD247
ZAP70
LCP2

PLCG1
SH3BP2
FYN
SHC2
SHC4
SHC3
SHC1
GRB2
SOS1
SOS2
ARAF
BRAF
RAF1
HCST
CD48
CD244
PRKCA
PRKCG
SH2D1B
SH2D1A
FAS
GZMB
PRF1
CASP3
BID
CD3D
CD3E
CD3G
PTPRC
ITK
TEC
NCK1
NCK2
GRAP2
PAK2
PAK3
PAK4
RHOA
CDC42
CD28
ICOS
MAP3K8
MAP3K14
CTLA4
CBLC
CBL
CBLB
CDK4
RASGRP1
PDK1
PRKCQ
