# Supplementary material for: Development and Verification of an Immune-Based Gene Signature for Risk Stratification and Immunotherapeutic Efficacy Assessment in Gastric Cancer
Source: Dis Markers. 2021 Nov 11;2021:4251763. doi: 10.1155/2021/4251763 (PMC8602949; doi:10.1155/2021/4251763)
Supplement: Supplementary 2 — Supplementary Table 2: differentially expressed immune-related genes in gastric cancer from TCGA dataset. [file 4251763.f2.pdf]

Supplementary table 2. Differentially expressed immune-related genes in gastric cancer from TCGA dataset.

ID	logFC	AveExpr	t	P.Value
GKN1	-5.600673996	4.009008516	-7.432419052	6.30E-13
DES	-3.505058771	6.547346183	-5.726476932	1.99E-08
SST	-3.103550283	2.274941101	-7.168328108	3.58E-12
CHGA	-3.0417453	2.60690724	-6.509759938	2.21E-10
DEFA5	-2.871228105	1.425728883	-6.213112301	1.28E-09
RBP2	-2.421188816	2.257667791	-5.317107969	1.74E-07
PGC	-2.203205743	7.169707894	-2.797649082	0.005390955
FABP4	-2.170654628	2.323219205	-7.089240447	5.96E-12
DEFA6	-2.166843534	1.321899253	-5.083035078	5.66E-07
CXCL17	-2.136153403	4.802137287	-3.905350941	0.000110055
GIP	-2.018670901	0.723804257	-9.405243549	3.78E-19
ANGPTL1	-2.002785883	1.810724762	-6.927426629	1.67E-11
CCL21	-1.905972009	5.456172996	-4.962043476	1.03E-06
VIP	-1.835108363	1.337808301	-5.9880636	4.65E-09
ESRRG	-1.78222298	0.921569583	-10.27738331	3.42E-22
CNTFR	-1.728676507	1.573454771	-6.215276278	1.26E-09
GHRL	-1.695787041	1.846499313	-5.88872384	8.13E-09
PTN	-1.674534367	3.164446254	-7.22490535	2.47E-12
OGN	-1.632634752	3.196337471	-3.994860353	7.68E-05
S100B	-1.614943451	2.651992723	-7.505428379	3.87E-13
S100A8	-1.596109273	4.606517398	-3.698218578	0.000246763
LTF	-1.506145565	3.810054486	-3.022972556	0.002660667
PENK	-1.434532241	1.150572117	-5.254741535	2.39E-07
SLURP1	-1.375316869	0.650359626	-4.326566643	1.91E-05
FABP2	-1.351996178	1.518307663	-3.922863203	0.000102622
S100A12	-1.333802964	1.944882309	-4.831626079	1.92E-06
NPPC	-1.318046359	0.691432059	-8.69109311	8.77E-17
SLC22A17	-1.309267644	3.006891877	-5.753803505	1.71E-08
S100G	-1.301184948	0.752862778	-6.551271621	1.72E-10
FAM3B	-1.300854446	3.522594171	-3.556844354	0.000419309
NPY	-1.284607894	0.956931344	-5.760277447	1.65E-08
TPM2	-1.28067254	7.14913488	-4.161353405	3.86E-05
CHGB	-1.255060716	1.422450761	-3.892408821	0.000115873
ADRB2	-1.242723216	1.117085028	-9.409708801	3.65E-19
PCSK2	-1.238811544	0.478911981	-8.491863517	3.81E-16
CTSG	-1.232932669	1.08025588	-6.253968397	1.01E-09
PTGDS	-1.228801065	5.755978073	-3.757721206	0.000196409
HSPA2	-1.227758452	2.606685206	-5.796377948	1.36E-08
GHR	-1.212710399	1.396894007	-6.789754594	3.97E-11
GREM2	-1.211520737	1.824702744	-4.632223519	4.87E-06
GCG	-1.170389432	0.49716454	-5.481207546	7.41E-08
CMA1	-1.158598638	0.672675325	-7.562561885	2.63E-13
CSRP1	-1.14050923	6.121975544	-5.069759139	6.05E-07
CXCL12	-1.13799746	4.289708005	-4.553664123	6.97E-06
AGTR1	-1.104996844	1.086639514	-4.874393998	1.56E-06
EDN3	-1.072329103	1.884552631	-3.892834027	0.000115678
TNFRSF17	-1.071215952	2.00667008	-4.312243439	2.03E-05
APOD	-1.045348852	5.98067481	-2.412757657	0.016271651
ANGPTL7	-1.043813448	0.696992094	-6.874286296	2.34E-11

RXRG	-1.030542678	0.420134674	-10.58711967	2.61E-23
TUBB3	1.001433015	1.393675715	6.262883005	9.58E-10
FGFRL1	1.003076969	4.82749629	4.850613081	1.75E-06
AQP9	1.003080849	1.954023052	4.250589945	2.64E-05
NRP1	1.003190551	4.710082449	6.056711748	3.15E-09
TAPBP	1.003377182	7.798934192	7.905150842	2.51E-14
C3AR1	1.006011646	3.702610038	4.61865905	5.18E-06
ULBP3	1.008521072	2.114941597	5.365616125	1.35E-07
LTB	1.014249444	3.993751457	3.894297246	0.000115007
PSME3	1.014263968	6.61213738	10.28748187	3.15E-22
SRC	1.0143323	6.150180371	7.093839029	5.79E-12
EIF2AK2	1.017724739	4.6759311	8.119777654	5.56E-15
EDN1	1.022228544	3.904157275	4.856647496	1.70E-06
HLA-F	1.027820015	6.630450231	5.347007815	1.49E-07
CCR1	1.031241821	3.056560124	5.34597214	1.50E-07
RNASE2	1.036459999	1.724214295	5.334984677	1.59E-07
PTK2	1.036552756	5.630557966	9.705273638	3.54E-20
CXCL2	1.049359804	4.909463376	3.653049944	0.000292853
ITGB2	1.049928665	4.561917125	4.405419487	1.35E-05
PIK3CB	1.050960109	4.380720418	8.690030585	8.84E-17
TMSB10	1.055632604	11.64962343	7.933626989	2.06E-14
NR5A2	1.057593886	2.469645745	4.78553653	2.39E-06
TINAGL1	1.057620338	5.65382835	6.636818424	1.02E-10
IL27RA	1.060012032	3.550768603	6.550675617	1.73E-10
CANX	1.06008905	8.215424261	9.792173824	1.77E-20
PSMD1	1.066365495	6.631339197	11.14510668	2.27E-25
TNFRSF4	1.067356039	2.659514059	6.832571844	3.04E-11
NFYA	1.06756672	4.282743791	8.537102182	2.73E-16
GMFB	1.075486128	4.228037441	9.589544456	8.87E-20
CMTM1	1.076654104	2.139743304	9.066490021	5.18E-18
CCL4	1.077118072	3.639242289	4.591275473	5.87E-06
CD86	1.07949623	3.019476809	5.770986899	1.56E-08
PROC	1.084044638	1.929361883	4.683644373	3.84E-06
LCK	1.088457845	3.773605755	4.664755405	4.19E-06
PTPN6	1.09066661	5.319877092	8.165335977	4.02E-15
HSPA5	1.097706096	8.382301278	9.894971415	7.74E-21
MIF	1.097741513	7.302654939	6.896596533	2.03E-11
IRF7	1.098569669	5.117893278	6.438178214	3.40E-10
CYBB	1.099288127	4.448636829	4.699294737	3.57E-06
TNFSF13B	1.1044931	3.557799131	5.729195305	1.96E-08
GZMB	1.107886017	3.900544075	3.993634757	7.72E-05
CTLA4	1.109099123	1.954266179	5.919680232	6.84E-09
OSM	1.113332142	1.862025289	5.016511138	7.86E-07
NMB	1.114015131	3.764554936	6.251828663	1.02E-09
IL24	1.115605189	2.093794336	4.762426435	2.66E-06
LCP2	1.115938667	3.893428427	6.074165676	2.85E-09
CST4	1.119604039	1.072673486	5.182123942	3.45E-07
PSMD3	1.119858119	6.670788088	7.155843007	3.88E-12
NR2C1	1.12501238	3.674038237	10.73069287	7.80E-24
PSMD14	1.126665497	4.518408346	12.12893644	3.94E-29
WNT5A	1.133099201	3.379308559	5.582502061	4.33E-08
CMTM6	1.136177961	5.721569839	11.14370589	2.30E-25
PSMD11	1.145635356	6.034842337	11.56911177	5.69E-27

NRAS	1.146329043	5.109718608	9.392777144	4.17E-19
IL23A	1.146808056	2.096506192	6.274195058	8.97E-10
RBP4	1.146922257	3.392805958	2.846755687	0.00463931
HDGF	1.15116845	8.290769467	9.724273059	3.04E-20
CACYBP	1.152931457	5.662607604	10.2323122	4.96E-22
TLR2	1.166392778	2.595914641	7.177354842	3.37E-12
S100A10	1.167152018	9.567618942	5.984738225	4.74E-09
STC2	1.172213359	1.955540803	6.736637873	5.52E-11
IL2RA	1.17865725	2.275278173	6.327579395	6.55E-10
F2RL1	1.182512046	5.378683397	4.826901941	1.96E-06
PLSCR1	1.182666929	6.385605839	8.868357022	2.33E-17
PDIA3	1.185356419	8.326016103	10.49439259	5.66E-23
SAA1	1.186883284	4.36615114	2.496422252	0.012938191
CXCR4	1.188869198	5.954287207	5.141895928	4.23E-07
TAP2	1.19424062	3.947160844	7.958487721	1.73E-14
PDGFB	1.198322221	3.868276565	7.798208084	5.28E-14
ZC3HAV1L	1.200624217	3.382709436	7.501788776	3.96E-13
ADAR	1.205522976	6.923634629	10.10437331	1.42E-21
CTSB	1.221968808	9.041614609	7.789209766	5.61E-14
BST2	1.225818713	7.444269196	4.971362079	9.80E-07
PGF	1.239156825	2.716101305	8.230187518	2.53E-15
JAG2	1.241710444	3.326060367	7.707937487	9.81E-14
VEGFA	1.24410416	4.683477065	6.958031812	1.38E-11
DLL4	1.250868981	3.450574602	8.538080227	2.72E-16
PLXNA3	1.252104571	3.660077596	8.386935989	8.18E-16
PLXND1	1.260782637	5.135883061	7.110053474	5.21E-12
SLC11A1	1.261622855	2.14713687	7.343340497	1.14E-12
IKBKE	1.268145162	3.881763926	8.919467759	1.58E-17
LEFTY1	1.277612537	1.49330753	3.411058594	0.000711559
CXCL13	1.278314277	4.377472426	3.299192579	0.001054597
TNFSF15	1.284728819	2.714024774	7.367671823	9.68E-13
CTSS	1.299765934	6.363734476	6.918740029	1.77E-11
DMBT1	1.308773982	4.987554681	2.352511184	0.019120443
CKLF	1.309119148	4.851661634	9.467933091	2.31E-19
TNFSF11	1.31194163	1.551494464	8.300357216	1.53E-15
TNFRSF25	1.315413086	2.855190587	7.284929237	1.67E-12
TNFSF9	1.316543041	2.589210246	4.70704509	3.45E-06
LCN2	1.318747314	9.728898369	2.67131782	0.007857222
PRKCG	1.318856851	1.515805531	5.507174176	6.46E-08
STC1	1.321391173	4.19074756	6.592085899	1.34E-10
NFKBIE	1.34926515	4.199180085	9.61742782	7.12E-20
IL2RG	1.349606444	6.379339776	5.043173581	6.90E-07
CMTM8	1.354388067	4.792444965	9.238783652	1.38E-18
ICAM1	1.377048003	5.324830561	6.470000831	2.81E-10
LYZ	1.382217409	9.856051822	3.427500976	0.000670969
UNC93B1	1.387037966	6.209974927	8.776571161	4.64E-17
HSP90AB1	1.392299926	9.89123999	11.23137257	1.08E-25
FCER1G	1.392352351	7.448246805	6.458327562	3.01E-10
FABP6	1.396010824	2.245290362	5.018467865	7.79E-07
TNFRSF10A	1.406930087	3.828344649	9.717423257	3.21E-20
TAP1	1.426444207	6.895918479	6.972161381	1.26E-11
CARD11	1.441748156	3.376915459	5.408132865	1.09E-07
IFITM1	1.449222964	7.953276893	5.96681088	5.25E-09

DKK1	1.454605799	2.621650456	3.292069181	0.001080959
CMTM7	1.466754732	4.680904877	8.37010173	9.24E-16
SEMA4G	1.47230693	4.665729584	6.862234402	2.52E-11
AMH	1.487734986	1.87337981	5.941276161	6.06E-09
OSMR	1.490040965	4.788375612	7.251485879	2.08E-12
HSP90AA1	1.500561116	9.252712271	12.57251921	7.11E-31
NOX1	1.504998397	1.78202544	4.887972317	1.47E-06
LYN	1.506611721	4.979317468	10.07327531	1.82E-21
CDH1	1.5067699	7.132657965	5.720155471	2.06E-08
PLXNA1	1.511132377	4.567150181	9.959007734	4.61E-21
CCL3	1.513923342	2.383993753	6.982552312	1.18E-11
CBLC	1.517866893	5.860464536	7.199047404	2.93E-12
BMP8A	1.522113644	2.267672299	8.618194596	1.51E-16
MICB	1.526731799	3.053444588	8.361340724	9.85E-16
ISG15	1.549719025	7.008140357	6.546488099	1.77E-10
BID	1.575749802	4.873911213	13.38361753	3.95E-34
IL11	1.580605737	1.788794518	5.894563072	7.87E-09
BMP1	1.592866018	4.498428231	11.2922248	6.37E-26
CCL15	1.605418075	2.852764339	5.859592423	9.56E-09
TFRC	1.606319629	6.200901029	8.057746066	8.63E-15
VAV2	1.610984993	4.742572152	11.0460233	5.33E-25
CXCL6	1.658417264	2.193241842	5.594841761	4.05E-08
STAT1	1.687679006	7.12146997	9.314490928	7.67E-19
APLN	1.721592306	2.30750757	7.593214439	2.14E-13
PDGFRB	1.725331353	5.735592941	8.126935893	5.29E-15
PLAUR	1.729771509	6.361942567	8.539063818	2.70E-16
IL32	1.731357779	7.464222364	7.709396364	9.71E-14
CXCL16	1.73328633	7.249714057	10.47720764	6.54E-23
HNF4G	1.745613962	4.188483354	7.077312101	6.44E-12
TNFRSF12A	1.763406907	5.985473256	8.975559779	1.04E-17
CCL18	1.776948909	4.542316984	4.859390249	1.68E-06
AGT	1.784454591	4.665044932	5.654230963	2.94E-08
TNFRSF10B	1.789205492	5.047465601	12.39083764	3.71E-30
TNFRSF11B	1.8018337	2.617577283	6.317066719	6.97E-10
LGR5	1.817130529	2.665659095	5.715468549	2.11E-08
MDK	1.859154951	8.372193853	8.397598294	7.57E-16
MSR1	1.88296774	3.571637598	8.376037443	8.86E-16
F2R	1.92550785	5.228871154	10.66499104	1.36E-23
IL17RB	2.060179672	3.878850035	10.67912184	1.20E-23
CXCL5	2.066049121	4.531247343	3.857909198	0.000132838
OLR1	2.071198371	2.561235226	9.151277461	2.71E-18
MET	2.114805745	5.037652762	9.691465553	3.95E-20
CXCL11	2.130843287	3.246887123	6.715791503	6.28E-11
PLAU	2.149366078	5.359802835	9.930940207	5.79E-21
LIF	2.1586203	3.475846399	10.44909456	8.26E-23
BIRC5	2.175024354	4.455051719	11.28116896	7.01E-26
CCL20	2.182972542	5.684414803	5.351518906	1.46E-07
CXCL3	2.184308057	4.695682483	6.999581603	1.06E-11
MMP9	2.255760636	4.578903589	7.800458592	5.20E-14
HNF4A	2.286521939	5.180970266	8.761884499	5.18E-17
FCGR3A	2.423048321	4.763223312	9.127127524	3.26E-18
CXCL10	2.43635787	4.880420348	7.158121395	3.82E-12
ESM1	2.460924084	2.542553543	11.8041859	7.15E-28

MMP12	2.5619214	4.540566879	6.489977928	2.49E-10
FGFR4	2.588013934	5.166765408	9.844508283	1.16E-20
CXCL9	2.746989452	5.014336439	7.595007932	2.11E-13
CLDN4	2.788485184	7.215418855	10.2803719	3.34E-22
INHBA	3.023284501	3.898639917	12.56619487	7.53E-31
CXCL1	3.16376669	5.979897119	9.084146046	4.53E-18
GDF15	3.340085013	6.431235537	12.30810696	7.85E-30
SPP1	3.413890902	6.105015229	8.812399689	3.55E-17

adj.P.Val	B
5.09E-12	18.62216115
8.07E-08	8.524264645
2.52E-11	16.92310389
1.17E-09	12.89739302
6.08E-09	11.18657827
6.00E-07	6.428241973
0.008913757	-3.331222848
3.98E-11	16.42353639
1.85E-06	5.290534026
0.000245057	0.267731359
7.89E-18	32.69659246
1.05E-10	15.41493787
3.19E-06	4.720131986
2.12E-08	9.932755974
1.37E-20	39.60316852
6.03E-09	11.19882071
3.54E-08	9.391620732
1.80E-11	17.28311654
0.00017603	0.607181009
3.18E-12	19.10014213
0.000510861	-0.490339539
0.004620431	-2.69084409
8.12E-07	6.12073677
4.85E-05	1.926774326
0.000229014	0.33358295
5.65E-06	4.118944701
1.25E-15	27.33666339
7.12E-08	8.66891098
9.24E-10	13.14197714
0.000840296	-0.985560994
6.93E-08	8.703264866
9.33E-05	1.257447662
0.000256303	0.219240578
7.78E-18	32.7309757
4.89E-15	25.89272487
4.89E-09	11.41831055
0.000417838	-0.276510562
5.78E-08	8.89543248
2.31E-10	14.57129984
1.34E-05	3.227431513
2.73E-07	7.252380429
2.24E-12	19.47664946
1.96E-06	5.227354036
1.85E-05	2.885474627
4.72E-06	4.314524135
0.000256303	0.220831341
5.14E-05	1.867804222
0.025083376	-4.314352632
1.41E-10	15.08771098

1.45E-21	42.14332935
4.66E-09	11.4690399
5.18E-06	4.205583598
6.61E-05	1.616009766
1.46E-08	10.31113323
2.47E-13	21.77888269
1.41E-05	3.168011167
4.79E-07	6.669598338
0.000255514	0.226306711
1.37E-20	39.68529643
3.89E-11	16.45246581
6.06E-14	23.25925547
5.09E-06	4.233182388
5.24E-07	6.576786047
5.25E-07	6.571628658
5.52E-07	6.516967414
8.65E-19	35.02985215
0.00059642	-0.650526891
3.50E-05	2.25460792
1.25E-15	27.32890141
2.07E-13	21.97363154
6.89E-06	3.909896139
5.68E-10	13.6499712
9.24E-10	13.13845662
4.79E-19	35.71421231
1.77E-23	46.8252014
1.80E-10	14.8322401
3.61E-15	26.21855889
2.02E-18	34.12437466
9.11E-17	30.11913426
1.58E-05	3.048533228
6.56E-08	8.760166412
1.07E-05	3.45410724
1.17E-05	3.370578784
4.53E-14	23.57717706
2.28E-19	36.52861953
1.24E-10	15.22485021
1.74E-09	12.47860442
1.00E-05	3.523543815
7.98E-08	8.538627443
0.000176505	0.602485081
3.02E-08	9.559433815
2.49E-06	4.975412057
4.92E-09	11.40614308
7.62E-06	3.805749306
1.34E-08	10.40791048
1.15E-06	5.766680763
2.70E-11	16.84395396
5.21E-22	43.33530647
6.58E-27	55.38033386
1.67E-07	7.771876868
1.77E-23	46.81328519
7.12E-25	50.46782081

8.52E-18	32.60065437
4.38E-09	11.53349779
0.00778658	-3.195716235
7.82E-19	35.1791537
1.91E-20	39.23719934
2.40E-11	16.98039595
2.15E-08	9.914518724
3.18E-10	14.24940826
3.25E-09	11.83898077
5.75E-06	4.097435204
3.70E-16	28.64066368
2.99E-21	41.37832469
0.020099329	-4.112613772
1.40E-06	5.572401917
1.75E-13	22.14407315
4.85E-13	21.05209944
3.23E-12	19.07623039
5.07E-20	38.20354968
5.11E-13	20.99128004
3.06E-06	4.763631721
2.92E-14	24.03193213
8.62E-13	20.44432325
8.74E-11	15.6042998
3.61E-15	26.22561662
1.01E-14	25.1416462
3.53E-11	16.55458654
8.77E-12	18.04379767
2.65E-16	29.01995641
0.001360653	-1.477219038
0.001982563	-1.84130191
7.52E-12	18.201246
1.09E-10	15.3613116
0.028984393	-4.455484606
5.04E-18	33.18026908
1.80E-14	24.52686827
1.26E-11	17.66744754
9.70E-06	3.558007019
0.012698285	-3.669385943
2.43E-07	7.384772047
7.34E-10	13.38367743
1.66E-18	34.34191387
2.20E-06	5.101271601
2.71E-17	31.42235028
1.46E-09	12.66431775
0.001287952	-1.422738164
6.83E-16	27.96322521
9.83E-24	47.56057429
1.55E-09	12.59610728
2.48E-06	4.984628717
8.05E-19	35.12530554
8.04E-11	15.69194462
3.90E-07	6.882707153
2.36E-08	9.816346635

0.002020749	-1.86409661
1.12E-14	25.02175673
1.51E-10	15.01377528
2.69E-08	9.676942474
1.54E-11	17.45300855
2.52E-28	59.34937042
4.44E-06	4.376939047
5.90E-20	37.95347275
8.32E-08	8.490886981
1.44E-19	37.03856897
7.58E-11	15.7564881
2.10E-11	17.11830542
2.07E-15	26.80563787
1.17E-14	24.95943023
9.43E-10	13.11372878
3.95E-31	66.76304228
3.46E-08	9.423218222
7.03E-24	48.08109823
4.15E-08	9.234378146
9.20E-14	22.8284411
3.81E-23	45.9842873
1.58E-07	7.835719911
1.54E-17	32.00005983
1.83E-12	19.67953463
5.82E-14	23.30912336
3.61E-15	26.23271494
8.61E-13	20.4541038
3.27E-21	41.23697009
4.27E-11	16.34856407
1.79E-16	29.43789917
5.06E-06	4.245736648
1.16E-07	8.144674542
9.30E-28	57.71596349
3.44E-09	11.77865561
8.50E-08	8.466160024
9.49E-15	25.2176683
1.08E-14	25.06401004
7.99E-22	42.78871562
7.54E-22	42.90611477
0.000291894	0.090709681
5.12E-17	30.7584439
9.43E-19	34.92145938
3.60E-10	14.12362933
1.76E-19	36.81480838
3.76E-21	41.00601304
7.03E-24	47.98641845
5.14E-07	6.599260218
6.89E-11	15.86242909
4.85E-13	21.06731868
7.52E-16	27.85527284
6.05E-17	30.57594998
2.68E-11	16.85838994
1.02E-25	52.5168721

1.31E-09	12.78128227
3.23E-19	36.12817598
1.83E-12	19.69142462
1.37E-20	39.62746869
2.52E-28	59.29233515
8.10E-17	30.25193898
1.57E-27	56.97570967
5.38E-16	28.2270939
