# Supplementary material for: Development and Verification of an Immune-Based Gene Signature for Risk Stratification and Immunotherapeutic Efficacy Assessment in Gastric Cancer
Source: Dis Markers. 2021 Nov 11;2021:4251763. doi: 10.1155/2021/4251763 (PMC8602949; doi:10.1155/2021/4251763)
Supplement: Supplementary 3 — Supplementary Table 3: differentially expressed genes between high- and low-risk gastric cancer samples. [file 4251763.f3.pdf]

Supplementary table 3. Differentially expressed genes between high- and low-risk gastric cancer samples.

ID	logFC	AveExpr	t	P.Value
VSNL1	-1.047435606	2.74088034	-5.737598266	2.08E-08
ASCL2	-0.953204521	4.169773665	-4.340749116	1.86E-05
IDO1	-0.947197057	3.875146241	-4.216216355	3.17E-05
AC007991.2	-0.916887821	1.585223674	-5.356520093	1.54E-07
MYB	-0.907002167	3.690579861	-6.71733352	7.53E-11
SNORC	-0.775795685	2.433066252	-6.295815667	9.18E-10
CXCL11	-0.756511214	3.404938357	-4.057263345	6.13E-05
PSAT1	-0.751565593	4.972038827	-5.383122466	1.35E-07
MMP12	-0.747358796	4.698922073	-3.250163749	0.001265533
RPL10P6	-0.708773734	4.910755805	-4.575455195	6.61E-06
PBK	-0.702872007	4.310192027	-6.154121286	2.07E-09
SNHG25	-0.698528514	4.814090651	-4.582270887	6.41E-06
CPNE7	-0.696328414	2.409538795	-5.154352405	4.27E-07
GPA33	-0.694450204	3.503869769	-2.94901627	0.003402607
CXCL9	-0.661160824	5.209278196	-3.087747713	0.002178197
KCNQ1	-0.653375352	5.115470652	-4.340218194	1.87E-05
CXCL10	-0.652198042	5.058291333	-3.257206779	0.001235456
CKS2	-0.652098596	7.20773849	-6.951678412	1.78E-11
ATP10B	-0.643638803	3.86224413	-3.283680632	0.00112823
PLEKHS1	-0.627726193	4.205587715	-3.516406678	0.000495119
ADGRG7	-0.626412237	2.341067738	-3.387318765	0.000786255
SNORD104	-0.625527572	5.014326146	-5.324983994	1.81E-07
CDCA7	-0.624847291	5.209722962	-5.136948975	4.65E-07
NOS2	-0.62455855	2.416269237	-2.984209356	0.003043303
PTMAP4	-0.61328363	4.921727581	-5.861391488	1.06E-08
CASP1	-0.612120526	4.782910685	-4.817599408	2.17E-06
BATF2	-0.611445562	3.912265104	-4.566617867	6.88E-06
LRRC26	-0.610843304	3.020156554	-3.681967283	0.000268045
SAPCD2	-0.606896002	4.657015133	-5.186173009	3.64E-07
LINC01871	-0.605355146	3.129680587	-4.608337732	5.70E-06
AMH	-0.594869973	1.999494414	-4.021990741	7.08E-05
KRT8P36	-0.591698124	2.441720193	-3.737668782	0.000216923
GAD1	-0.589977514	1.412158962	-4.769503079	2.72E-06
PPIC	0.580197827	5.64655415	6.58907792	1.63E-10
COX4I2	0.580277481	2.370714828	8.106750745	8.87E-15
ABCA1	0.580944151	3.352282368	6.367642003	6.05E-10
KLF2	0.581009478	6.194896875	4.778309834	2.61E-06
STUM	0.581066171	0.814658642	6.015744175	4.52E-09
SALL4	0.581211116	1.778158752	3.889166791	0.000120415
ZFP36	0.581278487	8.364447947	5.679239196	2.85E-08
NOTCH2	0.581357752	4.66050098	6.605981162	1.47E-10
CYP2B6	0.581362748	2.455814438	2.752931535	0.006215007
ADAMTS7	0.582081194	2.662282388	6.588573344	1.64E-10
RPL39L	0.582149808	3.963767124	4.049465369	6.33E-05
AC005165.1	0.582259538	0.719817317	6.500944744	2.76E-10
NPR2	0.582786923	2.492176276	8.35909681	1.52E-15
TAC1	0.582798451	0.707104495	4.862052533	1.76E-06
PLA1A	0.583077004	2.953546217	4.40410138	1.41E-05
SMYD1	0.583661591	0.806011185	3.713055081	0.000238262

MX2	0.583700283	4.447701844	5.247425525	2.68E-07
ITIH3	0.585228373	0.814242032	8.82235579	5.44E-17
C2CD4B	0.585321797	3.727559495	3.586554498	0.000382834
MSC-AS1	0.585424015	1.510514427	8.805744711	6.15E-17
AL136084.3	0.585519515	2.333227674	4.701699091	3.72E-06
LAMB1	0.58552702	5.904145874	6.488576318	2.97E-10
GAP43	0.585719326	0.904709916	6.627032761	1.30E-10
ITM2C	0.585948691	6.639430419	5.284868714	2.22E-07
GRID1	0.586046025	0.929506082	7.923113894	3.13E-14
AC037198.1	0.58617932	1.116069193	7.427445633	8.56E-13
LINC01082	0.586830353	1.568220176	5.360663456	1.51E-07
SOX17	0.586871526	2.080637089	7.211801477	3.45E-12
NTRK2	0.587158063	1.433631936	4.559851541	7.09E-06
FAM124B	0.587307592	1.209152882	8.62274224	2.32E-16
SNORD114-3	0.587449903	0.796297599	6.660363351	1.06E-10
PLAU	0.588206513	5.53177901	4.676540981	4.18E-06
CORIN	0.588607925	1.115227146	7.487356494	5.79E-13
SNTA1	0.588961833	3.620993942	5.517066764	6.72E-08
FGL2	0.589283821	4.221426643	3.767694018	0.000193331
TSHZ2	0.589387246	2.233485386	5.687948624	2.72E-08
GNAZ	0.589429549	2.382332699	4.79707339	2.39E-06
MXRA5	0.589471577	5.536204291	4.203058588	3.35E-05
LRCH2	0.589663655	1.141358522	8.730162707	1.07E-16
TBKBP1	0.589733982	2.671437394	6.762410147	5.72E-11
ENPP2	0.589839592	3.961765373	4.789272653	2.48E-06
NPY	0.589850291	0.866705578	4.802377882	2.33E-06
NLGN4X	0.590066177	1.048251257	5.943218347	6.77E-09
TGFBR3	0.5900708	3.365676166	4.692813844	3.87E-06
PCDH17	0.590452766	2.635382987	7.149141191	5.14E-12
ECHDC3	0.591139072	2.605606893	3.559222937	0.000423395
EFNB3	0.591145755	0.991018408	7.501035318	5.29E-13
PLXNB3	0.592039253	1.700683121	4.5971439	5.99E-06
ATF3	0.592115866	5.12887338	4.751689241	2.95E-06
SH3BGRL	0.592316081	6.864955315	6.059046042	3.55E-09
TXNIP	0.59236743	8.103377234	4.854782955	1.82E-06
ADGRF5	0.592399764	3.676603674	6.872146864	2.92E-11
ABHD8	0.59302294	3.429915319	6.828896991	3.81E-11
CLDN23	0.593136155	4.419320658	4.09356187	5.28E-05
RN7SL8P	0.593287986	1.402083312	5.398680752	1.24E-07
GIHCG	0.593626328	2.062309661	7.343102699	1.48E-12
FSCN1	0.593933488	5.90101018	4.552166254	7.34E-06
P3H1	0.59408464	4.67147001	8.914143219	2.78E-17
MAF	0.594511677	3.855307397	6.167013143	1.92E-09
ABCA6	0.595006377	1.04631281	8.153735592	6.40E-15
SYPL2	0.595200001	0.947266174	7.609750741	2.58E-13
KCND1	0.595203513	1.551785142	8.769827465	7.99E-17
MBNL1-AS1	0.595219672	1.350765934	5.31688708	1.89E-07
RRAS	0.595590573	6.985236244	6.878849773	2.80E-11
NUMBL	0.59564698	3.965133548	7.543522659	4.00E-13
SLC1A7	0.596216204	1.177427433	6.416078773	4.55E-10
RNASE1	0.596220684	7.683325209	4.471103294	1.05E-05
PODNL1	0.596530545	2.507782322	5.558832158	5.40E-08
RNA5SP283	0.596578796	2.622938739	4.400960002	1.43E-05

AMPH	0.596723202	1.241875924	8.240625412	3.49E-15
ARMC9	0.59704924	2.715738878	8.461207682	7.36E-16
ABCA9	0.597213033	0.956674633	7.276057961	2.28E-12
AHNAK	0.59742701	6.248960396	5.565730782	5.21E-08
ITGB5	0.597687891	5.799622567	7.898186611	3.71E-14
CIDEC	0.59783865	2.060169498	3.690067598	0.000259963
LINC01116	0.598078719	1.389386201	6.932313115	2.01E-11
PDZD2	0.598393698	1.691870459	7.505245744	5.14E-13
LMCD1	0.59844878	2.748414503	8.069671688	1.15E-14
HTR2B	0.598503492	1.183834441	6.491350299	2.92E-10
SNCA	0.598620475	1.172509042	7.710495334	1.32E-13
DZIP1L	0.598735571	2.098866624	5.247563499	2.68E-07
CHN1	0.598864644	3.418341683	6.316288183	8.15E-10
CACNB2	0.599384612	1.52934299	6.215617548	1.46E-09
RNF212	0.599588915	1.166920185	5.497467172	7.45E-08
PLAAT5	0.599637775	1.276565068	4.72744553	3.30E-06
GALNT14	0.599888393	1.495252617	4.428555404	1.27E-05
DACT2	0.600595413	1.259020144	3.689744746	0.000260281
MMP23B	0.600668456	1.160161071	8.024597275	1.56E-14
PLPPR2	0.600830661	4.571805108	6.963790773	1.65E-11
AR	0.601521014	0.777963467	8.91347937	2.79E-17
SERPINF2	0.601776307	1.784796496	4.733732841	3.21E-06
SUSD5	0.601893515	0.769815249	9.145364749	5.01E-18
CDH2	0.602116818	1.330479975	5.657996318	3.19E-08
IFIT1	0.602347962	3.16161352	4.418848592	1.33E-05
BTBD19	0.602538571	2.374268147	7.947291802	2.66E-14
WTIP	0.602553383	1.282314614	9.770458593	4.30E-20
LDLRAD4	0.602672222	2.665557608	6.031340763	4.14E-09
PLA2G5	0.602827	1.10522702	6.843964217	3.47E-11
ANOS1	0.603000515	2.083422343	5.759155511	1.85E-08
NPNT	0.603016051	4.08053054	3.869685395	0.000130021
HSD11B1	0.603269831	2.287028258	5.204144035	3.33E-07
ERICH5	0.603486102	1.890225272	4.640807259	4.92E-06
MT1M	0.603534102	2.304181835	3.840804626	0.000145607
NAP1L5	0.604180787	1.990819981	8.181089517	5.29E-15
FARP1	0.604209762	3.950040983	6.66505042	1.03E-10
PHYHIP	0.604219037	1.143022085	8.391831512	1.20E-15
DIRAS3	0.604246078	1.35470924	6.287780987	9.62E-10
CCDC144NL-A	0.604325958	1.206809207	5.299066458	2.07E-07
MEIS3P2	0.604545757	1.022971804	8.585661666	3.02E-16
CLSTN3	0.604705859	4.5509423	6.564474097	1.89E-10
HAVCR1	0.604738081	1.085684609	4.234723945	2.93E-05
FGF14-AS2	0.60490786	0.914259083	7.639210341	2.12E-13
LIMCH1	0.604991771	2.634619025	6.297731847	9.08E-10
RTL8A	0.605556433	6.116733128	5.555701673	5.49E-08
MAGI2	0.605607684	1.172772104	9.185817053	3.70E-18
SLC29A4	0.606156803	1.898228595	4.109617434	4.94E-05
BCL6	0.606357827	4.499708691	7.21410756	3.40E-12
CCN5	0.606484759	0.919807585	6.593458635	1.59E-10
MAMLD1	0.606536196	1.875939851	7.103366483	6.87E-12
ZNF677	0.607500592	1.526749571	7.16143108	4.75E-12
FOS	0.607613759	7.685536321	4.175653961	3.76E-05
ST6GALNAC6	0.607701506	4.325574998	7.237876258	2.92E-12

ADAMTS4	0.607878196	2.981191999	6.055342823	3.62E-09
SNAI1	0.608766713	3.341528403	6.695295382	8.60E-11
PDE1B	0.608767743	1.409006859	7.3511478	1.41E-12
AC090877.2	0.608918225	0.876316307	8.697090487	1.35E-16
SELE	0.609108103	2.008344857	4.708629856	3.60E-06
NCKAP5L	0.609327959	3.570732251	8.049368065	1.32E-14
AP003071.3	0.609512532	0.921749588	6.576219207	1.76E-10
AVPR1A	0.609798566	1.377290958	7.719584159	1.24E-13
APOC3	0.610044975	1.223112124	3.187083877	0.001566821
TDRP	0.610336177	1.342050119	7.495069508	5.50E-13
CCDC136	0.610580182	1.515384786	5.007362065	8.77E-07
TBC1D9	0.610845025	3.538529224	7.052476525	9.47E-12
CPZ	0.610890819	0.861526737	9.481822383	3.96E-19
DKK2	0.610935395	1.408973509	6.100697341	2.80E-09
AC016747.1	0.610936781	1.983640144	6.917008609	2.21E-11
TEAD1	0.610993529	4.134883159	6.312481959	8.33E-10
DTX1	0.611555963	2.535256268	5.247547132	2.68E-07
NOTCH4	0.611977336	3.101808303	7.846031961	5.29E-14
WIPF3	0.612042264	2.289209642	4.756459989	2.89E-06
SMIM3	0.612419113	4.055670119	6.54138994	2.17E-10
ZDHHC2	0.612473572	3.192562734	5.131155056	4.79E-07
NR2F2-AS1	0.612714689	1.198954273	7.958924678	2.45E-14
RAB23	0.612849349	2.79553311	5.315711592	1.90E-07
UST	0.613260665	2.264667311	4.981228293	9.95E-07
PTPRU	0.613313166	3.900311778	4.046505996	6.40E-05
COL9A1	0.613390913	1.431841523	5.305959364	2.00E-07
RSPO2	0.61345299	0.827192349	6.824212713	3.92E-11
LPL	0.613936013	2.195852289	5.380819032	1.36E-07
SHE	0.614001614	1.680016919	8.230327807	3.75E-15
PON3	0.614172543	1.666721598	3.91578632	0.00010837
ORM1	0.614402936	1.628689925	2.661444226	0.008140008
FNBP1	0.614439902	4.346485979	4.748801193	2.99E-06
CYTL1	0.614490301	1.506558659	6.888879163	2.63E-11
ANGPT1	0.61455918	1.684460962	7.496762885	5.44E-13
PLA2G4C	0.614667552	2.661989917	5.767691015	1.77E-08
FHL3	0.61471135	5.05460309	7.264979129	2.45E-12
TCEAL8	0.614736127	5.433012044	7.28929544	2.10E-12
TMEM37	0.614920617	4.373942982	4.847638733	1.88E-06
CSGALNACT1	0.615244144	3.248426854	6.790161944	4.83E-11
GNE	0.61528024	3.920393562	4.819565095	2.15E-06
MYCT1	0.615602451	2.458015404	8.19714839	4.73E-15
PIFO	0.61568184	1.175084568	6.307556767	8.58E-10
BMP6	0.615769158	1.601276144	7.122643003	6.08E-12
TTYH2	0.615929508	2.34431397	5.977227335	5.60E-09
THBS3	0.616099518	3.728176473	8.160735504	6.10E-15
EPAS1	0.616238627	6.493598619	8.032386294	1.48E-14
AC053503.3	0.616487743	0.96053242	4.456726767	1.12E-05
ECEL1	0.616555062	1.267901791	5.193290824	3.52E-07
RNF217	0.616567789	1.510488857	8.262716566	2.99E-15
SLC19A3	0.616578669	2.384963589	3.318677426	0.00099972
HACD4	0.61682332	1.96903561	7.650794799	1.96E-13
BARX1-DT	0.616973479	0.715067328	7.259759208	2.54E-12
CSF1R	0.617491015	4.480028009	4.641052114	4.91E-06

BACE1	0.617503691	3.515984161	7.790170307	7.72E-14
TNFRSF11B	0.617759401	2.767758047	3.615021394	0.000344485
SLC34A2	0.617822008	2.030901026	3.075271439	0.00226887
RAMP3	0.617969595	4.038783113	6.477204547	3.18E-10
PROCR	0.61848994	5.220555244	5.377909358	1.38E-07
BMPR1B	0.618689452	1.044314495	6.544660433	2.13E-10
PAPSS2	0.618821962	4.182433788	5.892627737	8.95E-09
FES	0.618832477	3.248007859	7.150733512	5.09E-12
CHST13	0.619661565	1.895755373	3.913818066	0.00010922
PBX3	0.619765929	4.152777206	6.26912736	1.07E-09
KLHL29	0.619923244	2.142508766	7.050097426	9.62E-12
JAZF1	0.620103677	3.348220096	7.980675813	2.11E-14
PTGIR	0.620362755	2.306487797	7.970213963	2.27E-14
SCN7A	0.620392248	0.770908619	6.256526794	1.15E-09
TRO	0.620527484	1.276469939	9.041656492	1.08E-17
RGS7BP	0.620824508	1.026133144	8.553996305	3.80E-16
SBSPON	0.620935243	2.010298072	4.530089736	8.10E-06
ZNF528-AS1	0.62104259	1.566443386	6.639787697	1.20E-10
CHST7	0.621302116	2.228171515	8.680371179	1.53E-16
ZNF512B	0.621605127	2.867644904	5.682648387	2.80E-08
PGF	0.621743376	2.831265104	7.509835522	4.99E-13
PRAME	0.622191474	1.88968741	2.942764985	0.003470346
SESN3	0.622383206	2.962137169	5.089406526	5.88E-07
ARHGAP6	0.622715584	1.987711605	6.122943737	2.47E-09
HORMAD1	0.62289023	1.041908408	4.524458291	8.31E-06
EMP3	0.622977452	5.95897738	6.29120539	9.43E-10
PLXND1	0.623066918	5.249196251	6.32762049	7.63E-10
FRAS1	0.6232493	1.96813136	4.772704349	2.68E-06
METTL7A	0.623718909	4.745164878	5.408167614	1.18E-07
CHL1	0.62388803	1.28703583	6.675099051	9.72E-11
STAB1	0.624340424	3.938045173	5.974438269	5.69E-09
VPS35L	0.624560365	3.919444957	6.633114017	1.25E-10
PGM5-AS1	0.624607186	0.964905954	4.737124603	3.16E-06
IGDCC4	0.625737528	1.616826856	7.605719106	2.65E-13
GPR183	0.626101151	3.947783483	5.185006903	3.67E-07
FGF18	0.626223701	1.895846542	5.869154062	1.02E-08
SSBP2	0.62627253	1.935721095	7.065609132	8.72E-12
CDON	0.626942258	1.590609678	6.36258996	6.23E-10
VLDLR	0.626965464	1.470359949	6.99507744	1.36E-11
PCDHGB7	0.627233354	1.542869399	7.732281593	1.14E-13
CSRNP1	0.627501353	4.741259959	7.471239783	6.43E-13
RARB	0.628161189	2.250571736	7.909111575	3.45E-14
FGD1	0.628392515	2.700225551	7.465202988	6.69E-13
TRPS1	0.628754026	2.122543204	6.324071789	7.79E-10
GNA14	0.629125982	2.327774376	6.028598249	4.21E-09
HAS1	0.629252988	0.787815126	7.136668027	5.56E-12
SIPA1L2	0.629507314	3.545832497	6.385418223	5.45E-10
ITPR1	0.629634846	2.5671232	6.610095187	1.44E-10
CD200	0.629794784	3.266666656	6.924853712	2.10E-11
CARTPT	0.629799207	0.916974364	3.909478902	0.000111116
AC093010.2	0.630131503	4.103296943	6.611636106	1.43E-10
NCAM1	0.630497658	1.566184215	4.914014877	1.37E-06
NOVA1	0.631368732	0.981838796	7.155029286	4.95E-12

SEPTIN4	0.631636162	2.226009599	9.2370136	2.52E-18
BHLHE41	0.632062592	4.123554499	4.589844172	6.20E-06
CALCRL	0.632105381	3.583722058	6.370173294	5.96E-10
ADAMTS9	0.632324536	2.872232722	7.007553899	1.26E-11
FBXL2	0.632385035	1.804159338	8.451052124	7.91E-16
CAPS	0.632404086	2.954371516	5.052905147	7.03E-07
AP003555.2	0.63309538	2.527940557	3.940053377	9.84E-05
BCL6B	0.633390641	2.966443119	7.574038655	3.27E-13
RAP1GAP	0.633512017	3.880358702	4.06720046	5.88E-05
LINC00392	0.633966531	0.686320434	3.681265676	0.000268756
TMEM130	0.634257802	1.0689503	6.901422636	2.43E-11
ARMCX3	0.634270324	4.380881277	7.145880788	5.25E-12
MMP17	0.634438628	1.61686824	6.711227916	7.81E-11
SLC22A3	0.634489322	2.817017295	4.401228631	1.43E-05
KLHDC8B	0.634688866	3.457617867	7.767849511	8.97E-14
CHST15	0.634734454	3.940460036	5.243979493	2.73E-07
CHI3L2	0.634863265	1.847289476	5.055462781	6.94E-07
SLC12A4	0.63509055	4.145071348	8.128086161	7.65E-15
NPTX2	0.635142278	1.338259509	5.758971207	1.86E-08
SEMA3C	0.635626266	4.567773229	4.631274663	5.13E-06
SYN1	0.63570466	1.599084419	7.10091253	6.98E-12
NXPH3	0.635905146	1.076977265	5.929789317	7.29E-09
TRABD2B	0.636188175	1.208740065	7.671962956	1.71E-13
NMNAT2	0.636339221	1.337127561	5.872039746	1.00E-08
ISYNA1	0.636360963	4.451212453	4.923925839	1.31E-06
ARHGEF15	0.636564944	2.624188905	7.702008743	1.39E-13
SNAI2	0.636619287	3.353204232	6.414852894	4.59E-10
MACROH2A2	0.636748837	3.298686061	4.625416894	5.27E-06
ALDH1A2	0.636756213	0.721730899	7.243725023	2.81E-12
DNAJB4	0.636832711	3.700423189	7.864982544	4.65E-14
F2RL2	0.636964499	3.458353092	4.867873361	1.71E-06
APOLD1	0.637132698	3.663229971	6.292362307	9.36E-10
MAP7D3	0.637136293	2.532685327	7.181614895	4.18E-12
SEMA6C	0.63714674	2.102415515	7.682468248	1.59E-13
TBX2	0.637182166	3.105626078	7.564905361	3.47E-13
FNDC5	0.637308199	1.077490596	7.184842815	4.10E-12
CYP2U1	0.637650888	2.221100922	7.997486309	1.88E-14
DMPK	0.637945072	4.285603411	5.408743205	1.18E-07
HEPACAM2	0.638144921	1.742070715	3.138271405	0.001844086
EHD3	0.638576774	2.704213714	7.237206586	2.93E-12
AC010332.1	0.638620481	1.294985712	6.222024068	1.40E-09
TSPAN4	0.6388157	4.178440248	7.626798414	2.30E-13
TPBG	0.638828004	3.209201673	6.470238276	3.31E-10
TMEM121	0.638929775	1.761966615	7.21738631	3.33E-12
CORO2B	0.639040306	1.160230122	8.03392608	1.47E-14
PBXIP1	0.639745729	5.873836767	7.712833054	1.30E-13
MFSD4A	0.640113347	2.792884484	3.945009844	9.65E-05
CBLIF	0.640162532	1.716577555	2.707130842	0.007120248
SCGN	0.640466839	0.951694383	4.485171437	9.90E-06
GLIPR1	0.640526938	3.18951463	6.056739411	3.59E-09
CSRP2	0.640879481	2.234736818	7.938922095	2.81E-14
PRRX2	0.64092892	3.406655183	4.372073937	1.63E-05
LGALS1	0.640951367	8.953707898	6.017851896	4.47E-09

NCS1	0.641144108	3.585508277	4.674033097	4.22E-06
FAM198B-AS1	0.641441304	1.589371455	6.479850436	3.13E-10
GGT7	0.641619308	3.609044225	5.265503804	2.45E-07
PTGFR	0.642376711	1.156956726	7.513274308	4.88E-13
PI15	0.643094558	1.522937819	5.027186584	7.97E-07
TCEA2	0.643145466	3.779961057	6.209065971	1.51E-09
GSPT2	0.643188746	2.02457961	6.091287946	2.96E-09
SUGCT	0.643361729	1.762083686	7.573578223	3.28E-13
RGL1	0.643381427	3.114895771	6.874663653	2.87E-11
SCGB3A1	0.643682002	1.484949162	3.123026371	0.001939551
PARVA	0.643843493	5.433131819	7.899812565	3.67E-14
RASSF2	0.643892223	3.393144843	5.505188923	7.15E-08
PHGDH	0.643929247	3.98940175	4.154071436	4.11E-05
ARHGAP24	0.64404227	2.672177849	7.219541065	3.28E-12
JCAD	0.644301207	3.099045076	6.591462096	1.61E-10
NUAK1	0.64503404	2.656304832	8.200197002	4.63E-15
LBP	0.645224399	0.858417676	4.839474803	1.96E-06
CLCF1	0.645515001	3.603901216	6.906309841	2.36E-11
PMEPA1	0.64574393	5.963640391	4.559555991	7.10E-06
TRIL	0.645871934	2.0887518	6.789933726	4.83E-11
FPR1	0.646144101	2.762151017	4.959711826	1.10E-06
CHST1	0.647152584	2.535555694	7.094476061	7.27E-12
PREX2	0.647191648	1.548264748	8.597958842	2.77E-16
CCL2	0.647234854	5.730043117	4.980199769	1.00E-06
ACHE	0.647360147	3.25739626	3.682713396	0.00026729
PYGL	0.647711998	3.600388775	5.014380897	8.48E-07
CP	0.647937317	2.288456208	3.732716592	0.000221066
PENK	0.648099699	1.056390043	4.526255305	8.24E-06
GRP	0.648248839	0.948776629	5.450042849	9.53E-08
ZNF542P	0.648333636	1.729106075	7.408676795	9.68E-13
EVA1B	0.648454993	4.604706652	7.742607604	1.06E-13
PLPP3	0.648864707	5.140895858	8.199988863	4.64E-15
PLEKHG2	0.649950476	3.726207264	7.917231474	3.26E-14
ENG	0.650162502	6.538886069	7.625449327	2.32E-13
IGHV4-61	0.65078513	3.694573185	2.965882302	0.003225833
TGFB1	0.650998049	6.169208731	6.995608575	1.35E-11
BNIP3	0.651181711	3.269076247	5.530522663	6.27E-08
CARD11	0.651699903	3.515409791	4.278480207	2.43E-05
PTHLH	0.651716137	1.78774861	6.344247946	6.93E-10
IGF1	0.651733202	0.862123073	7.219187341	3.29E-12
NPC1L1	0.652005075	2.349388202	3.28861752	0.001109216
TCN2	0.652280791	4.203825216	6.545973935	2.11E-10
FKBP7	0.652379989	3.128554815	9.510369746	3.18E-19
CXCL6	0.652484217	2.328088334	3.705765548	0.000244951
GSTM3	0.652596514	4.050103213	4.783171442	2.55E-06
ZNF528	0.652987941	1.793645219	6.28268945	9.90E-10
AFAP1L1	0.653299025	2.494306212	8.788660465	6.96E-17
FAM171B	0.653327179	1.848268168	8.499695751	5.60E-16
MEF2C	0.653367911	3.051667417	6.565233559	1.88E-10
CCN4	0.654372119	2.542800061	5.767020651	1.78E-08
TGFBR2	0.654785593	6.198739712	6.879175501	2.79E-11
CDHR2	0.655056923	3.541783712	3.2559422	0.001240807
SEMA7A	0.655358055	3.037146614	6.016229937	4.51E-09

IGHV3-11	0.655712791	5.056331227	2.687164981	0.007550823
RAB32	0.655727787	4.779802357	6.780745009	5.11E-11
TM4SF5	0.655805537	4.828828095	3.667084423	0.000283512
HSPA12B	0.656277763	2.541442281	7.883915355	4.09E-14
LRRC4B	0.657142222	1.621050535	7.707415496	1.35E-13
PPP2R3A	0.657337551	2.277151251	6.872466715	2.91E-11
FGL1	0.657407244	0.663959925	4.94613559	1.18E-06
DIP2C	0.657424961	3.033715852	7.845063329	5.32E-14
CRTAC1	0.657523919	0.755746826	7.440921134	7.84E-13
FBXO27	0.657564733	1.798768311	5.758359266	1.86E-08
KIF3C	0.657744993	2.315786363	7.248057567	2.73E-12
LPP	0.657840155	5.488422622	5.891227594	9.02E-09
TRPC1	0.658352032	1.343490044	8.766914091	8.16E-17
NAP1L3	0.658412087	1.076942249	8.730439816	1.06E-16
ANXA6	0.659447704	6.096283785	5.847342888	1.15E-08
NEU1	0.660638986	4.178093756	5.266534344	2.44E-07
ADAMTS12	0.660931662	2.752358564	5.48748868	7.85E-08
THRB	0.661136664	2.369397299	5.719733913	2.29E-08
KLHL5	0.661317773	4.366631158	6.898562697	2.48E-11
COL4A1	0.661445842	7.402854759	6.423868368	4.35E-10
TENM3	0.661520905	1.176972833	6.797243988	4.62E-11
ST3GAL1	0.6616048	3.959815576	6.46990427	3.32E-10
MARK1	0.661959379	1.671409913	6.270595275	1.06E-09
MAPK4	0.662116374	0.858617467	5.957082476	6.27E-09
ROR1	0.662221044	2.678342497	6.478332707	3.15E-10
HHEX	0.662248601	3.762128101	5.853207853	1.11E-08
COL15A1	0.662736443	5.826720472	5.391281949	1.29E-07
PLEKHA4	0.662991338	4.541973599	5.946506883	6.65E-09
NPR3	0.663025745	0.93888506	7.584808126	3.04E-13
RFTN1	0.663087952	4.529558295	6.540061985	2.19E-10
SOCS3	0.663499379	6.077822195	6.510189197	2.61E-10
SUSD2	0.663612673	2.832810695	6.295303287	9.21E-10
ISLR2	0.663815809	1.122391414	8.480608974	6.41E-16
CCL11	0.664071041	4.013766558	4.05377594	6.22E-05
NR3C1	0.664689829	3.642201913	7.114195774	6.42E-12
RASIP1	0.664896693	3.129868582	7.914376829	3.32E-14
PALMD	0.665528048	2.17026706	7.28667219	2.13E-12
SPSB1	0.665539405	4.927924902	8.831644428	5.09E-17
UGT2B15	0.665546735	1.749277543	3.987581449	8.13E-05
DCLK2	0.665791506	1.522388257	7.402758859	1.01E-12
RGL3	0.665796459	2.399836703	4.368194306	1.65E-05
NPY1R	0.665903128	0.756472052	7.228984915	3.09E-12
PEAR1	0.665910743	2.394418774	8.301966118	2.27E-15
PRDM5	0.665965419	1.153186664	9.580815956	1.86E-19
LTBP4	0.666853119	6.355588916	6.344043591	6.94E-10
CHST10	0.66699818	2.070171064	7.565098364	3.47E-13
HOXC10	0.667019696	3.066624495	2.934286064	0.003564194
PTPRS	0.667500386	3.327456563	4.804997152	2.30E-06
LGI4	0.667785075	1.619356652	7.604199644	2.68E-13
FZD1	0.668304408	3.497495959	7.575457263	3.24E-13
TANC2	0.668383949	2.539638402	6.689822504	8.89E-11
SYT13	0.669124525	3.69291319	3.393358391	0.000769665
AC239859.5	0.669236808	1.594015548	6.56758995	1.86E-10

SLC4A3	0.669240368	1.524783052	5.910339819	8.12E-09
RGN	0.669268441	1.676146267	5.096882171	5.67E-07
SH3RF3-AS1	0.669459454	1.513388108	8.797343883	6.53E-17
EMID1	0.670094906	2.667955747	5.640438075	3.51E-08
FZD4	0.670496426	3.032305941	8.480849048	6.40E-16
PRDM8	0.670802839	1.667026103	6.956641903	1.73E-11
SOBP	0.670868049	2.296374479	5.957370731	6.26E-09
SNHG14	0.671032443	1.821354636	5.910244669	8.12E-09
NINL	0.671319207	2.545265688	5.600205165	4.34E-08
HGF	0.672806139	1.772799259	7.181350424	4.19E-12
PRSS1	0.673236848	2.156814752	2.846824889	0.00467674
SERP2	0.673247594	1.904674111	6.740530987	6.54E-11
HSD17B14	0.673410038	3.094619059	6.217328941	1.44E-09
TSPAN7	0.673428334	3.321357325	4.924568342	1.31E-06
PXDC1	0.673659477	4.472613896	7.953549826	2.54E-14
KIAA1755	0.674220455	1.906911259	7.698717635	1.43E-13
POPDC2	0.674239712	2.145463142	4.861752921	1.76E-06
LXN	0.674705148	4.744816061	6.638080153	1.22E-10
OLFML2A	0.674828524	3.544706424	7.021261808	1.15E-11
C1QTNF2	0.674843285	1.183333491	9.646137481	1.12E-19
FZD7	0.675059271	3.813798965	5.815310533	1.37E-08
ZEB2	0.675278497	3.204259749	6.704071131	8.16E-11
PCDHB4	0.67541095	1.26146141	9.477608705	4.09E-19
ROBO1	0.675450918	3.460863684	6.478428383	3.15E-10
C1QTNF3	0.675475887	2.07122338	6.050433181	3.72E-09
GLRB	0.676573027	1.079901877	8.993940275	1.54E-17
VIPR2	0.676700473	0.87494463	7.688039299	1.53E-13
EMCN	0.67679568	2.84439839	7.632775715	2.21E-13
P2RY14	0.677240448	2.080163595	6.072409155	3.29E-09
EBF1	0.677414804	2.436935084	7.161236603	4.76E-12
ARHGAP31	0.677903111	2.952340691	7.701030412	1.40E-13
PLXDC1	0.678335958	2.889126261	8.637039234	2.09E-16
LYVE1	0.678519676	1.715905629	6.519143257	2.48E-10
AQP1	0.678751401	6.299607361	4.401560604	1.43E-05
CERS4	0.67876723	3.158499829	4.793002035	2.43E-06
NPTX1	0.678825471	0.992358987	5.004499434	8.90E-07
ANO5	0.679259791	1.366576113	6.731463435	6.91E-11
SEMA3E	0.67988887	1.213574299	5.781963757	1.64E-08
PRAF2	0.679976466	4.631567536	8.375168616	1.36E-15
SPP1	0.680150825	6.343478345	3.037290015	0.002566711
CPVL	0.680812211	4.72676845	4.819200675	2.15E-06
HSPA1A	0.681239826	5.311600138	4.548186238	7.47E-06
SLC16A7	0.681444919	1.553383978	7.562794105	3.52E-13
QPCT	0.681633495	3.276259112	4.620592398	5.39E-06
PRICKLE1	0.681718609	1.349453492	8.674316762	1.60E-16
OLFM2	0.682264328	3.416357143	5.158605377	4.18E-07
FRY	0.683737857	2.178501353	8.555455719	3.76E-16
WNT5A-AS1	0.683895708	2.117476902	6.701044434	8.31E-11
GALNT17	0.684504397	1.146055177	9.279677523	1.83E-18
LAMB2	0.684718013	6.04206467	7.262571449	2.49E-12
SEMA3G	0.685196764	2.23749793	7.59464347	2.85E-13
TCIM	0.685651736	4.985774051	5.065624503	6.61E-07
SULF2	0.685891692	6.04512213	5.778602205	1.67E-08

MIR99AHG	0.686029097	1.050701423	8.338141246	1.76E-15
SPIRE1	0.686252098	2.594971248	6.837957276	3.60E-11
PYGO1	0.686364089	1.35373125	8.290202714	2.47E-15
AC254633.1	0.686470789	1.653651519	7.734010437	1.13E-13
GPR176	0.686548758	2.528154167	8.045209763	1.36E-14
SNCAIP	0.686938126	1.594641625	5.265599273	2.45E-07
AL139393.3	0.686988429	1.651071428	7.479276242	6.10E-13
SNORD116-4	0.687048448	1.096415096	5.989116443	5.24E-09
TNN	0.687091691	0.860685511	7.331285063	1.60E-12
PLEKHH2	0.687162809	2.0964383	6.833297926	3.70E-11
GSTM5	0.687271456	1.128291442	8.651992797	1.88E-16
PYGM	0.687520154	1.253208987	6.204697993	1.55E-09
XPNPEP2	0.687621357	1.946935879	3.756724219	0.000201655
GPRASP1	0.688207508	1.477679893	7.079309114	8.00E-12
MTSS1	0.688636111	3.325265615	6.012975249	4.59E-09
CD36	0.688998941	2.315702575	6.0425763	3.89E-09
TNNC1	0.6891207	2.59007353	4.260395667	2.63E-05
GLI2	0.689203421	2.205781409	6.623672266	1.33E-10
SCN1B	0.689489578	1.855129617	8.576360344	3.23E-16
SLC2A4	0.689732216	1.719379331	4.979263312	1.00E-06
TEK	0.689778516	2.419532568	8.036929595	1.44E-14
MMP19	0.689845669	2.897397628	7.375526016	1.20E-12
PLPPR4	0.689947972	1.404295403	7.579300393	3.16E-13
KCTD15	0.691557266	3.073073559	6.99734098	1.34E-11
MOCS1	0.69236358	2.502877242	8.132911064	7.40E-15
SYNE1	0.692685152	2.089154706	6.658438284	1.08E-10
CALHM2	0.6931162	3.946101451	7.84396509	5.36E-14
TPD52L1	0.693188943	2.968317357	4.596149303	6.02E-06
RNF180	0.693233324	1.229236058	9.6152694	1.43E-19
MMP14	0.693653347	7.189236417	6.980525705	1.49E-11
MGLL	0.693663196	5.587139106	6.285405561	9.75E-10
CST6	0.693667077	1.448709946	4.263669379	2.59E-05
PLP1	0.693689266	0.85415249	6.084717761	3.07E-09
AP003071.4	0.694254561	1.144368493	6.570402312	1.82E-10
DCDC2	0.694585207	1.372574413	5.353409181	1.57E-07
CDH5	0.694810172	4.533945578	7.423924101	8.76E-13
TCF4	0.694903659	3.942346562	7.444894997	7.64E-13
LRRN2	0.694985925	1.467482979	7.280413063	2.22E-12
GPSM1	0.695476238	2.671946686	6.546884119	2.10E-10
GRASP	0.695668922	3.251650855	8.194524946	4.82E-15
TNFSF12	0.696293784	4.231051161	7.763251622	9.25E-14
ZFPM2-AS1	0.69676666	1.390875593	5.520053464	6.62E-08
FXYD1	0.696902341	1.227212876	7.63464478	2.19E-13
TMEM204	0.696915998	4.128138803	8.732465968	1.05E-16
MYOM1	0.696977474	1.501901619	6.596005123	1.57E-10
CPM	0.697543679	3.410480496	5.596960499	4.42E-08
HOXB2	0.697568103	3.799473954	5.900726833	8.56E-09
PCP4L1	0.697985795	1.203193309	4.854806914	1.82E-06
CD302	0.69810761	2.574532968	7.291384408	2.07E-12
ARHGEF4	0.698357668	2.056230578	5.109837007	5.32E-07
TGFB2	0.698401915	1.946368017	7.110538922	6.57E-12
CCDC69	0.698458013	4.30831658	5.217108817	3.12E-07
C5AR1	0.698527995	4.15615209	5.88032472	9.58E-09

ZBTB10	0.698790713	2.381996577	7.026644475	1.11E-11
COL18A1	0.698790772	5.929679336	6.499667925	2.78E-10
CFL2	0.69947911	3.154676438	5.790926238	1.56E-08
ARL14	0.699696399	4.042415679	3.899273957	0.000115699
FHL5	0.699710258	1.559549374	8.482090085	6.35E-16
ARHGEF10	0.700121223	3.206400525	7.041762341	1.01E-11
SNED1	0.700141035	2.453218577	7.457051312	7.06E-13
KLF7	0.700424108	3.067575055	8.066643375	1.17E-14
SPINK1	0.70043804	7.796272811	2.800215389	0.005390861
TACC1	0.700598775	5.264904226	6.980727062	1.49E-11
GPRC5B	0.701447277	4.221875164	4.41977334	1.32E-05
TSPAN11	0.701649754	2.311858761	6.29187969	9.39E-10
CD34	0.701677418	3.928719155	8.164932583	5.92E-15
TENT5B	0.702003935	2.221682976	4.738668042	3.14E-06
VEGFC	0.702258256	3.138720265	8.509116342	5.23E-16
CXCL14	0.703153504	5.794833381	3.347817266	0.000903245
CTSG	0.70340902	1.000586934	6.396382596	5.11E-10
CPQ	0.703769328	4.621451715	7.567970323	3.40E-13
MEIS1	0.704115285	3.023658229	6.572660504	1.80E-10
DIXDC1	0.704866821	3.074388377	6.071915157	3.30E-09
AQP3	0.705214377	4.022043338	4.020956362	7.11E-05
PDE7B	0.705274488	1.437837541	7.262830735	2.49E-12
PBX1	0.705347325	3.431123498	6.470253247	3.31E-10
B3GNT7	0.706129803	4.740968311	4.300109813	2.22E-05
CYP7B1	0.706160864	1.805231624	7.196014698	3.81E-12
SMTN	0.706232908	5.576220374	5.508293958	7.04E-08
SEMA6B	0.706334004	3.336757545	8.021888748	1.59E-14
SV2A	0.706777404	1.768627879	7.560406391	3.58E-13
CDH6	0.706979463	1.803981456	10.34740808	4.58E-22
BCHE	0.70719377	1.084295827	5.799474921	1.49E-08
ASB2	0.707290119	2.634797183	4.419125953	1.32E-05
PLXNA4	0.70732827	1.074635314	7.755565456	9.74E-14
VANGL2	0.707982078	2.566011292	4.768544377	2.73E-06
MAP9	0.708125242	1.740715425	7.83346114	5.76E-14
FADS1	0.708127097	3.436710238	5.995669863	5.06E-09
TMEM45A	0.70883827	2.287804608	7.856580387	4.92E-14
CST1	0.708886146	5.95418184	2.637693123	0.008720436
MAGI2-AS3	0.709629479	1.693205309	8.8462298	4.57E-17
CYYR1	0.709895797	3.232727114	8.491479139	5.93E-16
ANKRD6	0.710652184	1.571292969	9.630562716	1.27E-19
ERRFI1	0.711885241	5.566452761	7.019587123	1.16E-11
ADAMTS2	0.712098798	4.177460027	5.001122742	9.04E-07
RIMKLB	0.712723267	2.031947036	6.491517151	2.92E-10
TGM2	0.713236989	6.957503123	5.253729473	2.60E-07
PCOLCE2	0.713238476	1.191683102	6.949648981	1.80E-11
SOGA1	0.713414903	3.506589602	6.810018063	4.27E-11
TCEAL9	0.713804071	6.195216597	6.447810027	3.78E-10
GJA4	0.713813024	3.845224985	7.838109166	5.58E-14
GNG11	0.713862097	3.690838631	8.394433989	1.18E-15
HHIP-AS1	0.713906449	1.259294089	8.199972965	4.64E-15
FAM13C	0.714520704	1.575396048	8.052226957	1.29E-14
LEPR	0.714565697	1.96161916	7.592321408	2.90E-13
HDGFL3	0.714603998	1.95984037	8.108410959	8.77E-15

ZBTB47	0.715179529	3.421967797	7.997766175	1.88E-14
RASSF8-AS1	0.715415039	1.445554924	8.663398692	1.73E-16
MSC	0.71546266	3.4259818	7.246539222	2.76E-12
AP005018.2	0.716246857	1.518307573	5.16708601	4.01E-07
MYADM	0.716297284	7.252088599	7.34324646	1.48E-12
TEAD2	0.716960939	4.138228496	7.08097057	7.92E-12
ADAMTS10	0.717415267	2.078229565	8.26222773	3.00E-15
FZD8	0.717631768	3.397755671	6.193855466	1.65E-09
IGHV1-69D	0.717688455	5.55839643	2.778778085	0.005751381
SAMD4A	0.717869926	2.85901451	7.173915769	4.39E-12
PTGER2	0.717962897	3.123380339	5.608044417	4.16E-08
IL34	0.718061378	2.56155845	7.017382749	1.18E-11
PIP4P2	0.7188329	2.853005576	8.300345188	2.30E-15
MSLN	0.718899908	5.285462307	2.623517282	0.009084312
FOXC2	0.718943893	1.448246491	7.804471456	7.01E-14
CFH	0.719330087	4.065597735	5.740971374	2.05E-08
NR2F1-AS1	0.719487771	1.520974859	9.109995193	6.52E-18
KCTD12	0.720071587	5.463614943	5.792820557	1.55E-08
RNF144A	0.721088924	2.979039612	7.993564075	1.93E-14
DUSP5	0.721188266	4.010042922	6.111179326	2.64E-09
QKI	0.721263244	3.823711197	8.387162007	1.25E-15
KIAA1324L	0.721502159	1.678293723	6.680628097	9.40E-11
IGHG4	0.721876439	7.288714505	2.875905441	0.00427592
PXDN	0.722342608	4.72526551	7.078257367	8.05E-12
VIM	0.722669223	8.434524569	7.855157174	4.97E-14
POSTN	0.723290244	6.805252688	4.741704632	3.09E-06
FUZ	0.723295052	3.091466738	6.261163234	1.12E-09
GPNMB	0.723583707	6.267851558	4.85272021	1.84E-06
REEP1	0.72412459	1.927870888	5.509258506	7.00E-08
SEPTIN5	0.724794322	2.1887535	7.526485793	4.47E-13
MPV17L	0.724797345	1.922395303	5.779002675	1.67E-08
CPT1C	0.724892047	1.935215773	7.438946327	7.94E-13
A4GNT	0.725252503	1.64708409	3.821115524	0.000157227
PAMR1	0.725377689	2.010357069	8.489615878	6.01E-16
IGFBPL1	0.725682148	1.456204523	4.974884201	1.03E-06
ZNF331	0.727394601	2.602046724	7.143121321	5.34E-12
APLP1	0.727584049	2.44996442	4.776276838	2.63E-06
FBXL22	0.728167328	1.884962595	6.310570833	8.43E-10
ECM2	0.72843801	2.851692656	7.684693727	1.57E-13
STMN2	0.728526006	1.083833271	6.198125623	1.61E-09
PCBP3	0.728812948	1.807673286	6.780174713	5.13E-11
CADM3	0.729197668	1.030787006	6.648981806	1.14E-10
CSRP1	0.729378589	6.042899297	6.11901521	2.53E-09
RAI14	0.730064186	4.747297339	9.288116178	1.72E-18
FAM162B	0.730227413	1.668656769	10.67859608	3.18E-23
PKD2	0.73189511	3.685243862	8.139908243	7.05E-15
FAM174B	0.731913753	3.702816297	6.689450403	8.91E-11
OLFM1	0.732254276	2.657427319	5.66937157	3.01E-08
FGD5	0.732290643	3.198651928	7.801911235	7.13E-14
GSDME	0.732333129	2.121290633	7.397574865	1.04E-12
DZIP1	0.732339053	1.666787829	8.745633211	9.52E-17
ANGPTL4	0.733185277	3.244769841	5.655694869	3.23E-08
TSPAN9	0.733196119	3.934597373	8.553476954	3.81E-16

ZNF134	0.73325671	2.455784158	7.525732698	4.50E-13
PHLDA3	0.733466019	4.703559392	5.880399998	9.57E-09
TUB	0.733717937	1.06355165	9.059869078	9.47E-18
CRIP2	0.733900417	5.904140669	7.850930297	5.12E-14
FOXS1	0.734131881	2.931518408	6.557286194	1.97E-10
ZFHX4	0.734138708	1.139994432	8.299455149	2.31E-15
DBN1	0.735459534	5.192467373	6.97205507	1.57E-11
PLAC9	0.735628716	3.270206378	6.735092421	6.76E-11
GADD45B	0.735792893	4.873249507	7.705435803	1.36E-13
TMEM88	0.736506356	3.104973141	7.727166248	1.18E-13
DTX3	0.736584353	3.183459532	6.659707752	1.07E-10
ITGA1	0.73685659	4.394660145	7.046272986	9.85E-12
NHSL2	0.73827055	1.318957278	7.927705065	3.04E-14
COX7A1	0.738991064	3.843172027	7.723669927	1.21E-13
LZTS1	0.739228136	2.401811946	7.771324003	8.76E-14
ID4	0.739337864	3.278194216	6.301541694	8.88E-10
CREB3L3	0.739474601	2.05187658	3.924532349	0.000104668
FAM229B	0.739785459	1.839302139	9.470751581	4.31E-19
TMEM35A	0.740153657	1.043507084	6.611083335	1.43E-10
MCC	0.740242553	2.243094892	8.089107811	1.00E-14
NR2F2	0.740640335	5.333147403	8.06760244	1.16E-14
NACAD	0.740717757	1.234425453	8.734725109	1.03E-16
VSIG2	0.741055623	5.143663858	3.034468659	0.002590209
NBEA	0.741516922	1.284328561	7.465015539	6.70E-13
EFNA5	0.741752616	2.122327006	7.228886033	3.09E-12
PKIG	0.74219984	5.381841154	8.231252909	3.73E-15
BEND5	0.742786169	1.249356374	8.43785474	8.69E-16
DCLK1	0.742959328	1.262877956	6.979674977	1.50E-11
MXRA7P1	0.743021803	1.983404967	7.177066616	4.30E-12
P4HA3	0.743296796	1.786880941	9.084501473	7.89E-18
GSN	0.743853521	6.892475502	7.373355582	1.22E-12
GJC1	0.743871716	2.11699381	7.442893522	7.74E-13
PLIN2	0.744367894	5.374833959	6.966880396	1.62E-11
RHOB	0.744754035	7.840353873	6.922763381	2.13E-11
AF001548.1	0.745065669	1.153235841	5.768041174	1.77E-08
TCF21	0.745590143	1.844846742	8.603641071	2.66E-16
PTCH2	0.746312514	1.577459954	6.747399337	6.27E-11
PECAM1	0.746423448	5.533587733	7.530546995	4.36E-13
DIPK2B	0.746430611	2.810364794	9.090962881	7.52E-18
DLG4	0.746590255	2.479125838	9.603661793	1.56E-19
RARRES1	0.747405259	4.594172856	4.498144123	9.34E-06
DAB2	0.748515988	4.867082843	8.01736919	1.64E-14
PCDH18	0.748667489	3.251415538	8.149949776	6.57E-15
SHISAL1	0.748688701	1.294542167	6.054952769	3.63E-09
EDNRB	0.748832702	2.967966973	7.487034264	5.80E-13
CD109	0.749042565	2.320264026	6.430834375	4.17E-10
EPDR1	0.74924136	3.180596957	6.050711482	3.72E-09
RAB3B	0.749288668	1.426113433	7.06842837	8.57E-12
TP73-AS1	0.749378017	1.877307016	8.325568262	1.92E-15
CLEC14A	0.749737513	4.022884875	8.559639987	3.64E-16
C20orf194	0.749757662	2.698918007	7.696793515	1.44E-13
TIE1	0.749943575	3.675004957	8.222515541	3.96E-15
GNB4	0.750197405	3.394290961	7.540684543	4.07E-13

PNMA2	0.750734771	1.866725008	6.164635394	1.95E-09
PDGFC	0.750953514	3.100274137	8.606109691	2.61E-16
TF	0.751250088	0.885831984	4.694648803	3.84E-06
LIX1L	0.751330746	4.026284154	7.752181197	9.96E-14
ALPL	0.751362845	2.895376978	6.646307499	1.16E-10
HBB	0.751422541	5.072585188	4.004735985	7.59E-05
PDZRN4	0.751876915	0.988437001	6.079529698	3.16E-09
EDIL3	0.752086372	3.878759874	7.626792847	2.30E-13
GLI3	0.752289527	1.793166062	7.867346319	4.58E-14
SCTR	0.752330622	1.133193348	5.858923241	1.08E-08
CC2D2A	0.752920313	2.307141957	9.725247947	6.11E-20
NEGR1	0.753004424	1.358852907	7.399874848	1.02E-12
APLNR	0.753559796	4.324577045	6.019691571	4.42E-09
ALKAL1	0.75386087	1.633089229	6.937389259	1.95E-11
TMEM59L	0.754250065	0.961592733	8.365007227	1.46E-15
VGLL3	0.754278627	1.697477327	7.906740799	3.50E-14
TPM1	0.754992312	6.945436547	6.801881637	4.49E-11
ERG	0.755240437	2.611866299	9.291582766	1.67E-18
ABLIM3	0.755280442	3.033292877	6.56304967	1.91E-10
F2R	0.755708563	5.36499257	7.796951613	7.37E-14
TFPI2	0.755902284	1.632498593	5.836116602	1.22E-08
SCUBE2	0.756608008	1.654255011	7.053450924	9.42E-12
CDK14	0.757338741	2.78960261	7.148463026	5.16E-12
PRKAR2B	0.757866637	2.7444142	6.245580003	1.23E-09
COL6A1	0.758158552	7.654024484	6.476655975	3.19E-10
CNTN4	0.758235928	1.540517526	9.244445946	2.39E-18
RAMP2	0.758910658	5.893828373	7.992705829	1.95E-14
AGTR1	0.75927522	1.009771186	6.17846548	1.80E-09
TIMP1	0.759480803	9.888916662	7.373526072	1.22E-12
CILP2	0.759762301	1.38188627	6.203527139	1.56E-09
HTRA1	0.760298468	6.128217291	7.187813531	4.02E-12
COL4A4	0.760392264	1.348413625	7.160676808	4.78E-12
CDA	0.760479993	4.17802787	4.27655267	2.45E-05
CBX6	0.760622333	4.175604112	5.928511143	7.34E-09
ARHGEF26	0.760648655	1.984055151	6.240908621	1.26E-09
FILIP1	0.76081774	1.844600921	6.443931114	3.86E-10
NYNRIN	0.761085904	3.487919408	7.159948682	4.80E-12
RUNX1T1	0.761388789	1.59572048	7.496914253	5.43E-13
PLPP1	0.761401922	5.397624167	8.842598802	4.70E-17
NRXN2	0.761422928	1.286080372	7.075639154	8.19E-12
TGFBI	0.761498491	6.257170959	6.078768454	3.17E-09
TPPP3	0.76155992	4.842052514	4.490064609	9.69E-06
IGHA2	0.761939596	7.477104025	2.67636596	0.007793354
SNRPN	0.762061998	3.651340857	6.725124824	7.18E-11
LAMP5	0.762953657	1.701404879	6.238250975	1.28E-09
FGF2	0.762977072	1.482228375	7.470206569	6.47E-13
LDB3	0.763005086	1.301666289	5.349568713	1.60E-07
ACTA2-AS1	0.763040806	1.746805951	6.621956444	1.34E-10
KLF15	0.763866265	1.652036126	6.445214604	3.84E-10
DOK5	0.763926192	1.571680938	9.998860391	7.25E-21
PRKG1	0.764004301	2.157237222	8.348280572	1.64E-15
PSD	0.764664529	2.090084061	5.226916092	2.97E-07
ASAP3	0.765465971	2.960405116	8.244383901	3.40E-15

PID1	0.766529404	2.89241266	5.751179005	1.94E-08
SFTA2	0.766843064	3.946426981	3.298533787	0.001071921
SLC2A10	0.76684387	3.774407319	6.100505078	2.81E-09
SCARA5	0.76702427	1.490358583	5.954549979	6.36E-09
CHRNA3	0.768101798	1.241087759	5.321485878	1.84E-07
COL4A2	0.768144359	7.484418455	7.320118053	1.72E-12
PKIA	0.769258016	1.466472553	8.169401503	5.74E-15
PRKAA2	0.769449909	1.138027254	6.778850392	5.17E-11
MAPK8IP1	0.76970562	2.524628351	6.495530501	2.85E-10
PALLD	0.770031791	6.042266592	6.032651374	4.11E-09
SYT11	0.770064663	3.102392766	7.009114727	1.24E-11
CHRM2	0.770348472	0.922369984	5.422009582	1.10E-07
RBP7	0.770483922	2.187008836	7.989510594	1.99E-14
WNT9A	0.772203619	1.545568884	6.724604646	7.20E-11
EGR3	0.772548152	2.599295055	6.414536369	4.59E-10
A4GALT	0.772909717	3.70579699	7.209260034	3.50E-12
TSPY26P	0.773100932	1.843743725	8.13742672	7.17E-15
CD93	0.773198304	4.61781765	7.693960426	1.47E-13
MAGED1	0.773490813	5.696013294	7.886729624	4.01E-14
LOX	0.773497156	3.897018987	6.671216401	9.95E-11
CLIP4	0.7737502	2.733383561	7.292583549	2.05E-12
MID2	0.773896768	2.459472552	7.739342629	1.09E-13
PLPP7	0.773940284	1.493800609	9.544347278	2.46E-19
SRPX2	0.774753498	3.292745281	7.281753258	2.20E-12
TCEAL3	0.774757174	4.191165448	7.874822307	4.35E-14
ANK2	0.775027966	1.405697811	7.048609252	9.71E-12
GPX7	0.775127917	3.730846984	7.316632721	1.76E-12
CYGB	0.775233041	4.917351012	8.734070025	1.04E-16
FCER1A	0.775320996	1.367479277	6.253129442	1.17E-09
CACNA1C	0.775896676	2.009079857	6.304362199	8.74E-10
LAYN	0.777800054	2.50399021	7.558751167	3.62E-13
GFRA3	0.778115719	1.440727807	5.814908763	1.37E-08
SULT1C4	0.778348056	1.488800099	7.836032486	5.66E-14
PHLDB1	0.778483081	4.169048735	8.55150822	3.86E-16
PRSS23	0.778851231	5.006089767	7.710234796	1.32E-13
NRSN2	0.779917891	4.584558723	7.153702385	4.99E-12
MEG3	0.780261197	2.270719579	7.262262518	2.50E-12
STOM	0.780906918	6.657936935	8.603455904	2.66E-16
CLIC4	0.781567791	6.221005861	7.764045931	9.20E-14
LMO3	0.782355036	1.527762375	7.185384453	4.08E-12
PDGFD	0.782606794	2.645874497	8.03611125	1.44E-14
IL6	0.782610596	2.57813122	4.754732621	2.91E-06
NID2	0.783520413	3.823633081	7.218006995	3.31E-12
ALPK3	0.784003155	2.005625025	6.865663495	3.03E-11
G0S2	0.784512587	4.642233279	5.527949093	6.35E-08
HAND2-AS1	0.784801889	1.13595697	5.159786119	4.15E-07
ATP2B4	0.784988816	5.092269715	6.767419196	5.55E-11
BNC2	0.786219063	1.503076073	7.883624746	4.10E-14
NLGN2	0.7863046	3.45966421	7.665490338	1.78E-13
PNMA8A	0.787553353	1.207226234	7.37411457	1.21E-12
EBF4	0.78812219	2.993859681	6.770177367	5.46E-11
KCNH2	0.788930938	2.75850119	4.726087804	3.32E-06
LRRC17	0.788944714	1.774384938	8.272588844	2.79E-15

MARVELD1	0.789030211	5.124162525	7.834976378	5.70E-14
DYNC1I1	0.789076858	1.427799088	7.813680195	6.58E-14
KRT17	0.789387855	4.913859482	2.689395264	0.007501589
GYPC	0.78944798	4.20248639	6.736793619	6.69E-11
PER1	0.789460518	4.491836833	7.021518596	1.15E-11
BST1	0.789703552	2.58942287	8.127428507	7.68E-15
FMO2	0.790382372	2.022187552	5.578858016	4.86E-08
RBMS1	0.790474428	4.714494944	8.850025833	4.45E-17
CACNA2D1	0.790821206	2.314210647	6.653927419	1.10E-10
CAND2	0.791375539	1.425228206	7.631364766	2.23E-13
RTL5	0.791439252	1.619832811	8.712999061	1.21E-16
PHYHD1	0.791980868	2.319470823	6.668137273	1.01E-10
GC	0.792293157	1.465762006	3.877608921	0.00012603
ZBTB16	0.792799934	1.251903673	6.701831169	8.27E-11
GABARAPL1	0.793039184	4.305835321	9.127002124	5.75E-18
GPR27	0.794215775	1.216309306	6.594997352	1.57E-10
HOXA4	0.797420127	1.96272261	7.77339358	8.64E-14
ADAMTSL3	0.797824147	1.36134832	7.968843591	2.29E-14
VGLL1	0.798411124	1.447204497	4.349618791	1.79E-05
ADAMTSL4	0.798823867	3.192509677	6.932172028	2.01E-11
CTSE	0.798901109	7.282438751	2.617128237	0.009252716
CDO1	0.798953342	1.126227036	8.433525098	8.96E-16
SORBS2	0.799278634	3.3534836	6.069740469	3.34E-09
SLC2A3	0.799626178	4.088182925	6.960374832	1.69E-11
F2RL3	0.79974701	2.81939169	7.578012374	3.18E-13
AP001189.3	0.799906303	1.482552118	9.575050247	1.94E-19
SH3PXD2B	0.799983053	4.163914137	8.712239039	1.21E-16
COL5A2	0.800243944	5.778481407	6.566250095	1.87E-10
SHF	0.800917616	2.107874394	8.030251475	1.50E-14
SCN4B	0.801235171	1.305319013	9.165070724	4.33E-18
LPAR1	0.801556941	2.999755298	7.544181196	3.98E-13
AADAC	0.801669714	2.650463595	4.202530031	3.36E-05
LGI2	0.801862572	1.877004141	6.975735748	1.53E-11
FAT4	0.802171049	1.707718874	8.779478295	7.44E-17
ACTC1	0.802221108	1.564785862	6.672855314	9.86E-11
MRAS	0.802390583	3.003237214	8.267671594	2.89E-15
PLAT	0.803149593	5.243487836	9.202958346	3.26E-18
PTN	0.80322722	3.049704674	6.342470534	7.00E-10
TWIST1	0.804040103	2.270943708	6.916014035	2.22E-11
FAM107A	0.804982222	2.121464079	6.906975051	2.35E-11
CMTM3	0.805019754	4.472513472	8.169149227	5.75E-15
FGF10	0.805611881	1.308886656	6.505088194	2.69E-10
SH3RF3	0.805802522	2.510765107	9.099341583	7.06E-18
ADRA2A	0.806022635	3.482584278	3.898206062	0.000116189
ANTXR2	0.806907593	4.06273451	7.088589005	7.54E-12
NEXN	0.806946251	3.984062266	5.216304152	3.13E-07
CXCL12	0.8070091	4.182262968	5.837186238	1.21E-08
GLIS3	0.807133053	2.514311368	7.215871617	3.36E-12
FOXP2	0.807663214	1.379489498	6.881761667	2.75E-11
HIC1	0.807729765	2.973505381	8.178300783	5.40E-15
BVES	0.808571632	1.666326961	6.813865714	4.17E-11
PLAGL1	0.808782074	2.793864884	7.914123755	3.33E-14
DST	0.808863235	3.77641501	7.262867055	2.49E-12

VIP	0.80931957	1.218951316	4.936282175	1.24E-06
ARHGAP23	0.809631802	4.237882	7.206213713	3.57E-12
EPHA7	0.809631869	1.103674978	6.201438754	1.58E-09
VWF	0.810498671	5.199213036	6.946515034	1.84E-11
NPR1	0.810689453	2.095176507	9.086131784	7.79E-18
CRMP1	0.810850047	2.72067001	8.47689923	6.58E-16
BEX1	0.811138822	1.475729178	4.166401273	3.90E-05
CCDC3	0.811390814	4.157552932	9.183213443	3.78E-18
DLC1	0.811602972	3.483702684	8.514530202	5.03E-16
DMD	0.811773371	1.938403825	6.748282453	6.24E-11
THSD4	0.812084126	2.397483604	7.356671959	1.36E-12
DNALI1	0.812638934	2.502800275	6.099083754	2.83E-09
TNFAIP8L3	0.812739943	1.68420032	9.048131895	1.03E-17
GRIK5	0.812813087	1.359597179	7.081695708	7.88E-12
MAPK10	0.81294751	1.556241506	8.800168483	6.40E-17
CSPG4	0.812951092	3.585686087	6.351115499	6.66E-10
KLF9	0.813152919	4.429135023	7.509398046	5.01E-13
DENND5A	0.813837379	3.986460003	8.911824956	2.83E-17
ZSCAN18	0.814068868	2.003936459	7.666922816	1.76E-13
WFDC1	0.814243476	2.611502416	7.562288807	3.53E-13
SPON2	0.815404676	5.990749529	7.376345486	1.19E-12
SYNPO	0.815701641	5.311453867	7.549100387	3.85E-13
PDZD4	0.815773791	1.257007317	7.617147266	2.46E-13
SYNDIG1	0.815978846	2.024670306	7.80523806	6.97E-14
COL12A1	0.816100194	5.348234446	6.060256871	3.52E-09
RHOJ	0.817018045	3.248157097	8.726992006	1.09E-16
DNAJB5	0.818167159	2.46176498	6.679821763	9.45E-11
TYRP1	0.81828681	1.498903665	6.645132405	1.17E-10
ST6GALNAC5	0.818368409	1.944873436	8.14332687	6.88E-15
CSDC2	0.818550109	1.377574939	9.559123066	2.19E-19
SLC16A4	0.818691068	2.644312721	8.042428745	1.38E-14
CLEC11A	0.818996164	3.991539144	7.291937092	2.06E-12
RTL8C	0.819038488	6.802666069	9.647063078	1.12E-19
NREP	0.819279184	4.617726932	8.669720834	1.65E-16
PLOD2	0.819555567	4.786346455	8.569845942	3.39E-16
TPSB2	0.82021221	3.890664539	4.844155365	1.91E-06
RBMS3	0.821103194	1.949607455	8.724283712	1.11E-16
CDKN1C	0.821350975	4.060408619	6.145368851	2.18E-09
OLFML1	0.822535063	2.97187898	8.918919068	2.68E-17
CACNG4	0.822983331	1.411058328	5.328482935	1.78E-07
CNRIP1	0.824276801	3.124330994	9.488676806	3.76E-19
THNSL2	0.82452862	2.454506229	6.239655472	1.27E-09
WLS	0.825230106	5.506044485	8.097175205	9.48E-15
APOA4	0.825623682	1.304599814	3.697556305	0.000252697
EGR1	0.825680663	6.743631375	5.96401785	6.03E-09
SYNGR1	0.825999991	2.267251489	6.635899391	1.23E-10
P3H3	0.826138072	3.422743242	7.870625859	4.48E-14
PDPN	0.826140071	4.263768576	7.677808089	1.64E-13
CD248	0.826252767	5.028713979	7.651871079	1.95E-13
ATP1B2	0.826358373	1.583613597	8.633299465	2.15E-16
DIO2	0.82737972	3.663836065	7.157371938	4.88E-12
CLEC3B	0.827822639	2.834129474	7.167406444	4.58E-12
CGNL1	0.827964314	2.023866535	7.390009495	1.09E-12

NOTCH3	0.828189992	5.650275961	7.941133796	2.77E-14
GHR	0.828600232	1.288969468	9.10678754	6.68E-18
SEMA6A	0.82869372	3.108793279	5.888726346	9.15E-09
LDB2	0.829030795	3.134325203	10.2761471	8.09E-22
ZFPM2	0.829041626	1.614376203	9.150175142	4.83E-18
SORCS2	0.829174573	1.361537269	9.562151468	2.14E-19
ARL4D	0.829184595	2.013821819	6.788442806	4.88E-11
EEF1A2	0.829269771	2.092542697	3.642144623	0.000311329
LINC02532	0.829413197	1.684311488	4.844074321	1.92E-06
SLC15A1	0.829533674	1.627578519	4.910545814	1.40E-06
SERTAD4	0.829694453	2.181194506	6.848454868	3.37E-11
PDE9A	0.829778244	2.079153537	7.319416333	1.73E-12
MEIS3	0.829810913	2.87188451	8.793768255	6.71E-17
RASD2	0.82985975	2.298001863	8.570213904	3.38E-16
CAVIN3	0.830194815	5.970276672	8.188429793	5.03E-15
CDC42EP3	0.8307087	3.8091811	7.520638596	4.65E-13
REEP2	0.831587857	1.855266396	6.654068647	1.10E-10
RAB3IL1	0.832236582	3.383677417	8.545997775	4.02E-16
FNDC4	0.832462626	2.707304486	8.774332655	7.73E-17
IGFBP3	0.832479917	7.347734881	6.973581319	1.55E-11
GPBAR1	0.833202361	2.365296493	7.191217005	3.93E-12
TGFB3	0.833366496	3.321922729	7.006444957	1.26E-11
UPK1B	0.833537669	1.437540515	4.367083524	1.66E-05
TSC22D3	0.834234512	5.964715277	7.115991022	6.34E-12
ITGA5	0.834277539	5.67767904	7.267075413	2.42E-12
NR4A1	0.834506523	5.251510238	5.831704958	1.25E-08
GAS7	0.834924182	3.185772368	7.215990058	3.36E-12
COL4A5	0.835584057	2.346968624	6.255050336	1.16E-09
LINC02381	0.836499776	4.115474117	4.589698903	6.20E-06
RECK	0.837441604	2.26640515	9.949667748	1.07E-20
BHMT2	0.837700461	1.477542537	8.251594184	3.23E-15
GLI1	0.837773304	1.777518467	8.490077985	5.99E-16
SMOC1	0.838434103	1.939237672	4.756228339	2.89E-06
ANKRD65	0.839271655	2.107237885	7.404280789	9.96E-13
APOB	0.840511274	1.020885681	4.88183995	1.60E-06
MMRN1	0.840599663	1.614628151	7.346342702	1.45E-12
HEG1	0.84075444	4.248286951	8.402829519	1.11E-15
TCF7L1	0.841020576	3.182596729	8.40839287	1.07E-15
RAB31	0.841350222	5.130097702	7.825187487	6.09E-14
TTC28	0.842056793	2.443609816	9.596370373	1.65E-19
PMP22	0.842626294	6.059694276	8.301763275	2.27E-15
MEOX2	0.842762224	1.382154233	7.083968085	7.77E-12
CPNE8	0.843411199	2.36796619	10.1457385	2.28E-21
NID1	0.84382359	5.809840366	8.46133956	7.36E-16
AC015922.3	0.844191097	3.086366113	7.698244525	1.43E-13
NRP1	0.844400747	4.77722118	9.872307213	1.95E-20
PRKD1	0.845428194	1.705419041	10.50452799	1.30E-22
FOLR2	0.84565555	3.582949892	5.808348466	1.42E-08
SMARCD3	0.845838348	3.180036207	9.1931453	3.51E-18
C4B	0.846501151	2.537139014	6.722631728	7.29E-11
S1PR1	0.847226177	4.274004854	8.081332825	1.06E-14
NTN4	0.847583146	3.921193307	8.535806574	4.32E-16
RCN3	0.847896034	5.283198974	7.870215548	4.49E-14

RARRES2	0.84791595	5.354023083	7.955812519	2.51E-14
INHBA	0.848453289	4.129517301	6.278828627	1.01E-09
COL16A1	0.848704108	4.085218475	7.485455243	5.86E-13
TSHZ3	0.84924096	2.43054332	8.653885105	1.85E-16
FAM110B	0.849453646	1.783069639	9.010236152	1.37E-17
HTRA3	0.849850817	5.334290751	6.193629916	1.65E-09
GASK1A	0.851167965	1.383550872	8.635871373	2.11E-16
DTNA	0.851423304	1.509724012	7.447646587	7.50E-13
MMRN2	0.852247868	3.781587517	9.291433126	1.68E-18
KCNJ8	0.852248901	2.861210038	8.818337923	5.61E-17
PCDHB5	0.852356465	1.149818435	8.257338286	3.11E-15
SCG5	0.85378574	2.59336966	8.450135798	7.97E-16
MAMDC2	0.853872231	1.44456942	6.395228917	5.14E-10
PRICKLE2	0.854549521	2.044180566	7.828790916	5.94E-14
PCOLCE	0.855204754	5.887806547	7.336948946	1.54E-12
MYOC	0.855498021	0.97976891	5.802359145	1.47E-08
EPHA3	0.855987904	2.158224415	6.664657247	1.04E-10
PDLIM7	0.856219257	6.575982384	7.921077165	3.18E-14
GALNT15	0.856219639	1.388921516	9.392189184	7.83E-19
SPART	0.85630669	3.110540311	7.99558009	1.91E-14
RGS4	0.85655369	1.99297759	7.207750158	3.54E-12
LSAMP	0.857287882	1.953611427	8.528458228	4.56E-16
PPP1R1A	0.857995966	1.266525261	6.244141064	1.24E-09
TFPI	0.858349593	3.651546761	8.043373228	1.37E-14
PTPRM	0.858404515	3.622149463	9.235854991	2.55E-18
LBH	0.858925733	5.305962947	8.917314902	2.71E-17
S1PR3	0.859595687	2.903395538	7.609303993	2.59E-13
FILIP1L	0.859601368	5.31369653	7.695084477	1.46E-13
FAXDC2	0.860439315	2.930876943	7.072821556	8.33E-12
MAP6	0.860686804	1.47732905	8.758168798	8.69E-17
MAGEH1	0.861088095	3.537680465	9.538795275	2.56E-19
PDE2A	0.861988512	2.150543933	8.793508875	6.72E-17
LINC01436	0.862198409	1.409524333	6.953500535	1.76E-11
MMP11	0.862801967	4.857653806	4.596592003	6.01E-06
ZNF423	0.863939115	1.812703386	8.930245324	2.47E-17
CARMN	0.86434245	1.610402743	6.453886211	3.64E-10
PLTP	0.86439273	6.342558959	7.178686734	4.26E-12
SMAD9	0.865341256	2.168925382	6.705163399	8.10E-11
AP000892.4	0.865759668	1.768032254	6.676393633	9.65E-11
NRXN3	0.865840316	1.462833344	6.61364821	1.41E-10
MCAM	0.866659538	5.697568545	7.980862765	2.11E-14
SCRN1	0.866698875	4.713419848	7.100800268	6.98E-12
SHISA2	0.86734346	2.025766167	7.303238267	1.92E-12
STEAP4	0.867493018	2.011038527	7.358296947	1.34E-12
CPXM1	0.867526426	3.591269144	6.550930553	2.05E-10
C1QTNF7	0.867728192	1.213581334	8.613985146	2.47E-16
HSPA2	0.86795495	2.51605581	8.188004433	5.04E-15
ZNF521	0.868048139	2.247667671	9.948608041	1.07E-20
ITGA9	0.868593018	2.706289044	7.418260927	9.09E-13
SETBP1	0.870020146	1.990801874	8.042191719	1.39E-14
NRP2	0.870030754	4.091812655	7.069732292	8.50E-12
OSMR	0.870131252	4.897089106	7.855233218	4.97E-14
LOXL1	0.870806105	4.792208581	8.286064981	2.54E-15

LAMA4	0.870862321	4.942437278	8.937607358	2.34E-17
MRC2	0.871784805	4.731954403	7.473528718	6.33E-13
NIBAN1	0.872562784	3.989879858	5.853232738	1.11E-08
FOXF2	0.872962778	3.816815152	6.721138355	7.36E-11
RBPMS	0.873866018	5.754907649	8.399238495	1.14E-15
SNAP25	0.875128541	1.348941262	6.213487703	1.47E-09
CHRD	0.875225127	1.995879335	9.133431511	5.48E-18
DCHS1	0.87530194	3.185351085	8.757691787	8.72E-17
PAPLN	0.875554206	2.745663574	7.750415691	1.01E-13
NR0B2	0.875659226	3.59125081	4.541649229	7.70E-06
NXN	0.876647419	4.383967895	7.493383258	5.56E-13
PARM1	0.876791593	3.723652886	6.481988825	3.09E-10
KIT	0.877312751	2.33140474	7.473485061	6.34E-13
PPP1R12B	0.87732313	3.999563117	5.287478835	2.19E-07
TCEAL7	0.877549476	1.751915	9.739982964	5.45E-20
CAP2	0.878764281	2.749474459	7.047634317	9.77E-12
FABP3	0.879469862	4.076774583	6.829753939	3.79E-11
LYNX1	0.87966179	2.381333823	5.333692359	1.73E-07
COL1A1	0.880676233	9.794186941	5.618744045	3.94E-08
GAMT	0.88109095	2.933872252	6.342018065	7.02E-10
LOXL4	0.881842227	1.598245704	7.926765596	3.06E-14
TSPYL5	0.882199484	2.357096644	6.625000965	1.32E-10
ITGA11	0.8825801	3.008706731	7.141789809	5.39E-12
KANK2	0.882663395	5.014238505	7.848852081	5.19E-14
KCNK3	0.882840756	1.197710752	7.703056668	1.39E-13
NTM	0.88318122	2.293348832	7.83343155	5.76E-14
KCNS3	0.883263064	3.045027424	8.046260799	1.35E-14
PRRX1	0.883580244	3.685587429	6.21797453	1.44E-09
ALDH1A3	0.884138461	3.283905911	7.581055161	3.12E-13
FRMD6	0.884962332	3.171262395	8.147006578	6.71E-15
SALL2	0.88811227	1.500055672	8.502810407	5.47E-16
LAMC1	0.888330501	5.989026766	9.320080353	1.35E-18
CLDN5	0.888424037	3.50895092	7.58318062	3.08E-13
GNAI1	0.889232125	3.302906861	8.986197591	1.63E-17
TMEM100	0.889257057	1.518995358	6.999296789	1.32E-11
DKK3	0.889412585	5.778669559	8.738314809	1.00E-16
ACKR3	0.890357769	4.121285234	9.893313572	1.66E-20
ETNK2	0.890616401	2.027170068	7.256952808	2.58E-12
SYNC	0.891040089	1.648458753	7.448621472	7.46E-13
GALNT16	0.891401608	1.197108116	9.046620508	1.04E-17
C3orf70	0.891963473	2.169145652	6.892674589	2.57E-11
NFATC4	0.89533211	2.45640104	9.97427501	8.79E-21
HMCN1	0.895469417	1.558746105	9.280670883	1.82E-18
GPX8	0.896397881	3.605101622	9.377753243	8.73E-19
L1CAM	0.896407221	2.306854219	4.871612712	1.68E-06
C4A	0.897449899	2.567973888	7.215049291	3.38E-12
CXCR4	0.897936618	6.034520818	7.256818385	2.58E-12
HIF3A	0.897953288	1.874450811	5.551651915	5.61E-08
FOLR1	0.898458129	2.501710185	4.04179307	6.53E-05
NFASC	0.898604877	2.230557162	6.657533773	1.08E-10
PDE1A	0.899160478	1.860715721	10.40461781	2.90E-22
FIBIN	0.899447974	2.851348536	7.783945633	8.05E-14
ARMCX1	0.900539532	2.639640145	9.354090903	1.04E-18

LRP1	0.900602761	5.908559504	8.695487205	1.37E-16
TUBA1A	0.901408	6.584563758	7.593739187	2.87E-13
PNCK	0.901650713	1.466270881	5.948461007	6.57E-09
GASK1B	0.901709749	4.635375879	8.789634044	6.91E-17
FADS2	0.902080475	3.991400744	6.859586877	3.15E-11
SYDE1	0.902388182	3.472818453	9.993387765	7.56E-21
RGS2	0.902607774	5.62157997	7.551634267	3.79E-13
HSPG2	0.903222539	6.519620722	7.4966062	5.44E-13
AKT3	0.905864096	3.060422662	8.593165976	2.87E-16
FBXL7	0.906452974	2.634183118	9.649610842	1.09E-19
CLIC6	0.907505698	2.941234182	5.193309879	3.52E-07
EPHX3	0.907771747	2.213542528	5.240399042	2.78E-07
ZEB1	0.908737744	3.882383908	8.125244179	7.80E-15
PROS1	0.908889849	3.775531652	7.959420297	2.44E-14
THBD	0.910937687	4.189698846	9.215621604	2.96E-18
CRABP1	0.911514114	1.644175738	4.503766161	9.11E-06
LTF	0.912286966	3.721826236	3.289261472	0.001106758
ITGA8	0.912301651	2.219060505	7.174624738	4.37E-12
PCDH7	0.912434817	3.623831161	5.823502683	1.31E-08
LINC00578	0.912810058	2.104636825	7.084111131	7.76E-12
SMARCA1	0.913603236	3.460105265	8.246128922	3.36E-15
PROC	0.91362275	2.027815292	6.998257774	1.33E-11
SVIL	0.915512432	4.784644117	6.927998796	2.06E-11
FEZ1	0.915646175	3.01667729	9.577012402	1.91E-19
VSTM4	0.917351077	2.302493278	9.060420669	9.43E-18
NR4A3	0.918334883	2.387245756	7.686728203	1.55E-13
ATP1A2	0.919551386	1.241729207	6.23055301	1.34E-09
GPC6	0.920502456	2.87151001	8.248378773	3.31E-15
COL6A2	0.920584801	8.205715124	7.567058395	3.42E-13
THY1	0.920980428	5.914188027	8.092685633	9.78E-15
TPST1	0.922034152	3.559196371	9.975060323	8.73E-21
TSPAN18	0.922160905	3.951454008	7.324740582	1.67E-12
FAM20C	0.922219649	5.228426346	8.500532648	5.56E-16
MFGE8	0.922791472	6.434078342	10.5617339	8.19E-23
SHISA3	0.923017417	1.62059912	6.214510601	1.47E-09
JAM2	0.923327897	2.665233395	7.996808533	1.89E-14
PTGS2	0.924226276	3.126512744	5.806602713	1.43E-08
TMEM200B	0.924408612	1.846730128	10.88271758	6.01E-24
CAV1	0.924515229	5.351102093	7.382367077	1.15E-12
SERPINE2	0.925342321	3.525156281	7.23165078	3.04E-12
RAMP1	0.926357875	5.229818657	5.492752611	7.63E-08
SERPING1	0.926973132	7.752067411	7.129458496	5.83E-12
SCRG1	0.92705816	1.249479099	6.554463698	2.01E-10
SELENOM	0.927411312	5.784696777	8.215209848	4.17E-15
TFF1	0.928319158	8.171654887	2.814836705	0.005156857
ATP8B2	0.928795321	3.334678568	8.457998671	7.53E-16
DAAM2	0.928914161	2.737672325	7.683087832	1.58E-13
RGS5	0.930764123	6.33651257	7.834844141	5.71E-14
CNTN1	0.931276467	1.28858128	7.468691026	6.54E-13
GLT8D2	0.932162106	3.412441855	9.243678237	2.40E-18
LHFPL3-AS2	0.932864363	1.974685637	5.61469834	4.02E-08
SPARC	0.932944241	8.789140904	8.137963687	7.14E-15
EFHD1	0.933198741	2.726037502	9.238184884	2.50E-18

MPDZ	0.934569134	2.193353733	9.121796236	5.97E-18
ZNF853	0.935002139	2.16537138	8.127124078	7.70E-15
SST	0.936360213	2.036846122	3.916584943	0.000108027
TACR2	0.936597245	2.411695885	4.320481178	2.03E-05
MAP1A	0.936755651	2.612564821	8.297199663	2.35E-15
TM4SF4	0.936916768	3.114389185	3.702508661	0.000247997
FAP	0.937220721	3.213650048	6.96685635	1.62E-11
KRT7	0.937803026	5.962383296	3.456756061	0.000614158
MAN1C1	0.937914694	2.994112311	9.530077689	2.74E-19
IGFBP7	0.938201581	9.239714886	9.384956489	8.27E-19
SCG2	0.939021459	1.591176611	7.248358449	2.73E-12
SPEG	0.939183931	1.863117725	6.318980396	8.03E-10
METTL24	0.940109676	1.535139799	7.10708956	6.71E-12
COL5A1	0.94083144	6.254064853	7.145558584	5.26E-12
MXRA7	0.941147324	4.569090512	8.479424013	6.47E-16
MYOCD	0.941189626	1.702006858	6.56143684	1.92E-10
TNS2	0.941757976	4.83645105	9.624782192	1.33E-19
PCDHGC3	0.942907235	2.964398922	8.64525575	1.97E-16
TUBB6	0.943341361	5.316821039	8.856804593	4.23E-17
LTBP3	0.944422859	5.716205827	8.854345215	4.31E-17
DACT1	0.944988021	3.127454291	8.078188158	1.08E-14
EFS	0.945493496	2.104735256	9.960275583	9.81E-21
TSPAN2	0.946135914	2.879921885	6.964287255	1.65E-11
PDLIM4	0.946680634	3.905989059	7.978560003	2.14E-14
SIX2	0.948158822	2.313047213	5.01216624	8.57E-07
CTSK	0.948177925	6.152347361	8.400274035	1.13E-15
F10	0.948363182	2.483236614	6.283269549	9.87E-10
CHST3	0.948769962	2.714959976	8.816080497	5.70E-17
GUCY1B1	0.950127242	3.878418591	8.79449694	6.67E-17
SSPN	0.950511273	3.432152316	7.941675293	2.76E-14
C1R	0.950576843	6.594610471	7.91729091	3.26E-14
RASL12	0.950848083	3.123229387	9.868477618	2.01E-20
CAV2	0.951014853	4.81740326	8.127633075	7.67E-15
SMIM24	0.951099258	5.853839474	4.356658801	1.74E-05
AXL	0.951198936	4.622861126	8.951791954	2.11E-17
EDNRA	0.952175114	3.384892488	9.11963154	6.07E-18
ADGRL2	0.952310619	3.518891552	9.490435822	3.71E-19
PRIMA1	0.952357169	1.622641508	6.251646937	1.18E-09
RNF150	0.953367338	1.730041862	7.75623349	9.70E-14
SDC2	0.954055435	4.604539514	10.15218557	2.16E-21
OLFML2B	0.954222658	4.514925902	6.891245589	2.59E-11
CCN2	0.955097589	7.302180429	8.095495778	9.59E-15
BCAM	0.955164513	5.501900114	7.246848592	2.76E-12
SGCD	0.955452427	2.120381096	8.467249227	7.05E-16
DPT	0.956610061	2.597775774	5.869075148	1.02E-08
LARP6	0.957451933	2.000331293	10.18922737	1.61E-21
PDGFRB	0.957452796	5.868292145	8.482220104	6.34E-16
CERCAM	0.957491101	4.080055131	8.366193951	1.44E-15
COL6A3	0.95795085	6.846166773	7.212144671	3.44E-12
IL33	0.960339122	3.571872467	5.80495694	1.45E-08
COLEC12	0.960496298	2.062846171	8.663672327	1.72E-16
NPW	0.960781722	2.889051386	4.857446832	1.80E-06
PFN2	0.96094868	5.04681165	6.529122231	2.33E-10

TUSC3	0.961134067	2.650115111	9.018578221	1.29E-17
SHISA4	0.961148684	3.585687858	9.100533525	7.00E-18
CRLF1	0.961647402	1.758873327	7.722001828	1.22E-13
QPRT	0.96193816	3.428179511	5.111422882	5.28E-07
AC104083.1	0.962373128	3.547094161	7.572684639	3.30E-13
AMIGO2	0.963460759	3.206771715	8.073076137	1.12E-14
STC1	0.963687498	4.307007011	9.099058908	7.08E-18
VASN	0.963880848	4.141726997	10.36670906	3.93E-22
PTGER3	0.964242664	1.529200591	9.87379556	1.93E-20
PRSS2	0.964456658	3.80242508	2.985051757	0.003035145
BMERB1	0.965030464	3.469907308	9.03088177	1.17E-17
LINC00261	0.966219184	2.216744975	5.065479208	6.61E-07
CCL19	0.966960452	3.760788502	4.191032512	3.52E-05
WASF3	0.968701954	1.834148428	8.36603048	1.45E-15
GJA1	0.969529571	4.906928098	8.980819127	1.70E-17
TNC	0.969863252	5.131638422	5.545346334	5.80E-08
SVEP1	0.970041125	1.918502846	8.519822502	4.85E-16
F5	0.970089	3.114054275	5.329047663	1.77E-07
ARHGEF17	0.970837188	3.875612017	10.66175242	3.64E-23
ANGPTL2	0.971296008	5.219647542	7.662659265	1.81E-13
HOPX	0.972272956	3.69581099	6.814222134	4.17E-11
AMOTL1	0.972343761	3.516693826	8.390016984	1.22E-15
IL1R1	0.972559574	4.888118598	10.07634642	3.94E-21
KCNE4	0.972938922	2.494641811	8.925934864	2.55E-17
PTGDS	0.973238523	5.679899229	5.197013043	3.45E-07
SMIM10	0.973318111	2.37588293	10.36297826	4.05E-22
TMTC1	0.97364846	2.347056619	8.405919827	1.09E-15
MYH10	0.975483368	3.69152503	8.51603143	4.98E-16
ZNF667-AS1	0.975826307	2.439285214	8.958896792	2.00E-17
COL1A2	0.976315865	8.986731325	6.796244122	4.65E-11
CASQ2	0.976454231	1.419426538	6.39094754	5.28E-10
ANGPTL1	0.97707595	1.65995319	6.229799897	1.34E-09
SHC2	0.97870959	3.518967792	8.805015087	6.18E-17
LRRN4CL	0.980116809	1.82046225	10.23441595	1.13E-21
GGT5	0.980222661	4.279175	8.744072113	9.63E-17
RTL8B	0.980442115	3.31170441	10.50605796	1.28E-22
KIRREL1	0.980484522	3.758421694	10.42747046	2.41E-22
PLXDC2	0.981774072	3.453166242	8.29190639	2.44E-15
SELP	0.983879031	2.502869851	7.247840611	2.74E-12
ST3GAL4	0.984368322	4.116381502	8.521000473	4.81E-16
ABCA8	0.984627542	1.663361789	7.340335547	1.51E-12
C8orf88	0.984685096	1.529356271	7.706589825	1.35E-13
FAM180A	0.985872462	1.473920988	9.611364458	1.47E-19
ZNF880	0.985977952	2.324483281	9.023793213	1.24E-17
MATN2	0.986828382	4.008957298	6.3663267	6.09E-10
CLDN11	0.987912723	1.934530103	8.517847554	4.92E-16
SLCO2A1	0.988497818	4.541323893	8.28103387	2.63E-15
NGFR	0.988680067	1.891073257	5.809840141	1.41E-08
RRAD	0.989039706	2.947663945	6.998685536	1.33E-11
FBXO32	0.989320454	4.207071908	7.572238253	3.31E-13
HPN	0.990353512	1.823265884	5.228464785	2.95E-07
SAMD11	0.991704078	2.129608088	10.12087256	2.77E-21
PDGFRA	0.992418531	4.077719504	8.562409343	3.57E-16

CLMP	0.992815657	2.92729475	7.257843389	2.57E-12
SLC16A2	0.993300504	3.203402325	7.70970075	1.32E-13
FSTL3	0.993683358	4.576549803	7.511212893	4.95E-13
GFPT2	0.994998308	2.70704427	8.374078671	1.37E-15
CILP	0.995104734	1.827475039	6.119550858	2.52E-09
EVC	0.998527162	2.605979588	9.0599647	9.46E-18
ADAMTS8	0.998833963	1.567575813	7.805219748	6.97E-14
AOX1	1.001368376	1.391429171	9.498371845	3.49E-19
CNTNAP1	1.001559674	2.824475977	9.566554584	2.07E-19
CTF1	1.001969433	2.754748844	7.563065494	3.51E-13
COL8A2	1.002343345	2.609423964	8.992422021	1.56E-17
FKBP10	1.003296085	5.78291969	6.787996155	4.89E-11
LIMS2	1.003425722	3.396970704	7.440187599	7.88E-13
BEX4	1.00416172	3.823437413	8.591506314	2.90E-16
LHFPL6	1.005058854	4.616332831	9.315658584	1.40E-18
RAI2	1.005332131	3.676863779	7.903000867	3.59E-14
CRABP2	1.006189457	4.086104569	4.910158665	1.40E-06
PI16	1.006957503	1.696073803	6.052065418	3.69E-09
ADGRA2	1.007174346	4.333999452	9.096654996	7.20E-18
MLF1	1.00941867	2.159703175	7.687817922	1.53E-13
ADGRD1	1.010788571	1.621322296	9.012992749	1.34E-17
TPSAB1	1.011270831	4.360720974	6.422780869	4.38E-10
CDH11	1.011276138	4.423796399	8.457451852	7.56E-16
GLIS2	1.014390181	3.677506312	10.2316469	1.15E-21
H19	1.014815763	4.445490922	4.142808277	4.31E-05
CPED1	1.014820199	2.941335099	7.040704865	1.02E-11
PTGER1	1.014923398	1.956131253	8.43976336	8.58E-16
KCNMB1	1.019441987	2.540699336	6.61013496	1.44E-10
PHLDB2	1.020264085	3.090155824	8.584441685	3.05E-16
ITGA7	1.020801326	3.530445815	7.639819467	2.11E-13
EML1	1.021660429	3.144468537	8.696356161	1.36E-16
CRISPLD2	1.021984768	5.076586784	9.394388561	7.70E-19
AC015922.2	1.022344473	3.219441371	8.7574221	8.74E-17
EMILIN1	1.023429474	5.702155739	7.879966925	4.20E-14
C16orf89	1.023878667	1.78059303	7.1460179	5.24E-12
HABP2	1.024236597	3.177030146	4.755300296	2.90E-06
TMEM119	1.024766969	4.008553167	7.959869454	2.44E-14
BICC1	1.025426744	2.716726317	9.438645594	5.50E-19
NPTXR	1.026324961	2.093892861	7.722333825	1.22E-13
TWIST2	1.02925662	2.36011618	8.744327355	9.61E-17
CAVIN2	1.029664742	3.328930524	7.434006376	8.20E-13
TAFA5	1.029794033	2.402562844	9.351510824	1.06E-18
ITIH5	1.03057478	2.879357062	8.116168916	8.31E-15
CCN1	1.032479792	6.114272411	7.707442316	1.35E-13
STON1	1.032604001	2.317696392	8.75767202	8.72E-17
MAP1B	1.032972931	3.381025695	8.200585561	4.62E-15
CHGA	1.033222292	2.365306565	3.986288619	8.17E-05
COPZ2	1.033250896	3.832644032	9.577316587	1.91E-19
PLSCR4	1.035157351	3.559064196	9.798939016	3.45E-20
CAVIN1	1.035312288	6.396361072	8.79614873	6.59E-17
SULF1	1.036508232	5.874630554	6.254136097	1.17E-09
IGF2	1.037370749	4.715993293	4.484418643	9.93E-06
CCDC8	1.03745374	1.921316565	8.97741573	1.74E-17

CCL21	1.037513513	5.314727968	4.643822149	4.85E-06
NNMT	1.037875595	5.487407063	8.229285965	3.78E-15
TMOD1	1.038362374	1.860984545	8.266642174	2.91E-15
TMEM47	1.038591212	3.797175964	9.079009217	8.21E-18
TIMP3	1.040095931	2.980585651	8.05979965	1.23E-14
SERTAD4-AS1	1.040291999	2.728409825	7.901632915	3.63E-14
TGFB1I1	1.040656789	4.389617038	9.28778075	1.72E-18
LAMA2	1.040997022	2.35910777	9.260953638	2.11E-18
LRRC32	1.042298147	4.624085381	9.297612048	1.60E-18
JAM3	1.043427096	3.664378987	10.05479531	4.67E-21
SERPINA4	1.043604729	2.862241869	5.125692226	4.92E-07
CLIP3	1.045033656	3.738927833	8.312775391	2.11E-15
FSTL1	1.045095147	6.363177181	9.717307502	6.49E-20
C14orf132	1.0450986	2.074370602	8.975700575	1.77E-17
C1S	1.045207274	7.017838488	8.313320883	2.10E-15
GFRA1	1.045689149	1.550939016	7.976089624	2.18E-14
IGFBP6	1.045991755	4.544528943	8.934442898	2.39E-17
MEIS3P1	1.046606676	2.787709027	9.36880803	9.34E-19
ABCC9	1.046645916	2.117070569	8.584500131	3.05E-16
CH25H	1.047630694	2.311566371	9.467484294	4.42E-19
NTN1	1.049058264	2.942033393	7.285689318	2.15E-12
APBB1	1.049155401	3.301436459	9.426585053	6.03E-19
RASD1	1.049766678	2.977696397	7.282693054	2.19E-12
FOXF1	1.050275142	3.941289991	8.564000554	3.53E-16
OMD	1.050333393	1.925595644	6.978507347	1.51E-11
EFEMP2	1.050558178	4.886607499	10.1177136	2.84E-21
VCAN	1.052977457	4.783115002	8.235087782	3.63E-15
OLFML3	1.055394475	4.607812359	8.44255359	8.41E-16
COL3A1	1.056431578	9.206853803	7.254649298	2.62E-12
PLIN4	1.057858762	1.800189048	5.855050997	1.10E-08
BGN	1.05812447	8.472832125	7.905040868	3.54E-14
ARMCX2	1.058415275	3.134445396	10.05051728	4.83E-21
DUSP1	1.058486432	7.336030662	8.733153704	1.04E-16
CLDN6	1.059040764	1.170847562	4.99266616	9.42E-07
IGFBP4	1.059107943	8.389095697	8.443232218	8.37E-16
PPP1R3C	1.059373834	2.13281746	7.889743346	3.93E-14
RCAN2	1.059871713	3.51982576	8.663561342	1.73E-16
ADAMTS1	1.061919001	4.048036357	9.001658988	1.46E-17
CCN3	1.061925551	2.50318911	8.770027555	7.97E-17
FGFR1	1.062462738	4.020435406	8.527654695	4.58E-16
FLNA	1.062711725	8.166525103	6.674357923	9.77E-11
CTSF	1.062764988	4.146078834	8.55151515	3.86E-16
RASSF8	1.06442983	2.548110755	9.632588135	1.25E-19
REG4	1.069786853	6.349396587	3.070570757	0.002303924
SLC24A3	1.069987543	2.822645795	9.861208985	2.13E-20
LTBP1	1.070675835	4.773700376	8.626760506	2.25E-16
CRISPLD1	1.073877425	2.081284475	9.926753781	1.27E-20
SLIT2	1.0744403	1.92401033	8.348836308	1.63E-15
FABP4	1.075660198	2.14300725	6.316041829	8.16E-10
FENDRR	1.075988555	3.178558318	7.734768159	1.12E-13
ARHGEF25	1.076831588	2.77204779	8.71418178	1.20E-16
MAOB	1.077410431	3.852558341	6.399804519	5.01E-10
SELENOP	1.078342912	4.704217289	7.589296186	2.95E-13

SMO	1.079004054	2.879276123	9.526838952	2.81E-19
IGFBP5	1.079616601	7.112460744	7.749605126	1.01E-13
EHD2	1.080859489	6.178315717	9.882850107	1.80E-20
MMP7	1.083417232	5.516417506	3.865028176	0.000132423
FMOD	1.083983683	5.722314503	8.311310312	2.13E-15
GAS1	1.084724283	2.951292276	6.831682137	3.74E-11
CTHRC1	1.085765346	5.603125499	7.451201833	7.33E-13
SORBS1	1.087458729	4.434177992	6.187360458	1.71E-09
PDGFRL	1.087604745	2.156341936	9.732669123	5.77E-20
LUM	1.091118749	7.895870225	8.313806502	2.09E-15
C3	1.091165598	7.431804069	5.881411393	9.52E-09
SGCE	1.091895081	3.848219839	9.540227077	2.53E-19
HEYL	1.093356053	3.906564474	9.535728227	2.62E-19
A2M	1.093964023	7.999163289	9.444146598	5.27E-19
PDZRN3	1.095726447	3.353357814	8.089337658	1.00E-14
SOD3	1.097384777	6.439783949	7.663760664	1.80E-13
WWTR1	1.100435259	5.088537738	10.11927886	2.81E-21
CES1	1.101363867	2.750375435	5.703189311	2.51E-08
GREM2	1.103945768	1.732761223	7.600140841	2.75E-13
ITGBL1	1.105133152	2.021177321	8.090891199	9.90E-15
GXYLT2	1.105982234	2.400290168	9.37799968	8.71E-19
CALD1	1.106203589	7.574185108	7.796256136	7.41E-14
SERPINE1	1.106740264	5.518095995	7.299536351	1.96E-12
FRZB	1.108225206	3.504831197	7.963625101	2.38E-14
OBSL1	1.108752623	3.208340768	8.208280903	4.38E-15
ZCCHC24	1.108837808	3.747227041	8.922321491	2.62E-17
PDE3A	1.113581875	2.464002269	9.4110606	6.78E-19
AHNAK2	1.11400282	2.954312705	6.79845677	4.59E-11
INMT	1.114774097	2.528058357	8.852600103	4.36E-17
SCARF2	1.114921402	3.872866524	9.318499284	1.37E-18
VTN	1.115703818	1.854423244	6.158383043	2.02E-09
TCEAL2	1.119277362	1.671691049	6.413568171	4.62E-10
GEM	1.120491084	5.077248272	9.770872117	4.29E-20
ISM1	1.122215577	1.931426594	8.469926473	6.92E-16
FBN1	1.122216317	4.422429826	8.543043004	4.11E-16
KCNMA1	1.122239096	2.318423562	6.378975506	5.66E-10
FERMT2	1.122389891	3.962761993	9.274023516	1.91E-18
GUCY1A1	1.122805296	3.772016915	8.092552477	9.78E-15
GSTA1	1.123717796	3.833539991	4.356353503	1.74E-05
PRUNE2	1.124985296	2.630034519	5.768976707	1.76E-08
JPH2	1.126810654	2.301689904	6.689637977	8.90E-11
GNAO1	1.128695761	2.183828991	7.324150734	1.67E-12
CPA3	1.133629174	3.629990479	7.27386817	2.32E-12
GPX3	1.134268782	5.705176353	8.929245915	2.49E-17
DDR2	1.135507244	3.242953893	8.89249526	3.26E-17
ABI3BP	1.137490895	2.965888996	6.948854039	1.81E-11
ASPN	1.137607009	5.020334855	6.798616539	4.58E-11
BEX2	1.138224255	1.872237679	7.032197226	1.08E-11
FOSB	1.138937748	4.31685137	6.088097495	3.01E-09
TTR	1.139592767	1.932813668	5.240562414	2.78E-07
NR2F1	1.14015153	3.716429945	9.136477642	5.35E-18
ADAM33	1.141986944	1.972114699	8.383419618	1.28E-15
LDOC1	1.142838461	3.680750165	8.95763044	2.02E-17

TNXB	1.14385219	2.486160155	7.879830216	4.21E-14
MATN3	1.145522976	2.191825928	9.140803535	5.18E-18
FBXO17	1.146911472	2.44716811	9.425613438	6.07E-19
HAND2	1.150720657	2.071252201	5.633687719	3.63E-08
NDN	1.151299051	3.711162721	9.956065881	1.01E-20
SSC5D	1.155513486	3.208594773	7.782655887	8.12E-14
MN1	1.155964243	2.473423452	8.794120956	6.69E-17
FXYD6	1.157244203	3.658475397	8.647795387	1.93E-16
BEX3	1.157271426	5.683367406	8.853891912	4.32E-17
C11orf96	1.157773562	5.750786891	9.415450303	6.56E-19
UCHL1	1.160913247	2.981128976	7.299737044	1.96E-12
CACNA1H	1.162038206	3.300746582	8.231115334	3.73E-15
FN1	1.164206736	7.873318125	6.871055558	2.94E-11
CAPN6	1.165464472	3.037928497	5.641645356	3.48E-08
TIMP2	1.167554481	7.199037899	10.96886434	2.96E-24
SERPINA1	1.173695744	7.275138442	5.704627253	2.49E-08
SLIT3	1.175033756	2.819298036	9.008988152	1.38E-17
PEG10	1.179208961	2.320609697	5.903828687	8.42E-09
DEPP1	1.183201689	5.960247767	9.666084736	9.64E-20
LTBP2	1.185430031	4.700641549	9.881788501	1.81E-20
CLU	1.185704289	6.956503766	6.324984281	7.75E-10
MSRB3	1.189190853	3.721893751	8.223280212	3.94E-15
SERPINF1	1.190827525	6.398117635	9.047459218	1.04E-17
SCARA3	1.19491862	3.443655594	8.680871765	1.52E-16
MEDAG	1.195791361	2.722690091	9.638935883	1.19E-19
TFF2	1.195942963	5.831194087	3.153493455	0.001753108
SGCA	1.19698453	2.273896898	8.098220838	9.41E-15
SNCG	1.197202954	3.094781227	8.739647867	9.94E-17
THBS1	1.199425777	6.412305072	8.843212631	4.67E-17
RSPO3	1.202284157	2.860223229	7.733361293	1.13E-13
MIR100HG	1.205533144	2.672917825	8.577143525	3.21E-16
SLC7A2	1.206656063	2.32359976	7.884339462	4.08E-14
MFAP2	1.206666787	3.982257054	8.92834535	2.50E-17
CPXM2	1.207617231	3.361564053	7.251639544	2.67E-12
FBLN2	1.208402569	4.407677303	7.854752698	4.99E-14
CKB	1.20855386	6.139598752	6.85138841	3.31E-11
SMOC2	1.209187327	4.759885465	6.680125116	9.43E-11
NDNF	1.2110927	1.856512439	9.562359065	2.14E-19
ROR2	1.211286653	2.735626043	9.403212728	7.20E-19
CYS1	1.213715806	2.044744106	9.138154418	5.29E-18
TPM2	1.214209255	7.098396207	7.72428383	1.20E-13
ANTXR1	1.21795002	5.509314867	9.607831879	1.51E-19
CYBRD1	1.218159067	5.104531537	9.086228548	7.79E-18
DACT3	1.219036809	2.772015024	8.694172185	1.38E-16
AEBP1	1.219234352	6.919584612	8.816352999	5.69E-17
FGF7	1.219783346	2.600578254	9.287819666	1.72E-18
F13A1	1.226935174	2.791045234	7.509035823	5.02E-13
RAB34	1.227225706	5.226312395	10.3752826	3.67E-22
ANPEP	1.229495345	5.806288543	4.344678124	1.83E-05
MOXD1	1.232051019	3.220525494	9.569885672	2.02E-19
RGMA	1.236264327	2.375225598	7.417127588	9.16E-13
CST2	1.239356371	2.672753201	7.522652322	4.59E-13
THBS2	1.239892043	5.277038658	7.071512148	8.40E-12

DCN	1.244526214	6.155547029	9.425217729	6.09E-19
PSCA	1.245315705	5.548307066	3.54816256	0.000440926
MMP2	1.247371978	7.124544008	9.252409516	2.25E-18
MRVI1	1.249678563	3.871471518	9.001373444	1.46E-17
CYP1B1	1.252797526	3.017956455	7.353879194	1.38E-12
SLC22A17	1.253669829	2.925349319	11.00927993	2.13E-24
LRRN1	1.256594493	1.615026195	8.454530795	7.72E-16
FBLN5	1.260435643	4.993429141	10.44981671	2.02E-22
SPARCL1	1.260734668	6.764269437	8.14496356	6.80E-15
LIPF	1.263351924	3.550744019	3.039053528	0.002552123
PALM	1.264023328	3.434228017	9.581700262	1.84E-19
SRPX	1.267855597	3.034876926	9.532308548	2.69E-19
RERG	1.270637938	2.749794015	9.578137826	1.90E-19
PGM5	1.275140653	2.869933606	6.565121851	1.88E-10
MFAP5	1.277598297	3.357596503	8.053577768	1.28E-14
COL10A1	1.278354295	3.700516531	6.037737736	4.00E-09
ISLR	1.285556155	5.683641471	7.703194953	1.38E-13
MRGPRF	1.288536307	3.638434122	7.821431042	6.25E-14
SERPINA5	1.290379181	2.218904156	8.221287624	4.00E-15
PDLIM3	1.299469536	4.435114494	8.390478104	1.22E-15
BOC	1.300563967	2.56505817	9.124391487	5.86E-18
ADH1B	1.300829807	2.402351059	6.656198056	1.09E-10
RBPMS2	1.309475657	3.164061254	7.796190013	7.41E-14
ACTA2	1.316546324	8.118292007	8.55434711	3.79E-16
GPC3	1.317820254	3.243023567	6.38557598	5.44E-10
AOC3	1.322608024	4.09263272	8.550026033	3.90E-16
PPP1R14A	1.324439611	4.283728262	9.148269693	4.90E-18
FHL1	1.326069687	5.135961487	7.163250483	4.70E-12
SPOCK1	1.327155722	3.245952124	8.046165313	1.35E-14
SYNM	1.327910866	3.447336232	5.603425465	4.27E-08
CRYAB	1.328275725	4.592980592	8.368760643	1.42E-15
ACKR1	1.32833273	3.484924479	7.318715419	1.73E-12
RBP4	1.33594531	3.457047526	5.869810857	1.01E-08
TNS1	1.339314764	5.456201115	8.506409437	5.33E-16
PLN	1.343934432	3.201799772	6.827864146	3.83E-11
VSTM2L	1.344414501	2.000586918	8.244353679	3.40E-15
ADCY5	1.346564801	2.220989987	8.970133201	1.84E-17
PDK4	1.349573173	4.533799601	7.670618782	1.72E-13
DPYSL3	1.3590345	5.398598033	8.563308722	3.55E-16
SPINK4	1.359783077	3.833775778	4.185169372	3.61E-05
AGT	1.360975237	4.769776455	8.260498989	3.04E-15
HSPB8	1.361202448	3.953075273	7.808315987	6.83E-14
AKAP12	1.369170539	3.420065463	9.692833005	7.85E-20
MYLK	1.383680227	5.026923597	7.616831731	2.46E-13
CPE	1.396201449	4.128956873	10.19115012	1.59E-21
MXRA8	1.402762363	5.969632574	10.7447499	1.86E-23
COL14A1	1.405980382	3.969119213	8.981405157	1.69E-17
CCDC80	1.414092407	3.898833975	8.342935998	1.70E-15
CHRDL1	1.414433051	1.981854866	8.438896425	8.63E-16
ECRG4	1.430270936	2.632399157	6.266162094	1.09E-09
SFRP1	1.444671458	2.107786122	7.994602005	1.92E-14
FNDC1	1.449427786	3.752949876	7.960103647	2.43E-14
COL8A1	1.454674545	4.232220101	9.351921964	1.06E-18

APOA1	1.454920258	2.417868889	5.378778698	1.38E-07
HMGCS2	1.456545854	4.765222918	5.26793366	2.42E-07
EFEMP1	1.478748998	4.986494027	9.819821829	2.93E-20
MYL9	1.492707186	7.466252858	8.599711597	2.73E-16
FLNC	1.493437618	3.976301158	6.697401931	8.49E-11
DKK1	1.497220477	2.765366605	5.936273501	7.03E-09
TAGLN	1.49788078	6.953168989	8.265511686	2.93E-15
BARX1	1.510059251	4.253273091	7.832244961	5.81E-14
HSPB7	1.515615172	3.085560294	7.166130954	4.61E-12
COMP	1.515842554	2.734713575	7.558702511	3.62E-13
PGC	1.517966467	6.980637751	3.458615808	0.000610074
LMOD1	1.519289	4.066236412	7.357651535	1.35E-12
SYNPO2	1.521326095	4.061951786	6.396591989	5.10E-10
GREM1	1.533375804	4.784621837	8.31770023	2.03E-15
PCSK1N	1.534041443	3.67290658	5.521565018	6.57E-08
MGP	1.546887068	7.403526079	8.738875544	1.00E-16
SPON1	1.550779116	4.291237744	8.923490169	2.59E-17
ITLN1	1.552248117	3.017152393	5.597284711	4.41E-08
PODN	1.574670852	4.582293592	10.37586058	3.65E-22
WFDC2	1.588846932	5.2596051	5.921154774	7.65E-09
ELN	1.617015688	4.833953191	10.80057187	1.18E-23
ACTG2	1.624200769	6.936733334	5.814973588	1.37E-08
PTGIS	1.625038139	3.183092099	9.238925124	2.49E-18
PRELP	1.642704357	3.558711816	8.665900733	1.70E-16
MFAP4	1.673981301	6.385369243	9.779169556	4.02E-20
OGN	1.681265565	3.005299523	7.639017603	2.12E-13
FBLN1	1.68673833	6.259287217	9.407240937	6.98E-19
CNN1	1.697422201	5.813863091	6.597197088	1.55E-10
C7	1.706142999	3.080627379	7.36835139	1.26E-12
HSPB6	1.730635947	5.230726301	6.595970771	1.57E-10
CHRDL2	1.762383162	4.064723373	8.128160302	7.65E-15
SFRP4	1.833486293	4.790825657	7.88518003	4.06E-14
MYH11	1.894998396	6.453841163	6.706779346	8.03E-11
DES	1.901516153	6.328971618	5.415183505	1.14E-07
THBS4	1.922673246	3.453146863	8.080011149	1.07E-14
APOD	2.03784399	5.9231248	8.651222295	1.89E-16
SFRP2	2.05066345	5.660198841	7.812542558	6.63E-14

adj.P.Val	B
5.77E-07	8.80957102
0.000257599	2.333577107
0.000410567	1.833193308
3.62E-06	6.89055213
3.39E-09	14.22992669
3.33E-08	11.81474613
0.000734128	1.213629755
3.20E-06	7.020919695
0.009812371	-1.593012168
0.000103118	3.311977207
7.04E-08	11.03065935
0.000100338	3.341072611
9.01E-06	5.917764309
0.022141823	-2.491141198
0.015343028	-2.087648616
0.000258112	2.331416064
0.009610222	-1.571022562
8.97E-10	15.62460661
0.008899083	-1.487966341
0.004449783	-0.73077475
0.006585777	-1.156758658
4.19E-06	6.736714457
9.75E-06	5.835517203
0.02020399	-2.39045014
3.12E-07	9.456435876
3.86E-05	4.369025726
0.000106971	3.274308756
0.002634705	-0.162775796
7.80E-06	6.068761435
9.03E-05	3.45270236
0.000833235	1.079074656
0.002194363	0.033754961
4.71E-05	4.155260297
6.75E-09	13.48217066
1.01E-12	23.0038349
2.27E-08	12.21761476
4.54E-05	4.194261777
1.43E-07	10.27876379
0.001316306	0.582009245
7.69E-07	8.508574194
6.17E-09	13.58008252
0.036017277	-3.031306559
6.77E-09	13.4792509
0.000754768	1.183790686
1.10E-08	12.97482088
2.16E-13	24.72011963
3.21E-05	4.568264091
0.00020207	2.593141227
0.002376244	-0.053425055

5.96E-06	6.361654314
1.31E-14	27.96005947
0.003570905	-0.493073623
1.45E-14	27.84195625
6.19E-05	3.857098497
1.18E-08	12.90404675
5.52E-09	13.70229521
5.01E-06	6.542139715
3.12E-12	21.77733183
5.79E-11	18.56536039
3.55E-06	6.910821188
2.02E-10	17.21466309
0.00010999	3.2455114
4.36E-14	26.55019782
4.63E-09	13.89640653
6.85E-05	3.747422474
4.09E-11	18.94573405
1.69E-06	7.685583314
0.001987776	0.140821032
7.37E-07	8.55333293
4.20E-05	4.277567815
0.000430521	1.781090405
2.22E-14	27.30635337
2.65E-09	14.49536302
4.33E-05	4.242899575
4.11E-05	4.301170365
2.07E-07	9.890209806
6.42E-05	3.8183044
2.91E-10	16.82767159
0.003889237	-0.58620739
3.77E-11	19.03288917
9.45E-05	3.404696827
5.06E-05	4.076562896
1.15E-07	10.51257004
3.30E-05	4.535572773
1.42E-09	15.14721201
1.80E-09	14.88934912
0.000644469	1.353212635
2.97E-06	7.097417379
9.53E-11	18.03361967
0.000113421	3.212848858
7.10E-15	28.61517504
6.56E-08	11.10140873
7.61E-13	23.32071464
2.00E-11	19.72961785
1.78E-14	27.58706846
4.34E-06	6.697340722
1.37E-09	15.18728639
2.91E-11	19.30432619
1.75E-08	12.49132752
0.000155931	2.871310675
1.38E-06	7.895633068
0.000204596	2.580191277

4.50E-13	23.90997289
1.15E-13	25.42451229
1.41E-10	17.61409345
1.34E-06	7.930455515
3.59E-12	21.61233371
0.002567162	-0.134364908
1.00E-09	15.50798286
3.67E-11	19.05973905
1.27E-12	22.75463987
1.16E-08	12.91991076
1.10E-11	20.38161629
5.96E-06	6.362317378
2.99E-08	11.92920645
5.09E-08	11.36920978
1.85E-06	7.587468763
5.57E-05	3.969874793
0.00018432	2.694232012
0.002569839	-0.135498375
1.66E-12	22.45275919
8.37E-10	15.69767551
7.10E-15	28.61042173
5.43E-05	3.997497058
1.83E-15	30.28403364
8.53E-07	8.399643065
0.000191165	2.654045101
2.68E-12	21.93771247
4.40E-17	34.92227056
1.32E-07	10.3628206
1.66E-09	14.97904204
5.20E-07	8.921398576
0.001407656	0.510388245
7.21E-06	6.154389701
7.93E-05	3.592533463
0.001553831	0.404818464
6.42E-13	23.50576689
4.51E-09	13.92376343
1.78E-13	24.94532074
3.47E-08	11.76990534
4.70E-06	6.610861519
5.49E-14	26.29057598
7.72E-09	13.34000113
0.000383203	1.906729242
1.67E-11	19.91964594
3.30E-08	11.82544685
1.40E-06	7.879843094
1.40E-15	30.57868105
0.000606543	1.415312692
2.00E-10	17.22895305
6.60E-09	13.50752724
3.79E-10	16.54654767
2.71E-10	16.90337744
0.000475108	1.673043938
1.75E-10	17.37643492

1.17E-07	10.49252174
3.83E-09	14.10064979
9.09E-11	18.08414969
2.76E-14	27.07291377
6.01E-05	3.887403868
1.44E-12	22.61851595
7.26E-09	13.407817
1.04E-11	20.44073499
0.011726616	-1.787960707
3.90E-11	18.99486374
1.73E-05	5.230627248
5.05E-10	16.23558646
2.23E-16	32.75830114
9.28E-08	10.73874007
1.10E-09	15.41598807
3.05E-08	11.90790385
5.96E-06	6.362238723
4.92E-12	21.26827877
4.96E-05	4.097613646
8.77E-09	13.20698864
1.00E-05	5.808188554
2.49E-12	22.01499741
4.36E-06	6.691628772
1.93E-05	5.110255311
0.000763604	1.172480259
4.55E-06	6.644281918
1.85E-09	14.86149497
3.23E-06	7.009609907
4.78E-13	23.83991898
0.001200095	0.680405049
0.044359267	-3.271169809
5.11E-05	4.063828489
1.29E-09	15.24730392
3.86E-11	19.00565493
4.98E-07	8.965771755
1.50E-10	17.54503981
1.31E-10	17.69670286
3.40E-05	4.503486737
2.26E-09	14.65945634
3.83E-05	4.377802155
5.82E-13	23.61460126
3.14E-08	11.88035373
3.38E-10	16.66477044
1.74E-07	10.07193525
7.30E-13	23.36803001
1.58E-12	22.50484276
0.000164915	2.811308728
7.56E-06	6.102646099
3.95E-13	24.0604551
0.008054465	-1.37720238
1.56E-11	19.99451372
1.55E-10	17.51253111
7.92E-05	3.59359123

6.82E-12	20.90152987
0.003262259	-0.395368471
0.015876335	-2.124654229
1.25E-08	12.83906861
3.27E-06	6.995329348
8.61E-09	13.22581128
2.66E-07	9.621443769
2.88E-10	16.83747488
0.001207811	0.673108617
3.84E-08	11.66597651
5.12E-10	16.22108988
2.19E-12	22.1597132
2.33E-12	22.09007399
4.11E-08	11.59591123
3.32E-15	29.5322626
6.59E-14	26.06944126
0.000124052	3.119295206
5.15E-09	13.77648882
3.06E-14	26.95511599
7.56E-07	8.526087648
3.57E-11	19.08902037
0.0224898	-2.508908019
1.20E-05	5.612052833
8.27E-08	10.86005054
0.000126896	3.095495434
3.41E-08	11.78901105
2.82E-08	11.99269105
4.65E-05	4.169430109
2.84E-06	7.144154226
4.28E-09	13.98246401
1.77E-07	10.05700031
5.33E-09	13.73765524
5.36E-05	4.012411587
2.04E-11	19.70365253
7.85E-06	6.063213923
3.00E-07	9.497375322
4.68E-10	16.31567319
2.33E-08	12.18916017
7.00E-10	15.88685755
9.70E-12	20.52340812
4.49E-11	18.84319267
3.38E-12	21.68460374
4.64E-11	18.80482497
2.88E-08	11.97280122
1.34E-07	10.34802724
3.11E-10	16.75093527
2.06E-08	12.31787718
6.03E-09	13.60394237
1.05E-09	15.4631255
0.001226813	0.657034933
5.99E-09	13.6128821
2.58E-05	4.803178449
2.81E-10	16.8639303

9.88E-16	30.95271255
9.73E-05	3.373447046
2.24E-08	12.23187843
6.54E-10	15.96247686
1.23E-13	25.35420469
1.42E-05	5.441697811
0.001107343	0.77064004
2.44E-11	19.49995894
0.000707495	1.251729856
0.002639813	-0.165233883
1.20E-09	15.32245928
2.96E-10	16.80760364
3.51E-09	14.19407821
0.000204409	2.581298344
7.86E-12	20.7554991
6.05E-06	6.345098489
1.40E-05	5.453600098
8.89E-13	23.14757369
5.20E-07	8.92044104
8.24E-05	3.551391147
3.84E-10	16.53151468
2.22E-07	9.818684689
1.37E-11	20.13152628
2.95E-07	9.512605581
2.47E-05	4.848230987
1.15E-11	20.32645915
1.76E-08	12.48438004
8.43E-05	3.526146286
1.69E-10	17.41278048
4.38E-12	21.39310942
3.13E-05	4.594471098
3.39E-08	11.79546768
2.41E-10	17.0279186
1.28E-11	20.19962197
2.57E-11	19.44134784
2.37E-10	17.04786001
1.99E-12	22.2717434
2.84E-06	7.146992088
0.013386001	-1.936343168
1.75E-10	17.3722748
4.93E-08	11.40463369
1.80E-11	19.8395197
1.30E-08	12.79930746
1.97E-10	17.24927606
1.57E-12	22.51514307
1.09E-11	20.39681742
0.001088351	0.789132892
0.040051941	-3.152358751
0.000147702	2.93019204
1.16E-07	10.50008135
2.82E-12	21.88215511
0.00022865	2.461499227
1.42E-07	10.29011292

6.92E-05	3.736517819
1.23E-08	12.85417922
5.48E-06	6.448659769
3.50E-11	19.11096715
1.59E-05	5.32230178
5.26E-08	11.33301392
9.72E-08	10.68753716
2.45E-11	19.49700301
1.40E-09	15.16225555
0.013965886	-1.982242609
3.56E-12	21.62308522
1.79E-06	7.626088524
0.000515531	1.588401488
1.95E-10	17.2626358
6.66E-09	13.4959692
5.73E-13	23.63527855
3.52E-05	4.466871454
1.17E-09	15.35176941
0.000110081	3.244254408
2.26E-09	14.65810481
2.12E-05	5.011559872
3.99E-10	16.49210305
5.09E-14	26.37659546
1.94E-05	5.105529082
0.002628697	-0.160161306
1.68E-05	5.263048661
0.002231331	0.016171955
0.000125952	3.103087169
2.34E-06	7.351277547
6.48E-11	18.44665129
9.14E-12	20.5907113
5.73E-13	23.63386668
3.22E-12	21.73836265
1.81E-11	19.83081613
0.021221732	-2.443027366
6.98E-10	15.89007468
1.59E-06	7.753112096
0.000325434	2.081738402
2.58E-08	12.08600246
1.95E-10	17.26044247
0.008764142	-1.472408005
8.55E-09	13.23337293
1.91E-16	32.97065828
0.002432412	-0.079142048
4.45E-05	4.215818818
3.56E-08	11.74151347
1.57E-14	27.72063532
9.11E-14	25.69146828
7.69E-09	13.34438339
4.99E-07	8.962284798
1.37E-09	15.18923456
0.009645049	-1.574974103
1.43E-07	10.28137911

0.041851097	-3.204519844
2.39E-09	14.60371749
0.002758245	-0.214825213
3.92E-12	21.51803169
1.12E-11	20.36159447
1.42E-09	15.14912361
2.24E-05	4.949476552
4.94E-12	21.26190386
5.34E-11	18.65072409
5.21E-07	8.917261931
1.65E-10	17.4397178
2.68E-07	9.614032072
1.81E-14	27.5664225
2.22E-14	27.30831172
3.34E-07	9.382456982
5.46E-06	6.453627132
1.95E-06	7.537629398
6.31E-07	8.717162481
1.22E-09	15.30531459
1.67E-08	12.53549823
2.17E-09	14.70141384
1.30E-08	12.79740191
3.81E-08	11.67414612
1.93E-07	9.964190393
1.24E-08	12.84551091
3.25E-07	9.413323784
3.08E-06	7.061015369
2.04E-07	9.907745126
2.30E-11	19.5691347
8.83E-09	13.19934796
1.04E-08	13.02778785
3.34E-08	11.81188522
1.02E-13	25.55898217
0.000743855	1.200278698
3.55E-10	16.61293434
3.28E-12	21.71945874
1.33E-10	17.68032355
1.23E-14	28.0261619
0.000941149	0.948843683
6.71E-11	18.40926664
0.000232356	2.445611187
1.84E-10	17.32122344
3.13E-13	24.32848084
1.35E-16	33.49627496
2.58E-08	12.08485447
2.57E-11	19.44258587
0.022987305	-2.532948717
4.06E-05	4.312833324
2.06E-11	19.69386913
2.43E-11	19.50906711
3.95E-09	14.06859623
0.006477303	-1.137160029
7.60E-09	13.35798278

2.44E-07	9.715327317
1.16E-05	5.647072432
1.52E-14	27.78228058
9.29E-07	8.30986202
1.02E-13	25.56064737
8.73E-10	15.65453773
1.93E-07	9.965730039
2.44E-07	9.714822357
1.13E-06	8.105012809
2.42E-10	17.02628504
0.028748117	-2.777075596
2.99E-09	14.36635643
5.05E-08	11.37866983
2.47E-05	4.851154353
2.57E-12	21.97927902
1.17E-11	20.30508068
3.21E-05	4.56691589
5.20E-09	13.76654989
6.05E-10	16.04567638
9.85E-17	33.98565108
3.92E-07	9.214321105
3.65E-09	14.15208969
2.28E-16	32.7269881
1.24E-08	12.84605731
1.19E-07	10.46595741
4.46E-15	29.18814022
1.25E-11	20.23576044
1.73E-11	19.87809513
1.07E-07	10.58499681
2.71E-10	16.90217871
1.15E-11	20.32010341
3.99E-14	26.65049136
9.95E-09	13.07914697
0.000204166	2.582666548
4.27E-05	4.259467634
1.75E-05	5.217415434
3.13E-09	14.3129851
4.63E-07	9.040091733
1.96E-13	24.83061436
0.01759675	-2.236436627
3.84E-05	4.376174849
0.000115165	3.195952948
2.60E-11	19.42780645
8.58E-05	3.505375638
3.18E-14	26.91249463
8.85E-06	5.937899726
6.56E-14	26.07962144
3.71E-09	14.13434256
7.65E-16	31.26535971
1.52E-10	17.53004309
2.18E-11	19.63237144
1.34E-05	5.50094028
4.70E-07	9.022574096

2.48E-13	24.57625961
1.72E-09	14.94326562
3.34E-13	24.24806261
9.62E-12	20.53467203
1.47E-12	22.59066581
5.48E-06	6.44911991
4.29E-11	18.89430417
1.64E-07	10.13566272
1.02E-10	17.95946804
1.77E-09	14.91553168
3.64E-14	26.75550487
5.38E-08	11.30889884
0.002061833	0.101612664
4.34E-10	16.39933837
1.45E-07	10.26385925
1.25E-07	10.42348283
0.000348036	2.009210531
5.62E-09	13.68276606
5.79E-14	26.2255655
1.94E-05	5.101226652
1.55E-12	22.53523881
7.90E-11	18.23751116
2.37E-11	19.53374801
6.92E-10	15.90056927
8.63E-13	23.18011499
4.67E-09	13.88517484
4.97E-12	21.25467661
9.48E-05	3.400436422
1.16E-16	33.75415547
7.62E-10	15.79878787
3.51E-08	11.75665701
0.000343718	2.022319239
1.01E-07	10.65182205
7.49E-09	13.37421867
3.67E-06	6.875342467
5.92E-11	18.54307086
5.22E-11	18.67591878
1.37E-10	17.64125963
8.51E-09	13.23861342
5.90E-13	23.59681182
8.06E-12	20.72545449
1.67E-06	7.700560255
1.71E-11	19.89016183
2.20E-14	27.32263189
6.51E-09	13.5222729
1.15E-06	8.088545504
2.55E-07	9.664344802
3.30E-05	4.53568045
1.29E-10	17.70974925
1.10E-05	5.707863846
3.63E-10	16.59050838
6.80E-06	6.216321291
2.83E-07	9.556366581

5.86E-10	16.07837951
1.11E-08	12.96750984
4.42E-07	9.086837745
0.00127131	0.619296792
1.02E-13	25.56925598
5.36E-10	16.17033046
4.87E-11	18.7530513
1.29E-12	22.73432198
0.032156361	-2.90429632
7.62E-10	15.80000558
0.000190649	2.657870231
3.40E-08	11.79277413
7.12E-13	23.39641298
5.32E-05	4.019201621
8.62E-14	25.75693051
0.007396181	-1.284135346
1.94E-08	12.37982883
2.53E-11	19.461011
7.40E-09	13.38725923
1.07E-07	10.58231711
0.000836382	1.075144922
1.52E-10	17.53165793
1.30E-08	12.79939288
0.000299224	2.168846308
2.22E-10	17.11692861
1.76E-06	7.641630933
1.69E-12	22.43465592
2.63E-11	19.41249515
1.33E-18	39.35487091
4.24E-07	9.131480953
0.000190996	2.655192302
8.46E-12	20.67525759
4.72E-05	4.151018406
5.26E-12	21.18558824
1.58E-07	10.17083397
4.63E-12	21.33773719
0.046810418	-3.332169421
1.12E-14	28.13004667
9.63E-14	25.6344109
1.06E-16	33.86879476
6.10E-10	16.03550542
1.78E-05	5.201839488
1.16E-08	12.92086513
5.79E-06	6.391964442
9.08E-10	15.61237326
2.01E-09	14.77717807
1.47E-08	12.67152216
5.14E-12	21.21615208
1.75E-13	24.9632496
5.73E-13	23.63375884
1.42E-12	22.63766901
2.20E-11	19.61743639
1.00E-12	23.01501073

1.99E-12	22.27360988
3.38E-14	26.83568265
1.66E-10	17.43027624
8.52E-06	5.97809316
9.53E-11	18.03452226
4.30E-10	16.40949302
3.95E-13	24.05712229
5.68E-08	11.24909712
0.03386031	-2.96213582
2.52E-10	16.98038175
1.09E-06	8.144831315
6.17E-10	16.02212025
3.15E-13	24.31739516
0.048363771	-3.368326764
6.25E-12	20.99524716
5.68E-07	8.827046085
2.24E-15	30.02705536
4.39E-07	9.096725647
2.03E-12	22.24558999
8.78E-08	10.79585452
1.82E-13	24.91316099
4.15E-09	14.0147918
0.026697647	-2.69668528
4.37E-10	16.39291112
4.66E-12	21.32836195
5.25E-05	4.032566131
4.01E-08	11.62167906
3.33E-05	4.526304371
1.75E-06	7.646460518
3.24E-11	19.19535156
4.70E-07	9.024660575
5.39E-11	18.63820722
0.001658484	0.333263727
9.70E-14	25.62147715
1.98E-05	5.081116681
3.00E-10	16.79062427
4.58E-05	4.185252884
3.08E-08	11.89721154
1.27E-11	20.21405606
5.55E-08	11.2726391
2.39E-09	14.60034381
4.89E-09	13.83003794
8.43E-08	10.83860232
7.31E-16	31.32730145
2.87E-19	41.95944801
8.27E-13	23.22733029
3.95E-09	14.06641764
8.07E-07	8.457932012
6.34E-12	20.97846088
6.92E-11	18.37653621
2.07E-14	27.41574503
8.63E-07	8.387861818
6.59E-14	26.06581879

3.25E-11	19.19053853
2.83E-07	9.556764427
2.99E-15	29.6639042
4.78E-12	21.30052487
8.02E-09	13.29854488
3.17E-13	24.31130872
7.98E-10	15.74758554
3.07E-09	14.33433859
1.13E-11	20.34872758
1.00E-11	20.49009056
4.64E-09	13.89258119
5.22E-10	16.19779404
3.04E-12	21.80776077
1.02E-11	20.46732704
7.69E-12	20.77821121
3.23E-08	11.84673025
0.00116636	0.712867671
2.38E-16	32.67604825
6.00E-09	13.60967498
1.12E-12	22.88516629
1.28E-12	22.74075609
2.19E-14	27.33860124
0.017711636	-2.244687584
4.64E-11	18.80363397
1.84E-10	17.3206097
4.77E-13	23.84620999
1.33E-13	25.26292098
7.65E-10	15.79364338
2.48E-10	16.99983152
2.56E-15	29.84220668
7.96E-11	18.22384216
5.28E-11	18.66322801
8.23E-10	15.71632925
1.06E-09	15.45056178
4.97E-07	8.967593264
4.93E-14	26.4163698
2.87E-09	14.40681997
3.16E-11	19.22131262
2.48E-15	29.88902683
1.21E-16	33.66721424
0.000140463	2.984634237
1.74E-12	22.40445739
7.78E-13	23.29513612
1.17E-07	10.49041066
4.09E-11	18.94368233
1.62E-08	12.57503446
1.19E-07	10.46746273
4.61E-10	16.33288028
2.69E-13	24.49006086
6.39E-14	26.10881513
1.18E-11	20.29258492
5.02E-13	23.78681181
2.96E-11	19.28616022

6.65E-08	11.08835093
4.87E-14	26.43365487
6.37E-05	3.826310774
8.65E-12	20.65316669
4.97E-09	13.81445611
0.000885991	1.013642103
1.03E-07	10.623642
1.80E-11	19.83948378
4.32E-12	21.40869467
3.16E-07	9.443427749
5.91E-17	34.58087229
6.83E-11	18.39105567
1.40E-07	10.30002138
9.75E-10	15.53852923
2.08E-13	24.76073784
3.42E-12	21.66891489
2.11E-09	14.72890764
7.28E-16	31.35275677
7.77E-09	13.33178294
6.55E-12	20.94595368
3.54E-07	9.323444713
5.02E-10	16.24152484
2.91E-10	16.82349689
4.35E-08	11.53513278
1.25E-08	12.83593633
9.60E-16	31.00711567
2.03E-12	22.23986839
6.18E-08	11.16435756
7.96E-11	18.22491577
5.40E-08	11.302437
2.33E-10	17.06621831
2.72E-10	16.89872834
0.000327486	2.073994884
2.23E-07	9.811883804
4.45E-08	11.50922225
1.50E-08	12.64945727
2.73E-10	16.89424074
3.86E-11	19.00661963
1.14E-14	28.1041747
4.43E-10	16.37691424
1.04E-07	10.61950877
0.000144884	2.950710689
0.042906599	-3.232578142
3.25E-09	14.27570894
4.51E-08	11.49448811
4.51E-11	18.83662435
3.74E-06	6.856576215
5.67E-09	13.6727977
1.49E-08	12.65675715
1.21E-17	36.6604757
2.32E-13	24.64583627
6.52E-06	6.263257097
4.40E-13	23.93555623

5.40E-07	8.879980396
0.008526848	-1.441091115
9.28E-08	10.7376932
1.96E-07	9.950666235
4.25E-06	6.719697561
1.09E-10	17.88947889
6.93E-13	23.42664511
2.41E-09	14.59251039
1.13E-08	12.94382675
1.31E-07	10.36989206
6.49E-10	15.97194409
2.67E-06	7.212470505
2.08E-12	22.21857067
3.25E-09	14.27265107
1.76E-08	12.48258635
2.05E-10	17.19891859
8.38E-13	23.21058243
1.20E-11	20.27419006
3.86E-12	21.53661855
4.36E-09	13.95977485
1.29E-10	17.71723957
9.32E-12	20.56942393
1.59E-16	33.22389264
1.36E-10	17.64962187
4.13E-12	21.45800804
5.16E-10	16.21202384
1.11E-10	17.86765054
2.19E-14	27.33397033
4.18E-08	11.57703949
3.19E-08	11.86249334
2.65E-11	19.40188299
3.93E-07	9.21221705
5.21E-12	21.20249498
6.64E-14	26.05208816
1.10E-11	20.37992234
2.83E-10	16.85575736
1.52E-10	17.52811912
4.93E-14	26.41507341
8.03E-12	20.73064396
2.36E-10	17.05120678
1.55E-12	22.52976297
4.99E-05	4.089989558
1.96E-10	17.25312408
1.47E-09	15.10847836
1.61E-06	7.740185921
8.80E-06	5.943492392
2.57E-09	14.52494299
3.92E-12	21.51611263
1.42E-11	20.08960332
7.94E-11	18.2286218
2.53E-09	14.54123798
5.60E-05	3.963914075
3.73E-13	24.12779081

5.23E-12	21.19555054
5.93E-12	21.05565675
0.041662832	-3.198711565
3.05E-09	14.34435173
6.04E-10	16.0472361
8.89E-13	23.14313919
1.25E-06	7.996817882
1.09E-14	28.15710169
4.77E-09	13.85886627
1.75E-11	19.86898742
2.48E-14	27.18513368
4.44E-09	13.94178849
0.001370627	0.539478374
3.70E-09	14.13895502
2.03E-15	30.15054398
6.54E-09	13.51643679
7.59E-12	20.79174303
2.35E-12	22.08095676
0.000249099	2.36971486
1.00E-09	15.50713409
0.049066249	-3.384561659
1.36E-13	25.23299408
1.09E-07	10.57052252
8.54E-10	15.67705884
2.39E-11	19.52547525
1.36E-16	33.45317131
2.48E-14	27.17976947
7.65E-09	13.35024964
1.60E-12	22.49056422
1.61E-15	30.42747007
2.91E-11	19.30854199
0.000431278	1.779000463
7.82E-10	15.76982834
1.68E-14	27.65549115
4.32E-09	13.96935097
3.84E-13	24.09424508
1.25E-15	30.70377229
2.60E-08	12.07601859
1.10E-09	15.41001505
1.16E-09	15.3557601
6.93E-13	23.42493817
1.07E-08	12.9985539
2.37E-15	29.94977021
0.001275676	0.615352908
4.13E-10	16.45607885
6.82E-06	6.212473649
3.52E-07	9.329063374
1.98E-10	17.23988654
1.35E-09	15.20470482
6.53E-13	23.48688169
1.97E-09	14.80002009
3.28E-12	21.71778307
1.52E-10	17.53188414

2.35E-05	4.904509895
2.09E-10	17.18005171
5.45E-08	11.2909138
9.23E-10	15.59348719
2.54E-15	29.85401819
1.04E-13	25.53325446
0.000491701	1.636708147
1.42E-15	30.55969312
8.32E-14	25.79457195
2.86E-09	14.41202496
8.81E-11	18.11886939
9.35E-08	10.72995493
3.22E-15	29.57904855
4.29E-10	16.41392555
1.50E-14	27.80234124
2.49E-08	12.12459887
3.58E-11	19.08622886
7.12E-15	28.59857671
1.41E-11	20.09887928
2.60E-11	19.42456592
7.86E-11	18.24267279
2.82E-11	19.34004188
1.91E-11	19.77727984
6.22E-12	21.00027421
1.14E-07	10.51912732
2.26E-14	27.28394859
4.16E-09	14.01007596
5.00E-09	13.80761097
8.10E-13	23.25040836
1.44E-16	33.33417975
1.50E-12	22.57204548
1.29E-10	17.71320143
9.85E-17	33.99259916
3.28E-14	26.88015332
6.01E-14	26.18006098
3.46E-05	4.487857221
2.30E-14	27.26481538
7.35E-08	10.98269437
6.92E-15	28.64937769
4.12E-06	6.753744847
2.14E-16	32.80925564
4.48E-08	11.5022741
1.08E-12	22.93940682
0.00250347	-0.108047851
1.87E-07	10.00125092
5.26E-09	13.75385943
4.24E-12	21.43032328
1.32E-11	20.16940652
1.55E-11	20.00147356
4.07E-14	26.62424654
2.77E-10	16.87836243
2.62E-10	16.9402205
7.24E-11	18.32880021

2.78E-12	21.89683225
2.28E-15	30.00378008
2.71E-07	9.600795217
2.23E-18	38.80000818
1.79E-15	30.3190304
1.42E-16	33.35679605
2.28E-09	14.64927635
0.002990909	-0.30160924
3.46E-05	4.487493704
2.61E-05	4.787427665
1.62E-09	15.00580327
1.09E-10	17.88508347
1.53E-14	27.75689185
6.01E-14	26.18263068
6.14E-13	23.55549568
3.35E-11	19.15799123
4.77E-09	13.85968974
6.85E-14	26.01366731
1.73E-14	27.61900354
7.92E-10	15.75680778
2.29E-10	17.08725784
6.58E-10	15.95575152
0.0002333	2.441064585
3.52E-10	16.62394699
1.49E-10	17.55809989
3.61E-07	9.300279838
1.98E-10	17.24062069
4.14E-08	11.58770874
9.73E-05	3.372825601
1.48E-17	36.28422801
4.23E-13	23.98465715
9.70E-14	25.62468467
4.97E-05	4.096591071
6.66E-11	18.41887887
2.95E-05	4.657463975
9.35E-11	18.05396478
1.67E-13	25.02111266
1.62E-13	25.05947694
5.53E-12	21.13121475
1.26E-16	33.61263223
3.13E-13	24.32709351
4.23E-10	16.42781802
4.83E-18	37.78986617
1.15E-13	25.42542564
1.17E-11	20.30200797
2.34E-17	35.69461189
7.17E-19	40.58528976
4.06E-07	9.177877892
1.33E-15	30.63214283
3.29E-09	14.2610549
1.18E-12	22.8329265
7.30E-14	25.94265284
4.25E-12	21.42761694

2.54E-12	21.99431364
3.63E-08	11.7199966
4.13E-11	18.93362921
3.61E-14	26.76880217
4.08E-15	29.30553755
5.69E-08	11.24785399
4.01E-14	26.6422948
5.14E-11	18.69336983
7.28E-16	31.35165784
1.34E-14	27.93147996
4.07E-13	24.02379413
1.23E-13	25.34786363
1.96E-08	12.37330629
5.40E-12	21.15489115
9.87E-11	17.99499611
4.18E-07	9.146555247
4.52E-09	13.92146804
3.16E-12	21.76383687
3.86E-16	32.09397152
2.01E-12	22.25903165
2.07E-10	17.1895667
7.67E-14	25.89148213
4.38E-08	11.52714984
1.49E-12	22.57836878
9.89E-16	30.94423415
6.97E-15	28.63788805
2.00E-11	19.72674013
1.19E-11	20.28148779
4.50E-10	16.35970408
1.91E-14	27.50447409
1.63E-16	33.18247779
1.53E-14	27.75505037
8.88E-10	15.63559261
9.47E-05	3.402332649
6.58E-15	28.73053724
1.42E-08	12.70610698
2.45E-10	17.00983476
3.63E-09	14.15849575
4.25E-09	13.99003145
5.92E-09	13.62455786
2.19E-12	22.16095822
3.84E-10	16.53082706
1.21E-10	17.78383282
8.74E-11	18.12908617
8.32E-09	13.26191812
4.63E-14	26.48881951
6.14E-13	23.55261314
1.48E-17	36.27613411
6.13E-11	18.50724184
1.50E-12	22.57045867
4.57E-10	16.34084044
4.66E-12	21.32886285
3.43E-13	24.21979384

6.29E-15	28.78332507
4.43E-11	18.85774616
3.25E-07	9.413454807
3.31E-09	14.25227908
1.70E-13	24.9963582
5.14E-08	11.35743957
1.95E-15	30.19726476
1.91E-14	27.50109625
8.74E-12	20.64164496
0.000118424	3.168230559
3.93E-11	18.98411971
1.22E-08	12.86639504
4.43E-11	18.85746855
4.95E-06	6.554761821
5.47E-17	34.69204225
5.19E-10	16.20608524
1.80E-09	14.89444637
4.03E-06	6.779118233
1.04E-06	8.199253545
2.61E-08	12.07347738
3.05E-12	21.80153325
5.58E-09	13.69048674
3.02E-10	16.78243304
4.84E-12	21.28684212
1.14E-11	20.33326764
5.26E-12	21.1853937
1.47E-12	22.59770417
5.03E-08	11.38223898
2.35E-11	19.54502025
7.93E-13	23.27525622
8.96E-14	25.71310632
6.16E-16	31.56223051
2.32E-11	19.55867621
4.67E-15	29.13240705
6.85E-10	15.91241954
2.14E-14	27.36398141
2.17E-17	35.85446322
1.57E-10	17.49506047
5.12E-11	18.69955382
3.22E-15	29.56812667
1.26E-09	15.27003365
1.35E-17	36.47230822
7.65E-16	31.27264946
4.19E-16	31.98732467
3.08E-05	4.611321117
1.99E-10	17.23478958
1.57E-10	17.49422377
1.43E-06	7.859427386
0.000776401	1.154483451
4.69E-09	13.87989839
1.14E-18	39.80177405
7.10E-12	20.8607763
4.88E-16	31.81272675

2.77E-14	27.06161139
2.19E-11	19.62655488
2.02E-07	9.918168757
1.57E-14	27.72754502
1.52E-09	15.07219995
1.23E-17	36.61856827
2.77E-11	19.35627323
3.86E-11	19.00465637
5.25E-14	26.34305963
9.85E-17	34.01172618
7.56E-06	6.102736865
6.15E-06	6.327906685
8.99E-13	23.12841221
2.48E-12	22.01829186
1.14E-15	30.7962747
0.000137557	3.008271538
0.008749732	-1.470377009
2.51E-10	16.98475757
3.77E-07	9.257249112
4.23E-10	16.42869267
4.37E-13	23.94743692
6.88E-10	15.90612383
1.03E-09	15.48203412
1.35E-16	33.46783843
2.99E-15	29.66789366
1.25E-11	20.22725392
4.71E-08	11.45183836
4.32E-13	23.96275715
2.54E-11	19.45515996
1.10E-12	22.90921698
1.35E-17	36.47831479
1.06E-10	17.91844111
9.08E-14	25.69728184
5.65E-19	41.0356339
5.11E-08	11.36309203
1.99E-12	22.26722337
4.09E-07	9.168745304
1.11E-19	43.58512545
7.59E-11	18.28061402
1.81E-10	17.33777209
1.90E-06	7.563911582
3.24E-10	16.70662673
8.15E-09	13.28227578
5.24E-13	23.73717888
0.031001818	-2.864603277
1.18E-13	25.40229012
1.28E-11	20.2036402
5.23E-12	21.19468106
4.54E-11	18.82699096
9.60E-16	31.001495
1.06E-06	8.178665462
8.37E-13	23.21420614
9.86E-16	30.96128434

2.08E-15	30.11272874
8.89E-13	23.14108653
0.001197257	0.68336655
0.00027763	2.251247573
3.21E-13	24.29588675
0.002460443	-0.090617051
8.23E-10	15.71618405
0.005354794	-0.929466129
1.68E-16	33.11747764
4.04E-16	32.04052721
1.65E-10	17.44158895
2.95E-08	11.94428031
3.71E-10	16.56936273
2.96E-10	16.80562082
1.03E-13	25.55076346
7.84E-09	13.32247943
1.09E-16	33.82545145
3.79E-14	26.70817858
1.06E-14	28.20543325
1.07E-14	28.18789556
1.20E-12	22.81180731
1.46E-17	36.36527535
8.36E-10	15.70067262
2.21E-12	22.14562427
1.70E-05	5.252814473
1.69E-13	25.0034959
3.55E-08	11.74474735
1.35E-14	27.91542625
1.53E-14	27.76206534
2.78E-12	21.90042613
3.22E-12	21.73875629
2.36E-17	35.66549032
8.89E-13	23.14451854
0.000242602	2.398445038
5.75E-15	28.88510875
2.09E-15	30.09700839
2.13E-16	32.8223353
4.21E-08	11.56880697
8.44E-12	20.67961901
4.78E-18	37.83964319
1.27E-09	15.26147466
1.09E-12	22.92811229
1.66E-10	17.43219988
1.11E-13	25.4663645
3.00E-07	9.496958913
3.71E-18	38.12596613
1.02E-13	25.57015791
2.07E-13	24.76889566
2.02E-10	17.21678953
4.13E-07	9.160137805
3.38E-14	26.83760701
3.27E-05	4.547547289
9.40E-09	13.13644968

3.88E-15	29.36568545
2.37E-15	29.95841424
1.03E-11	20.45646909
1.09E-05	5.715314776
2.46E-11	19.49126663
1.24E-12	22.77748757
2.37E-15	29.94772033
1.24E-18	39.50550029
2.34E-17	35.70593151
0.020157106	-2.388026041
3.58E-15	29.45445891
1.34E-05	5.500262819
0.000449182	1.733597389
2.07E-13	24.76777187
4.81E-15	29.0937094
1.48E-06	7.827664351
8.08E-14	25.83138325
4.12E-06	6.756494436
2.87E-19	41.8259816
1.45E-11	20.07127441
1.97E-09	14.80213652
1.79E-13	24.93282235
7.25E-18	37.25517739
6.70E-15	28.69964261
7.45E-06	6.120381698
1.24E-18	39.47637276
1.64E-13	25.04242103
8.25E-14	25.80501242
5.54E-15	28.93612822
2.18E-09	14.69548813
2.00E-08	12.34910884
4.72E-08	11.44766808
1.45E-14	27.8367719
2.89E-18	38.47601388
2.08E-14	27.40470096
7.17E-19	40.59731791
1.02E-18	39.98064862
3.32E-13	24.25970476
1.65E-10	17.4383686
8.04E-14	25.8395788
9.67E-11	18.01624893
1.12E-11	20.35622779
1.17E-16	33.72490063
3.75E-15	29.40330394
2.29E-08	12.21020487
8.17E-14	25.81764449
3.53E-13	24.18543423
4.03E-07	9.185683106
6.87E-10	15.90871569
2.46E-11	19.48840123
6.49E-06	6.270675727
5.41E-18	37.5980386
6.28E-14	26.12814205

1.56E-10	17.50060405
1.10E-11	20.37645021
3.54E-11	19.09781006
1.97E-13	24.82311649
8.42E-08	10.8415261
2.99E-15	29.66459578
6.22E-12	21.00015412
2.05E-16	32.8813634
1.41E-16	33.38968619
2.60E-11	19.42954696
4.49E-15	29.17720923
2.28E-09	14.6466318
5.35E-11	18.64607446
5.30E-14	26.33144973
6.26E-16	31.52970292
3.50E-12	21.64417211
2.62E-05	4.785670469
1.19E-07	10.47478693
2.39E-15	29.93028944
1.25E-11	20.23432405
4.02E-15	29.32540935
1.68E-08	12.52932907
1.18E-13	25.39850399
2.89E-18	38.45454011
0.00053746	1.544387121
5.39E-10	16.1638938
1.31E-13	25.27611675
6.03E-09	13.60417309
5.50E-14	26.28204648
1.67E-11	19.92358058
2.76E-14	27.06773696
3.83E-16	32.11022812
1.91E-14	27.49918658
4.01E-12	21.49196201
2.96E-10	16.80844743
4.98E-05	4.09249484
2.48E-12	22.0212776
2.95E-16	32.43782794
1.03E-11	20.45862999
2.08E-14	27.40650659
5.55E-11	18.60690697
4.90E-16	31.79370485
9.55E-13	23.06725449
1.12E-11	20.3617688
1.91E-14	27.50095628
5.73E-13	23.63791434
0.000944905	0.943970506
1.35E-16	33.47011237
3.73E-17	35.13779207
1.53E-14	27.77379368
4.16E-08	11.58263045
0.000148156	2.9270371
4.91E-15	29.06922962

7.84E-05	3.605561147
4.81E-13	23.83283467
3.86E-13	24.08722401
2.64E-15	29.80242512
1.35E-12	22.68842445
3.52E-12	21.63512399
7.31E-16	31.32483868
8.62E-16	31.1280422
7.06E-16	31.39704359
8.31E-18	37.089523
1.02E-05	5.782445814
2.92E-13	24.40244319
6.18E-17	34.52100426
4.95E-15	29.05689512
2.91E-13	24.40617741
2.24E-12	22.12917755
6.41E-15	28.7606317
4.41E-16	31.92128972
5.50E-14	26.28245508
2.41E-16	32.65178397
1.33E-10	17.67418771
3.17E-16	32.34846358
1.36E-10	17.65548647
6.25E-14	26.1392487
7.70E-10	15.78658329
5.41E-18	37.57368703
4.67E-13	23.87229348
1.29E-13	25.29541128
1.59E-10	17.48072416
3.22E-07	9.423029325
3.45E-12	21.65766742
8.33E-18	37.05666244
2.20E-14	27.32749306
1.84E-05	5.162871003
1.29E-13	25.30010465
3.79E-12	21.55652779
3.38E-14	26.8368265
4.24E-15	29.24373035
1.78E-14	27.58848657
7.69E-14	25.88588841
4.29E-09	13.97813226
6.64E-14	26.05213649
1.06E-16	33.88398521
0.016078809	-2.138560044
2.44E-17	35.61023438
4.25E-14	26.5783752
1.72E-17	36.10932023
2.32E-13	24.64965138
3.00E-08	11.92782735
9.58E-12	20.53960936
2.46E-14	27.19348186
1.91E-08	12.39918074
2.24E-11	19.59798362

1.70E-16	33.09333772
8.77E-12	20.63635581
2.27E-17	35.77481619
0.001431972	0.49331536
2.94E-13	24.39241466
1.78E-09	14.90591735
5.04E-11	18.71592469
5.88E-08	11.21331408
5.68E-17	34.63685027
2.91E-13	24.4095019
2.82E-07	9.562110095
1.63E-16	33.1931569
1.64E-16	33.15960531
2.85E-16	32.4786107
1.12E-12	22.88671114
1.44E-11	20.07840451
5.41E-18	37.58575271
6.86E-07	8.631793122
2.11E-11	19.66774243
1.11E-12	22.8971536
4.19E-16	31.98914443
6.56E-12	20.94139642
1.23E-10	17.76068735
2.43E-12	22.04624751
5.48E-13	23.69013282
6.81E-15	28.6737514
3.47E-16	32.2335317
2.15E-09	14.70860231
1.08E-14	28.17545312
6.18E-16	31.55059874
6.88E-08	11.05403437
1.77E-08	12.47710019
4.40E-17	34.92539732
1.09E-13	25.48491711
6.97E-14	25.99307218
2.14E-08	12.28151316
7.87E-16	31.22387678
1.10E-12	22.90832175
0.000242862	2.397198261
4.95E-07	8.972460315
3.95E-09	14.06751584
1.06E-10	17.91474471
1.43E-10	17.60043851
6.60E-15	28.72337328
8.17E-15	28.4602846
9.12E-10	15.6075821
2.15E-09	14.70954937
5.68E-10	16.11213567
9.89E-08	10.67019019
6.15E-06	6.328690908
1.92E-15	30.21940722
1.86E-13	24.88739498
5.57E-15	28.92703278

4.01E-12	21.49105955
1.88E-15	30.25086005
3.17E-16	32.34126718
9.61E-07	8.275406917
1.47E-17	36.33310616
7.14E-12	20.85233505
1.53E-14	27.75939591
3.73E-14	26.72601619
1.07E-14	28.18466341
3.38E-16	32.26601861
1.23E-10	17.76194192
4.77E-13	23.8452744
1.43E-09	15.14069027
9.24E-07	8.316027827
8.18E-20	44.27570711
6.82E-07	8.639204721
4.10E-15	29.29654218
2.51E-07	9.680788098
8.87E-17	34.13547152
2.27E-17	35.76673795
2.86E-08	11.97791473
5.00E-13	23.79200849
3.22E-15	29.57418739
3.06E-14	26.95864082
1.02E-16	33.93160458
0.012860478	-1.890302352
1.07E-12	22.94643975
2.14E-14	27.37340818
1.14E-14	28.10854795
9.64E-12	20.53044246
5.78E-14	26.23103769
3.91E-12	21.52083242
6.61E-15	28.71691829
1.62E-10	17.46199745
4.66E-12	21.32569773
1.59E-09	15.02329239
4.16E-09	14.01185007
1.42E-16	33.35834655
3.61E-16	32.17547412
1.91E-15	30.2315977
1.02E-11	20.47132346
1.19E-16	33.69844133
2.54E-15	29.85471928
2.78E-14	27.05234212
1.35E-14	27.91736401
7.31E-16	31.32512441
3.58E-11	19.08391762
1.24E-18	39.57245706
0.000253773	2.349576928
1.39E-16	33.41457437
6.16E-11	18.50007395
3.31E-11	19.17085547
4.53E-10	16.35170783

3.17E-16	32.33833642
0.004024346	-0.623708116
9.12E-16	31.06543637
4.24E-15	29.24167334
8.95E-11	18.10131432
8.18E-20	44.6005903
1.20E-13	25.37828153
9.27E-19	40.15575532
8.02E-13	23.26145945
0.017511968	-2.231275601
1.35E-16	33.50288724
1.67E-16	33.13410811
1.35E-16	33.47625177
7.69E-09	13.34373879
1.40E-12	22.64672034
1.28E-07	10.39734756
1.14E-11	20.33416614
5.65E-12	21.10654105
5.05E-13	23.7784676
1.79E-13	24.93599835
2.06E-15	30.13157883
4.71E-09	13.87210752
6.56E-12	20.94096315
6.59E-14	26.07188821
2.06E-08	12.31876796
6.67E-14	26.04175223
1.80E-15	30.30516647
2.68E-10	16.91459316
1.47E-12	22.59706472
1.12E-06	8.121364307
2.04E-13	24.78654232
1.10E-10	17.88069339
2.99E-07	9.500841261
8.76E-14	25.73811581
1.81E-09	14.88320626
4.40E-13	23.93535049
5.13E-15	29.0168675
1.38E-11	20.12281804
6.26E-14	26.13441956
0.000458758	1.710487408
3.99E-13	24.04533708
6.12E-12	21.02046135
7.34E-17	34.33665019
1.91E-11	19.77524591
3.71E-18	38.14084361
2.05E-19	42.48465333
4.81E-15	29.09792515
2.40E-13	24.60915451
1.32E-13	25.27012271
3.90E-08	11.64947811
2.02E-12	22.25251004
2.48E-12	22.02283442
4.90E-16	31.79673581

3.26E-06	6.999595349
5.43E-06	6.460373369
3.30E-17	35.29604342
5.05E-14	26.38886255
3.78E-09	14.11299281
2.15E-07	9.853204028
3.88E-13	24.07951429
5.30E-12	21.17759314
2.64E-10	16.93235415
2.65E-11	19.40157106
0.005320026	-0.923319323
8.76E-11	18.12502807
1.94E-08	12.38101277
2.84E-13	24.43616255
1.65E-06	7.708142602
2.14E-14	27.36794655
6.78E-15	28.68212471
1.15E-06	8.090190582
1.24E-18	39.57697202
2.31E-07	9.772765362
1.62E-19	42.92907825
3.93E-07	9.212556528
9.86E-16	30.96670197
3.36E-14	26.85327981
4.27E-17	34.98815207
1.67E-11	19.91840099
3.53E-16	32.20527075
6.48E-09	13.52917659
8.21E-11	18.19233782
6.51E-09	13.52207395
8.89E-13	23.14807363
3.90E-12	21.52638368
3.60E-09	14.16797461
2.75E-06	7.178762381
1.19E-12	22.82404961
3.65E-14	26.75009105
5.96E-12	21.04819113
